# SESAME-catalyzed H3T11 phosphorylation inhibits Dot1-catalyzed H3K79me3 to regulate autophagy and telomere silencing

**DOI:** 10.1038/s41467-022-35182-9

**Published:** 2022-12-06

**Authors:** Fei He, Qi Yu, Min Wang, Rongsha Wang, Xuanyunjing Gong, Feng Ge, Xilan Yu, Shanshan Li

**Affiliations:** 1 State Key Laboratory of Biocatalysis and Enzyme Engineering, School of Life Sciences, Hubei University, Wuhan, Hubei 430062 China; 2 Key Laboratory of Algal Biology, Institute of Hydrobiology, Chinese Academy of Sciences, Wuhan, Hubei 430072 China

**Keywords:** Epigenetics, Autophagy, Epigenetics, Enzymes

## Abstract

The glycolytic enzyme, pyruvate kinase Pyk1 maintains telomere heterochromatin by phosphorylating histone H3T11 (H3pT11), which promotes SIR (silent information regulator) complex binding at telomeres and prevents autophagy-mediated Sir2 degradation. However, the exact mechanism of action for H3pT11 is poorly understood. Here, we report that H3pT11 directly inhibits Dot1-catalyzed H3K79 tri-methylation (H3K79me3) and uncover how this histone crosstalk regulates autophagy and telomere silencing. Mechanistically, Pyk1-catalyzed H3pT11 directly reduces the binding of Dot1 to chromatin and inhibits Dot1-catalyzed H3K79me3, which leads to transcriptional repression of autophagy genes and reduced autophagy. Despite the antagonism between H3pT11 and H3K79me3, they work together to promote the binding of SIR complex at telomeres to maintain telomere silencing. Furthermore, we identify Reb1 as a telomere-associated factor that recruits Pyk1-containing SESAME (Serine-responsive SAM-containing Metabolic Enzyme) complex to telomere regions to phosphorylate H3T11 and prevent the invasion of H3K79me3 from euchromatin into heterochromatin to maintain telomere silencing. Together, these results uncover a histone crosstalk and provide insights into dynamic regulation of silent heterochromatin and autophagy in response to cell metabolism.

## Abstract (web summary)

How H3T11 phosphorylation exerts biological functions remains poorly understood. Here, authors show that H3pT11 directly inhibits Dot1-catalyzed H3K79 tri-methylation (H3K79me3) and uncover how this histone crosstalk regulates autophagy and telomere silencing.

## Introduction

In the nucleus, the eukaryotic genome is organized into actively transcribed gene-rich euchromatin and condensed gene-poor silent heterochromatin. In budding yeast, heterochromatin is localized at three genome locations, including telomeres, silent mating-type loci, and ribosomal DNA (rDNA) repeats, which comprise ~10% of the yeast genome^1^. The transcription of genes located proximal to these heterochromatic regions is repressed or silenced, a phenomenon known as position effect variegation (PEV)^2,3^. Maintenance of the integrity of silent heterochromatin is required for chromosome stability, including telomere protection, chromosome segregation fidelity, transposon repression, and DNA damage repair^4–6^.

The silent heterochromatin at yeast telomeres is formed by assembly and spread of a silent information regulator (SIR) complex, which composed of Sir2, Sir3, and Sir4 at chromatin, among which Sir2 is a NAD^+^-dependent histone deacetylase with preference for H4K16ac. Telomere heterochromatin formation is initiated by recruitment of Sir2 and Sir4 by Rap1 and the yeast Ku (yKu) complex to *cis*-acting silencer elements, where Sir2 deacetylates H4K16 on nucleosomes^7^. Deacetylated H4K16 is bound by Sir3, which in turn recruits more Sir2 and Sir4^8^. The propagation of SIR complex along the telomeres protects the underlying genes from transcription machinery, leading to their repression, so called telomere silencing^9–11^. The spread of SIR complex to transcriptionally active chromatin is antagonized by active histone markers, including Sas2-catalyzed H4K16ac, Dot1-catalyzed H3K79me3, and Set1-catalyzed H3K4me3, which prevent SIR complex titration away from heterochromatin into euchromatin regions and keep the concentration of SIR complex high at telomeres^12–15^. Notably, these modifications occur predominantly in euchromatin regions and are absent from telomere heterochromatin regions, suggesting that they indirectly regulate heterochromatin structure^16^. Although Sir2 deacetylates H4K16 and Sir3 competes with Dot1 for nucleosomes^17,18^, the mechanism to prevent the occurrence of these histone markers, especially H3K79me3, at silent heterochromatin is not completely understood.

Some metabolic enzymes have been reported to translocate into the nucleus, where they regulate telomere silencing. For example, glyceraldehyde 3-phosphate dehydrogenase (Tdh3) and glutamate dehydrogenase 1 (Gdh1) can translocate into the nucleus and maintain telomere silencing^19,20^. We have previously reported that the glycolytic enzyme, pyruvate kinase (Pyk1) can translocate into the nucleus, where it phosphorylates histone H3T11 (H3pT11) as a catalytic subunit of SESAME complex (Serine-responsive SAM-containing Metabolic Enzyme)^21^. The activity of SESAME is stimulated by glycolysis and serine metabolism to repress gene expression and confer cell resistance to oxidative stress^21,22^. Moreover, SESAME phosphorylates H3T11 at telomere regions to maintain telomere silencing by promoting SIR complex binding at telomeres and preventing autophagy-mediated Sir2 degradation^23^. This study also suggests that autophagy may affect telomere silencing by degrading telomere-associated proteins. However, little is known about the proteins and/or protein domains that specifically bind H3pT11 and the exact mechanism of action for H3pT11 remains poorly understood. For example, how H3pT11 represses autophagy (*ATG*) gene expression remains unknown. Moreover, it remains unclear how SESAME is recruited to telomere regions. In this work, we identify SESAME-catalyzed H3pT11 as an upstream inhibitor of Dot1-catalyzed H3K79me3. By inhibiting Dot1-catalyzed H3K79me3, H3pT11 represses the transcription of *ATG* genes and prevents autophagy. Moreover, H3pT11 and H3K79me3 work together to enhance the binding of SIR complex at telomeres to maintain telomere silencing. In addition, SESAME is recruited by Reb1 to phosphorylate H3T11 at telomeres and restrict H3K79me3 at the heterochromatin-euchromatin boundary to maintain telomere silencing.

## Results

### H3T11 phosphorylation anti-correlates with H3K79me3

**Fig. 1:**
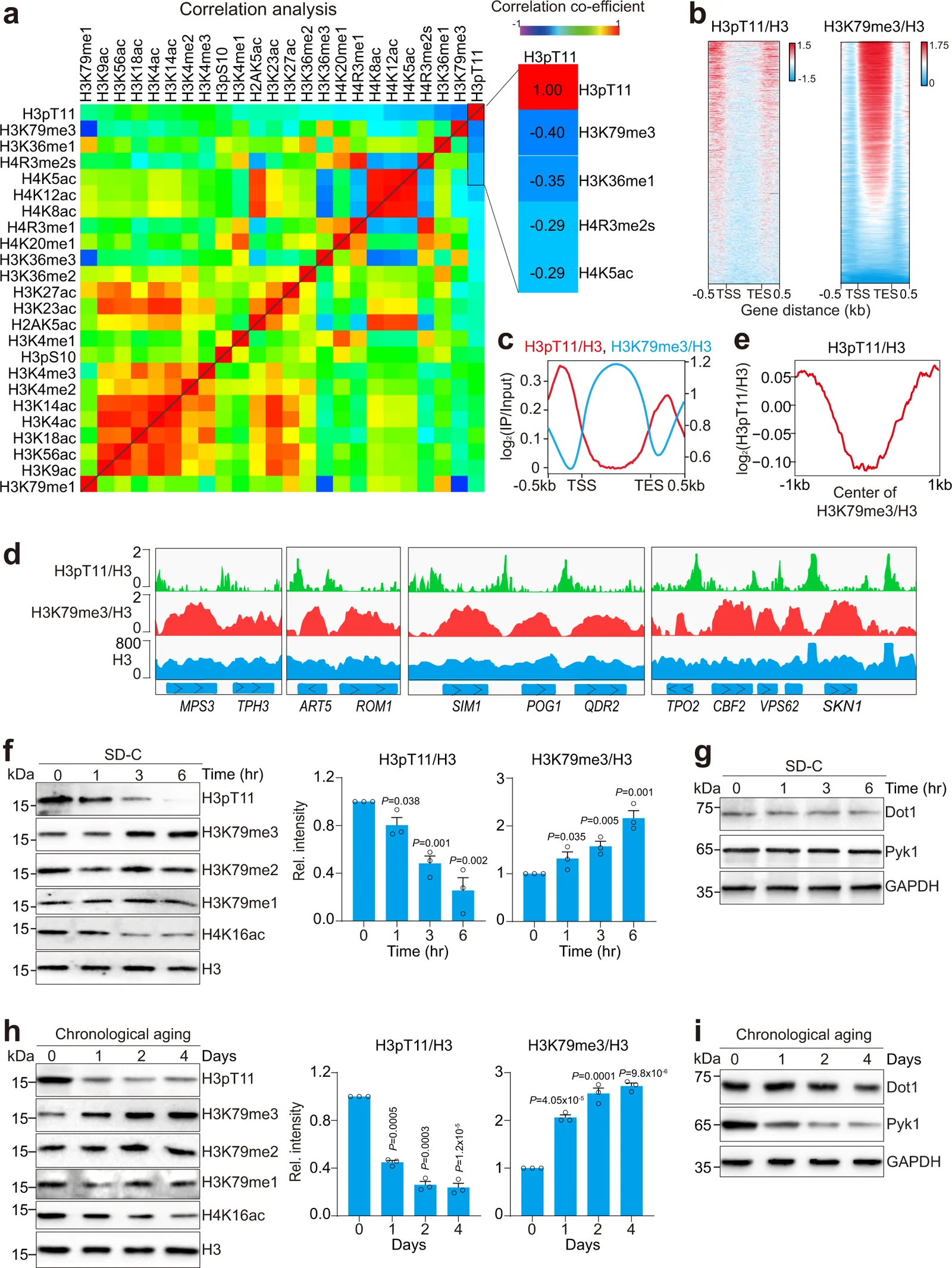
H3pT11 anti-correlates with H3K79me3. **a** Correlation analyses of the ChIP-seq data of H3pT11 with other histone modifications in budding yeast. The correlation of each histone mark to itself along the diagonal is defined as 1.0, which is indicated in black line. **b** Distribution of H3pT11 and H3K79me3 across each gene through 1 kb upstream of the TSS to 1 kb downstream from the TES at all genes. Log_2_ ratios of H3pT11 or H3K79me3 versus H3 at significantly enriched windows were used. Each row represents one gene. **c** Averaged metagene profiles of H3pT11/H3 and H3K79me3/H3 from **a**. **d** ChIP-seq tracks showing the enrichment of H3pT11/H3 and H3K79me3/H3 at representative genes. **e** Averaged distribution of H3pT11/H3 (log_2_ (H3pT11/H3)) around the peaks of H3K79me3/H3. **f** Immunoblots of H3pT11 and H3K79me3 when cells were grown in glucose starvation conditions (SD-C). Cells were grown in YPD until OD_600_ of 1.0. Cells were harvested, resuspended in SD-C and then grown for 0–6 h. **g** Immunoblots of Dot1 and Pyk1 in cells when grown in glucose starvation conditions (SD-C) for 6 h. **h** Immunoblot analysis of H3pT11 and H3K79me3 when cells were grown in YPD medium for 0–4 days. **i** Immunoblots of Dot1 and Pyk1 when cells were grown in YPD medium for 0–4 days. For **f**, **h**, data represent the mean ± SE; *n* = 3 biological independent experiments. Two-sided *t*-tests were used for statistical analysis. For **g**, **i**, a typical example of three biologically independent replicates was shown. Source data are provided with this paper.

To identify histone modifications regulated by H3T11 phosphorylation (H3pT11), we compared the ChIP-seq data for H3pT11 and the ChIP-seq datasets for other known histone modifications^23–25^. As shown in Fig. 1a, H3pT11 displayed strong anti-correlation with H3K79 trimethylation (H3K79me3) with a correlation co-efficient of −0.40 (Fig. 1a). In contrast, no anti-correlation was observed between H3pT11 and H3K79me1 (Fig. 1a). Our heat map and metagene analysis indicated that H3pT11 and H3K79me3 could be mutually exclusive (Fig. 1b, c). H3pT11 is located upstream of the transcription start site (TSS) and downstream from the transcription end site (TES) but is absent at gene coding regions (Fig. 1b–d). However, H3K79me3 predominantly occurred at gene coding regions but not at TSS and TES regions (Fig. 1b–d). When we centered all H3K79me3 peaks and plotted the levels of H3pT11, we found that the H3pT11 enrichment signals anti-correlate with the ChIP-seq signals of H3K79me3 genome-wide (Fig. 1e).

Pyk1-catalyzed H3pT11 is regulated by glucose availability^23^. When cells were grown in glucose starvation (SD-C) medium, the global H3pT11 was gradually reduced and H3K79me3 was gradually increased (Fig. 1f). In contrast, H3K79me1 and H3K79me2 showed no significant changes (Fig. 1f). The expression of Pyk1 and Dot1, which catalyze H3pT11 and H3K79me3, respectively, was not significantly changed when grown under glucose starvation conditions (Fig. 1g). H3pT11 is regulated by glycolytic enzymes, such as phosphoglucose isomerase (Pgi1), enolase (Eno2) and phosphofructose kinase (Pfk2)^22^. As *PGI1* and *ENO2* are essential genes, we thus examined H3pT11 and H3K79me3 in *TetO*_*7*_*-PGI1* and *TetO*_*7*_*-ENO2* mutants, where *PGI1* and *ENO2* promoters were replaced with TetO_7_ and their transcription can be shut off by doxycycline treatment^26^ (Supplementary Fig. 1a, b). Indeed, H3pT11 was significantly reduced and H3K79me3 was significantly increased in *TetO*_*7*_*-PGI1* and *TetO*_*7*_*-ENO2* mutants (Supplementary Fig. 1a, b). Moreover, H3pT11 was significantly reduced and H3K79me3 was increased in *pfk2Δ* mutants (Supplementary Fig. 1c).

H3pT11 has also been reported to be reduced during chronological aging^23^. When cells were grown in YPD medium for 0–4 days to undergo chronological aging, the intracellular H3pT11 was significantly reduced and H3K79me3 was significantly increased (Fig. 1h). The transcription of *PYK1* was reduced during chronological aging (Supplementary Fig. 1d), which may lead to reduced H3pT11. Nonetheless, the expression of Pyk1 and Dot1 was not oppositely regulated during chronological aging process (Fig. 1i).

We also examined whether this anti-correlation relationship between H3pT11 and H3K79me3 exists in other processes including methyl methanesulfonate (MMS)-triggered DNA damage response, hydroxyurea (HU)-caused DNA replication block, and different cell cycle phases. However, H3pT11 was not significantly changed in these processes and no anti-correlation was observed between H3pT11 and H3K79me3 (Supplementary Fig. 1e-g).

### Loss of SESAME-catalyzed H3pT11 increases H3K79me3

**Fig. 2:**
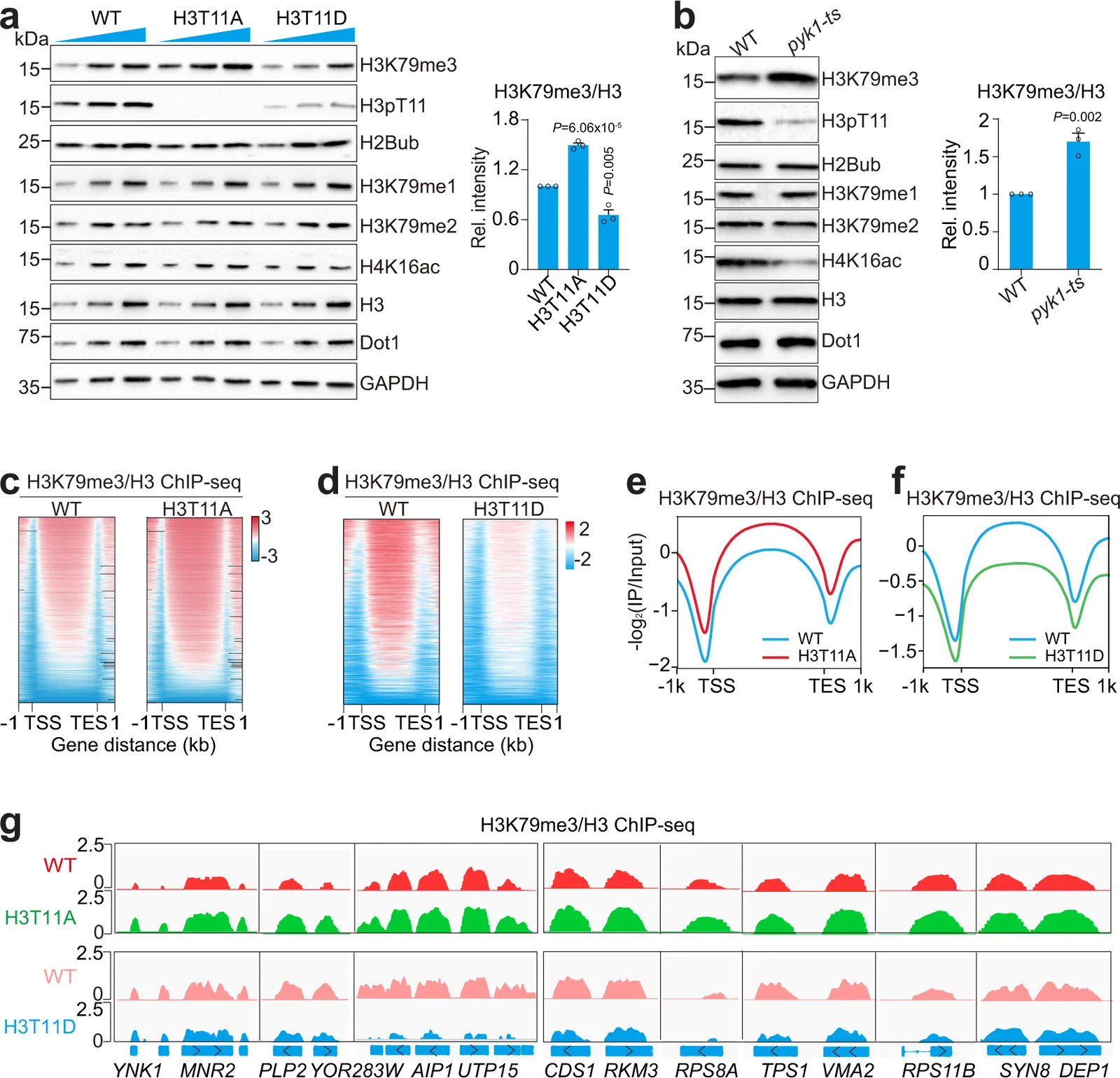
Loss of SESAME-catalyzed H3pT11 increases Dot1-catalyzed H3K79me3. **a** Immunoblot analysis of histone modifications and Dot1 in WT, H3T11A and H3T11D mutants when grown in YPD medium until OD_600_ of 1.0. **b** Immunoblot analysis of histone modifications and Dot1 in WT and *pyk1-ts* mutants when grown in YPD medium at non-permissive temperature (39 °C) for 2 h. **c** Distribution of H3K79me3 across each gene through 1 kb upstream of the TSS to 1 kb downstream from the TES at all genes in WT and H3T11A mutant. **d** Distribution of H3K79me3 across each gene through 1 kb upstream of the TSS to 1 kb downstream from the TES at all genes in WT and H3T11D mutant. **e** Averaged metagene profiles of H3K79me3/H3 in WT and H3T11A mutant. **f** Averaged metagene profiles of H3K79me3/H3 in WT and H3T11D mutant. **g** ChIP-seq tracks showing the enrichment of H3K79me3/H3 at representative genes in WT, H3T11A, and H3T11D mutants. For Fig. **a**, **b**, data represent the mean ± SE; *n* = 3 biologically independent experiments. Two-sided *t*-tests were used for statistical analysis. Source data are provided with this paper.

Considering the strong correlation between H3K79me3 and gene transcription^27^ as well as the anti-correlation between H3pT11 and H3K79me3, we examined whether H3pT11 regulates gene transcription via H3K79me3. The global level of H3K79me3 but not H3K79me1 and H3K79me2 was significantly increased in H3T11A mutant, whereas H3K79me3 was significantly reduced in H3T11D mutant, which mimics H3pT11 (Fig. 2a; Supplementary Fig. 2a). As Pyk1 within the SESAME complex phosphorylates H3T11, we then examined the effect of SESAME complex on H3K79me3. H3pT11 was significantly reduced and H3K79me3 was significantly increased in SESAME mutants, including *pyk1-ts* and *acs2-ts* mutants at non-permissive temperature 39 °C (Fig. 2b; Supplementary Fig. 2b).

Dot1 has been reported to methylate histones assembled into chromatin^14^. We thus examined whether this inhibitory effect occurred at chromatin. By subcellular fractionation, we found that the chromatin-bound H3pT11 was significantly reduced in *pyk1-ts* mutant and the chromatin-bound H3K79me3 was significantly increased in *pyk1-ts* mutant (Supplementary Fig. 2c, lane 3 vs lane 4). To investigate the effect of H3pT11 on genome-wide occupancy of H3K79me3, we performed ChIP-seq (chromatin immunoprecipitation combined with high-throughput sequencing) for H3K79me3 in WT, H3T11A, and H3T11D mutants. The occupancy of H3K79me3 at chromatin was significantly increased in H3T11A mutant and reduced in H3T11D mutant when compared with WT (Fig. 2c–g; Supplementary Fig. 2d). We further inhibited the nuclear transport of Pyk1 using the “anchor-away” technique, which a domain of FK506-rapamycin-binding protein (FRB) is fused to Pyk1 (Pyk1-FRB), and upon rapamycin treatment is trapped in the cytoplasm via an interaction with an FKBP-ribosomal protein fusion^28^. When Pyk1-FRB was treated with rapamycin, the amount of Pyk1 in the nucleus was markedly reduced (Supplementary Fig. 3a). Blocking the nuclear translocation of Pyk1 significantly reduced the global H3pT11 and increased the global H3K79me3 (Supplementary Fig. 3b). We also performed ChIP-seq for H3pT11 and H3K79me3 in Pyk1-FRB when treated with or without rapamycin. It revealed that depletion of nuclear Pyk1 significantly reduced the genome-wide occupancy of H3pT11 and increased the genome-wide occupancy of H3K79me3 (Supplementary Fig. 3c, d), suggesting that loss of H3T11 phosphorylation increased genome-wide occupancy of H3K79me3.

### SESAME-catalyzed H3pT11 directly inhibits the catalytic activity of Dot1 on nucleosomes

To understand how H3pT11 inhibits H3K79me3, we first examined the effect of H3pT11 on Dot1 expression. The protein levels of Dot1 were not significantly changed in both H3T11A and *pyk1-ts* mutants (Fig. 2a, b; Supplementary Fig. 2a). Dot1-catalyzed H3K79me3 has been reported to be regulated by histone modifications, including Rad6/Bre1-catalyzed H2BK123 monoubiquitination (H2Bub)^29^ and H4K16 acetylation (H4K16ac)^30^. H2Bub and H4K16ac were not increased in H3T11A mutant (Fig. 2a). Surprisingly, although the global H3K79me3 was significantly increased in *pyk1-ts* mutant, the global H4K16ac was reduced in *pyk1-ts* mutant (Fig. 2b). The reduced H4K16ac could be caused by reduced production of acetyl-CoA within the context of SESAME complex and impaired function of H4K16 acetyltransferase SAS complex^31^. We thus examined the genome-wide occupancy of H4K16ac in WT and *pyk1-ts* mutant by ChIP-seq. H4K16ac was increased at subtelomere regions in *pyk1-ts* mutant (Supplementary Fig. 3e, f), which is consistent with the role of H4K16ac in promoting H3K79me3^30^. In other places like non-subtelomere regions, the occupancy of H4K16ac was decreased in *pyk1-ts* mutant (Supplementary Fig. 3g). As H3T11 phosphorylation is required to maintain the binding of H4K16 deacetylase Sir2 at subtelomere regions^23^, the increased H4K16ac at subtelomere regions in *pyk1-ts* mutant could be due to reduced Sir2.

**Fig. 3:**
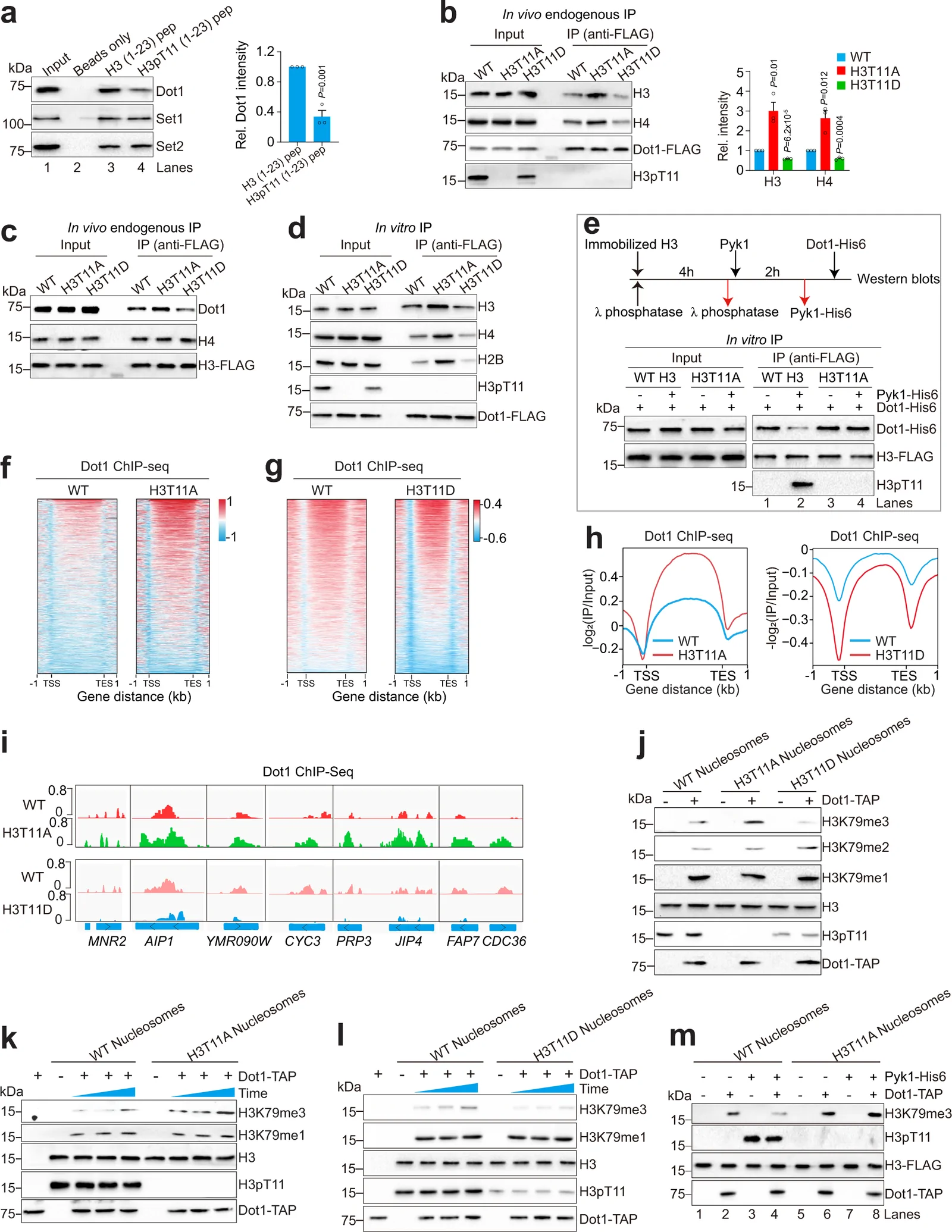
SESAME-catalyzed H3pT11 antagonizes Dot1-catalyzed H3K79me3. **a** Peptide pull-down assay showing Dot1 had reduced binding to H3pT11 peptide. The biotinylated H3 (1–23) peptide and H3pT11 (1–23) peptide were incubated with the whole cell extract. **b** In vivo endogenous Co-IP showing Dot1 preferentially bound H3T11A nucleosomes but bound less H3T11D nucleosomes. **c** Reciprocal IP showing Dot1 preferentially bound H3T11A nucleosomes. Nucleosomes (H3-FLAG) were immunoprecipitated from WT, H3T11A, and H3T11D mutants with anti-FLAG beads. **d** In vitro Co-IP showing purified Dot1-FLAG preferentially bound in vitro assembled H3T11A nucleosomes. Although the recombinant histone H3 was expressed and purified from *E. coli*, it was phosphorylated at H3T11 possibly by one of the two forms of *E. coli* pyruvate kinase. Thus, the in vitro-assembled nucleosomes were phosphorylated at H3T11. **e** In vitro Co-IP showing Pyk1-catalyzed H3pT11 inhibits the interaction between Dot1 and nucleosomes. WT H3-FLAG-containing nucleosomes were isolated from yeast cells and immobilized on anti-FLAG beads. The immobilized nucleosomes were dephosphorylated by λ phosphatase and then phosphorylated by purified Pyk1-His6. The nucleosomes were then incubated with purified recombinant Dot1-His6. **f**–**g** Distribution of Dot1 across each gene through 1 kb upstream of the TSS to 1 kb downstream from the TES at all genes in WT, H3T11A, and H3T11D mutants. **h** Averaged metagene profiles of Dot1 ChIP-seq in WT, H3T11A, and H3T11D mutants. **i** ChIP-seq tracks showing Dot1 occupancy at representative genes in WT, H3T11A and H3T11D mutants. **j**–**l** In vitro histone methyltransferase assay with purified Dot1 (Dot1-TAP) and nucleosomes purified from yeast cells (**j**), or in vitro assembled nucleosomes (**k**, **l**) showing that Dot1 has higher H3K79me3 activity to H3T11A nucleosomes and lower activity to H3T11D nucleosomes. **m** In vitro histone methyltransferase assay with purified Dot1 (Dot1-TAP) and nucleosomes phosphorylated by Pyk1 showing that Pyk1-catalyzed H3pT11 inhibits Dot1-catalyzed H3K79me3. For **a**, **b**, data represent the mean ± SE; *n* = 3 biological independent experiments. Two-sided *t*-tests were used for statistical analysis. For **c**–**e**, **j**–**m**, a typical example of at least two biological independent replicates was shown. Source data are provided with this paper.

We then investigated the effect of SESAME-catalyzed H3pT11 on the binding of Dot1 to its histone substrates. We performed the peptide pull-down assay by incubating cell extracts with biotinylated unmodified, or H3T11 phosphorylated (H3pT11) H3 (1-23) peptides. Less Dot1 was pulled down by H3pT11 (1-23) peptide (Fig. 3a, lane 3 vs lane 4). For the other two histone methytransferases, Set1 and Set2, which are responsible for H3K4 methylation and H3K36 methylation, respectively, their binding to H3 (1-23) peptides was unaffected by H3T11 phosphorylation (Fig. 3a). We then examined the effect of H3pT11 on Dot1 binding to nucleosomes by co-immunoprecipitation (Co-IP) assay. The endogenous Dot1-FLAG was immunoprecipitated from the cell extracts of WT, H3T11A, and H3T11D mutants, whose chromatin was digested by Micrococcal Nuclease (MNase) to mononucleosomes (Supplementary Fig. 4a). The bound nucleosomes were detected by immunoblots with anti-H3 and anti-H4 antibodies. The co-IPed nucleosomes were significantly increased in H3T11A mutant but significantly reduced in H3T11D mutant (Fig. 3b). Dot1-FLAG bound nucleosomes contain no H3pT11 (Fig. 3b). We also performed the reciprocal IP by immunoprecipitating WT H3-FLAG, H3T11A-FLAG and H3T11D-FLAG nucleosomes with anti-FLAG antibody. It revealed that more Dot1 was pulled down by H3T11A nucleosomes and less Dot1 was pulled down by H3T11D nucleosomes compared with WT nucleosomes (Fig. 3c). We then performed in vitro Co-IP by incubating purified Dot1-FLAG with purified recombinant WT H3, H3T11A and H3T11D histones. Dot1 showed preferential binding towards H3T11A histones and reduced binding affinity towards H3T11D histones when compared with WT H3 (Supplementary Fig. 4b). In contrast, the purified Set1-FLAG had no preference towards WT, H3T11A, and H3T11D histones (Supplementary Fig. 4c). In addition, the in vitro Co-IP with purified Dot1-FLAG and in vitro-assembled nucleosomes showed that Dot1 bound more H3T11A nucleosomes and less H3T11D nucleosomes than WT nucleosomes (Fig. 3d). Still, the purified Set1 had no preference towards WT, H3T11A and H3T11D nucleosomes (Supplementary Fig. 4d).

To directly show that H3pT11 inhibits the binding of Dot1 to nucleosomes, we prepared nucleosomes that were phosphorylated by Pyk1 in vitro and then performed the in vitro Co-IP (Fig. 3e). Specifically, H3-FLAG-containing nucleosomes were isolated from yeast cells and immobilized on anti-FLAG beads. The immobilized nucleosomes were first dephosphorylated by λ phosphatase and then phosphorylated by purified Pyk1-His6. After Pyk1-His6 was washed away, the nucleosomes were then incubated with purified recombinant Dot1-His6. The H3T11A nucleosomes were used as a negative control. The Co-IP assay revealed that Pyk1-catalyzed H3T11 phosphorylation reduced the interaction between Dot1 and nucleosomes (Fig. 3e, lane 1 vs lane 2), while Dot1 showed high and constant binding to H3T11A nucleosomes (Fig. 3e, lane 3 vs lane 4).

We then performed ChIP-seq to examine the effect of H3T11 phosphorylation on the genome-wide occupancy of Dot1 at chromatin. The overall binding intensity of Dot1 at chromatin was significantly increased in H3T11A mutant and significantly reduced in H3T11D mutant when compared with WT (Fig. 3f–i; Supplementary Fig. 4e, f), consistent with increased H3K79me3 in H3T11A mutant and reduced H3K79me3 in H3T11D mutant (Fig. 2c–g). All these data indicate that H3T11A promotes the binding of Dot1 to nucleosomes and H3T11D reduces the binding of Dot1 to nucleosomes.

To examine the effect of H3pT11 on the enzymatic activity of Dot1, we performed in vitro histone methyltransferase (HMT) assay with Dot1 and nucleosomes (WT, H3T11A, H3T11D) purified from yeast cells. Dot1 showed higher H3K79me3 activity towards H3T11A nucleosomes and lower H3K79me3 activity towards H3T11D nucleosomes when compared with WT nucleosomes (Fig. 3j). Dot1 showed similar H3K79me1 activity towards WT, H3T11A and H3T11D nucleosomes (Fig. 3j). As a control, the activity of Set1 to catalyze H3K4me3 was unaffected by H3T11A and H3T11D mutations (Supplementary Fig. 4g). Similar results were observed when in vitro-assembled recombinant WT, H3T11A and H3T11D nucleosomes were used as substrates (Fig. 3k, l). To directly show that it is Pyk1-catalyzed H3T11 phosphorylation that inhibits Dot1-catalyzed H3K79me3, we prepared nucleosomes that were phosphorylated by Pyk1 in vitro and then performed the HMT assay with purified Dot1. Our data showed that Pyk1-phosphorylated nucleosomes directly inhibit Dot1-catalyzed H3K79me3 (Fig. 3m, lane 2 vs lane 4), while the activity of Dot1 towards H3T11A nucleosomes was constant and high even after incubating with Pyk1 (Fig. 3m, lane 6 vs lane 8). Collectively, these data indicate that SESAME-catalyzed H3T11 phosphorylation directly inhibits the binding of Dot1 to chromatin and impairs its activity to catalyze H3K79me3.

### SESAME-catalyzed H3pT11 inhibits Dot1-catalyzed H3K79me3 to repress autophagy

**Fig. 4:**
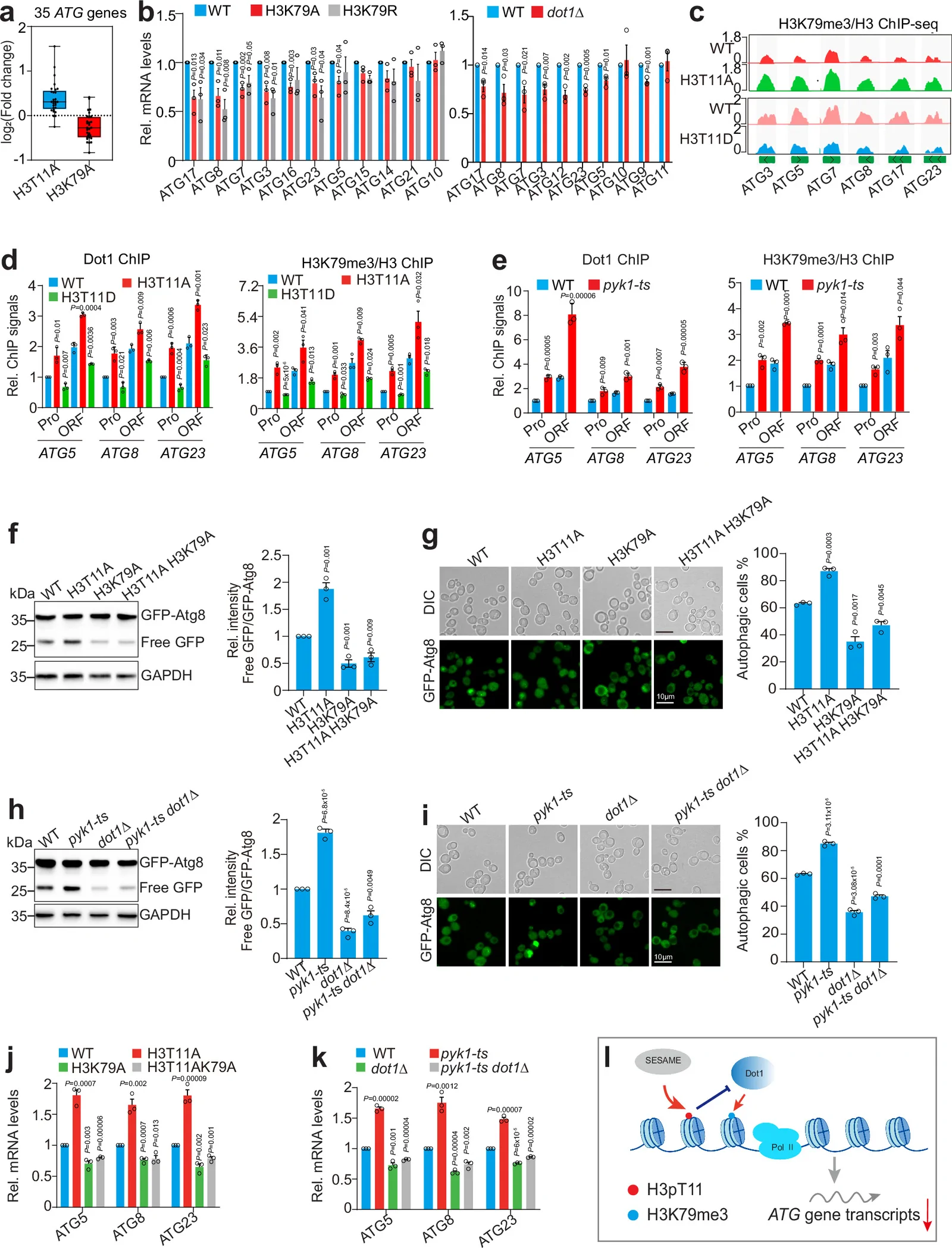
SESAME-catalyzed H3pT11 represses autophagy by inhibiting Dot1-catalyzed H3K79me3. **a** Box plots showing transcriptional changes assayed by RNA-seq of 35 *ATG* genes in H3T11A and H3K79A mutants. **b** RT-qPCR analysis of the transcription of *ATG* genes in WT, H3K79A, H3K79R, and *dot1Δ* mutants. **c** ChIP-seq tracks showing the enrichment of H3K79me3/H3 at representative *ATG* genes in WT, H3T11A, and H3T11D mutants. **d** ChIP-qPCR analysis of the enrichment of Dot1 and H3K79me3/H3 at *ATG5*, *ATG8* and *ATG23* in WT, H3T11A and H3T11D mutants. **e** ChIP-qPCR analysis of Dot1 and H3K79me3 occupancy at *ATG5*, *ATG8* and *ATG23* in WT and *pyk1-ts* mutant when grown in YPD medium at 39 °C for 2 h. **f** Representative immunoblots of GFP-Atg8 and free GFP in WT, H3T11A, H3K79A, and H3T11A H3K79A mutants expressing the endogenous *ATG8* promoter-driven *GFP-ATG8* with anti-GFP antibody. GAPDH was used as a loading control. **g** Representative fluorescence microscopy images showing the distribution of GFP-Atg8 (green) in WT, H3T11A, H3K79A, and H3T11A H3K79A mutants. The autophagic cells were defined as cells with clear vacuolar GFP fluorescence. Quantification of autophagic cells depicted in the right panel with 200–300 counts (blinded) for each replicate. A total of three biologically independent experiments were performed. **h** GFP-Atg8 processing assays were performed in WT, *pyk1-ts*, *dot1Δ* and *pyk1-ts dot1Δ* mutants expressing the endogenous *ATG8* promoter-driven *GFP-ATG8*. **i** Representative fluorescence microscopy images showing the distribution of GFP-Atg8 (green) in WT, *pyk1-ts*, *dot1Δ* and *pyk1-ts dot1Δ* mutants. A total of three biological independent experiments were performed. **j** RT-qPCR analysis of the transcription of *ATG5*, *ATG8* and *ATG23* in WT, H3T11A, H3K79A, and H3T11A H3K79A mutants. **k** RT-qPCR analysis of the transcription of *ATG5*, *ATG8,* and *ATG23* in WT, *pyk1-ts*, *dot1Δ,* and *pyk1-ts dot1Δ* mutants. **l** Diagram showing SESAME-catalyzed H3T11 phosphorylation represses *ATG* gene transcription by inhibiting Dot1-catalyzed H3K79me3. For Fig. **a**, centre lines denote medians, box limits denote 25th and 75th percentiles and whiskers denote maximum and minimum values. For **a**-**k**, data represent the mean ± SE; *n* = 3 biological independent experiments. Two-sided *t*-tests were used for statistical analysis. Source data are provided with this paper.

We have previously reported that H3pT11 inhibits autophagy by repressing the transcription of autophagy (*ATG*) genes^23^. The expression of *ATG* genes correlates with the formation of autophagosome and transcriptional regulation of *ATG* genes represents an important way to control autophagy^32,33^. However, it remains unclear how H3pT11 represses *ATG* gene transcription. By analyzing the RNA-seq data for H3T11A and H3K79A mutants, we found that the overall transcription of *ATG* genes was increased in H3T11A but reduced in H3K79A mutant (Fig. 4a). We also confirmed the increased transcription of *ATG* genes in H3T11A and *pyk1-ts* mutants by RT-qPCR (Supplementary Fig. 5a, b). The transcription of *ATG* genes was also significantly increased when the nuclear Pyk1 was depleted in Pyk1-FRB mutants by rapamycin (Supplementary Fig. 5c). In contrast, the transcription of *ATG* genes was significantly reduced in H3K79A, H3K79R, and *dot1Δ* mutants (Fig. 4b), indicating that H3pT11 and H3K79me3 have opposite effects on autophagy gene transcription.

We then compared the effect of H3T11A and H3K79A on autophagy activity using a GFP liberation assay, which detects free GFP that is cleaved from the endogenous promoter-driven Atg8 with an N-terminal GFP tag (GFP-Atg8) in the vacuole^23^. As expected, H3T11A and *pyk1-ts* mutants had increased autophagy flux as assessed by increased ratio of free GFP/GFP-Atg8 (Supplementary Fig. 5d, e). Blocking the nuclear transportation of Pyk1 also increased autophagy activity (Supplementary Fig. 5f). The induced autophagy activity was not caused by inactivation of TOR (target of rapamycin) pathway by rapamycin as WT (HHY168) and Pyk1-FRB mutant contain a mutated *TOR1* (*tor1-1*) and deleted *FPR1* (Δ*fpr1*)^28^. In contrast, H3K79A and *dot1Δ* mutants had reduced autophagy flux as indicated by decreased ratio of free GFP/GFP-Atg8 (Supplementary Fig. 5g, h). To confirm these findings, we used a complementary assay by assessing the autophagy-dependent translocation of GFP-Atg8 to the vacuole by fluorescence microscopy. The percentage of autophagic cells that displayed clearly vacuolar localization of GFP was significantly increased in H3T11A but was significantly reduced in H3K79A (Supplementary Fig. 5i), indicating that H3pT11 and H3K79me3 have opposite effects on autophagy activity.

As H3pT11 inhibits Dot1-catalyzed H3K79me3, we next examined the effect of H3T11A and H3T11D on the occupancy of Dot1 and H3K79me3 at *ATG* genes. By analyzing our ChIP-seq data, we found that H3K79me3 was increased at *ATG* genes in H3T11A mutant and decreased in H3T11D mutant (Fig. 4c). ChIP-qPCR also showed that the occupancy of Dot1 and H3K79me3 at *ATG* genes were significantly increased in H3T11A mutant and significantly reduced in H3T11D mutant (Fig. 4d). In accord with these data, the occupancy of Dot1 and H3K79me3 at *ATG* genes was significantly increased in *pyk1-ts* mutant (Fig. 4e), indicating that Pyk1-catalyzed H3pT11 antagonizes Dot1-catalyzed H3K79me3 at *ATG* genes.

To test whether H3pT11 represses autophagy by inhibiting Dot1-catalyzed H3K79me3, we constructed H3T11A H3K79A double mutant. Compared with WT, the autophagy activity was significantly increased in H3T11A mutant, but reduced in H3T11A H3K79A mutants (Fig. 4f, g). Similar effect was observed for *pyk1-ts dot1*Δ double mutant (Fig. 4h, i). We also examined the transcription of *ATG* genes in WT, H3T11A, H3K79A and H3T11A H3K79A mutants by RT-qPCR. While the transcription of *ATG5*, *ATG8* and *ATG23* was significantly increased in H3T11A mutant, mutation of H3K79A restored the up-regulated transcription of *ATG* genes in H3T11A mutant (Fig. 4j). Similarly, deletion of *DOT1* also rescued the increased transcription of *ATG* genes in *pyk1-ts* mutant (Fig. 4k).

Although H3pT11 also showed anti-correlation relationship with H3K36me1 (correlation co-efficient of −0.35) (Fig. 1a), loss of Set2, the sole enzyme that catalyzes H3K36 methylation, had no significant effect on autophagy (Supplementary Fig. 5j). Meanwhile, the transcription of *ATG* genes was not significantly reduced in *set2Δ* mutant (Supplementary Fig. 5k). Some *ATG* genes were even increased in *set2Δ* mutant, including *ATG3*, *ATG5*, *ATG8*, *ATG9* (Supplementary Fig. 5k). It is unlikely that H3T11 phosphorylation represses the transcription of autophagy genes by inhibiting Set2-catalyzed H3K36me1.

SESAME complex has been reported to be recruited to actively transcribed genes by the histone methyltransferase Set1^21^. Indeed, loss of Set1 led to reduced Pyk1 and H3pT11 at *ATG* genes, which results in increased Dot1 and H3K79me3 at *ATG* genes (Supplementary Fig. 5l). Together, these data indicate that SESAME-catalyzed H3pT11 represses the transcription of autophagy genes by inhibiting Dot1-catalyzed H3K79me3 (Fig. 4l).

Autophagy can be induced by glucose starvation^34^. When cells were grown under glucose starvation (SD-C) conditions, the occupancy of Pyk1 and H3pT11 at *ATG* genes was significantly reduced, while the occupancy of Dot1 and H3K79me3 at *ATG* genes was significantly increased (Supplementary Fig. 6a). The autophagy activity was significantly increased in H3T11A mutant but reduced in H3K79A mutant under glucose starvation conditions (Supplementary Fig. 6b). Mutation of H3K79A abolished the increased autophagy activity in H3T11A mutant (Supplementary Fig. 6b). Similarly, deletion of *DOT1* in *pyk1-ts* mutant abolished the increased autophagy activity in *pyk1-ts* mutant (Supplementary Fig. 6c). Consistent with the changes of autophagy activity when cells were grown in SD-C medium, mutation of H3K79A reduced the increased transcription of *ATG* genes in H3T11A mutant and loss of Dot1 reduced the increased transcription of *ATG* genes in *pyk1-ts* mutant under glucose starvation conditions (Supplementary Fig. 6d, e). These data indicate that under glucose starvation conditions, loss of H3pT11 leads to increased H3K79me3, which then activates the transcription of *ATG* genes to stimulate autophagy.

We also examined the effect of H3pT11 and H3K79me3 in autophagy activity when cells were grown in nitrogen starvation (SD-N) medium. Nitrogen starvation significantly increased autophagy in WT and mutation of H3T11A further increased autophagy; however, mutation of H3K79A significantly reduced the increased autophagy in H3T11A mutant (Supplementary Fig. 6f), suggesting that SESAME-catalyzed H3pT11 also represses autophagy by inhibiting Dot1-catalyzed H3K79me3 upon nitrogen starvation.

### H3pT11 and H3K79me3 work together to maintain telomere silencing

**Fig. 5:**
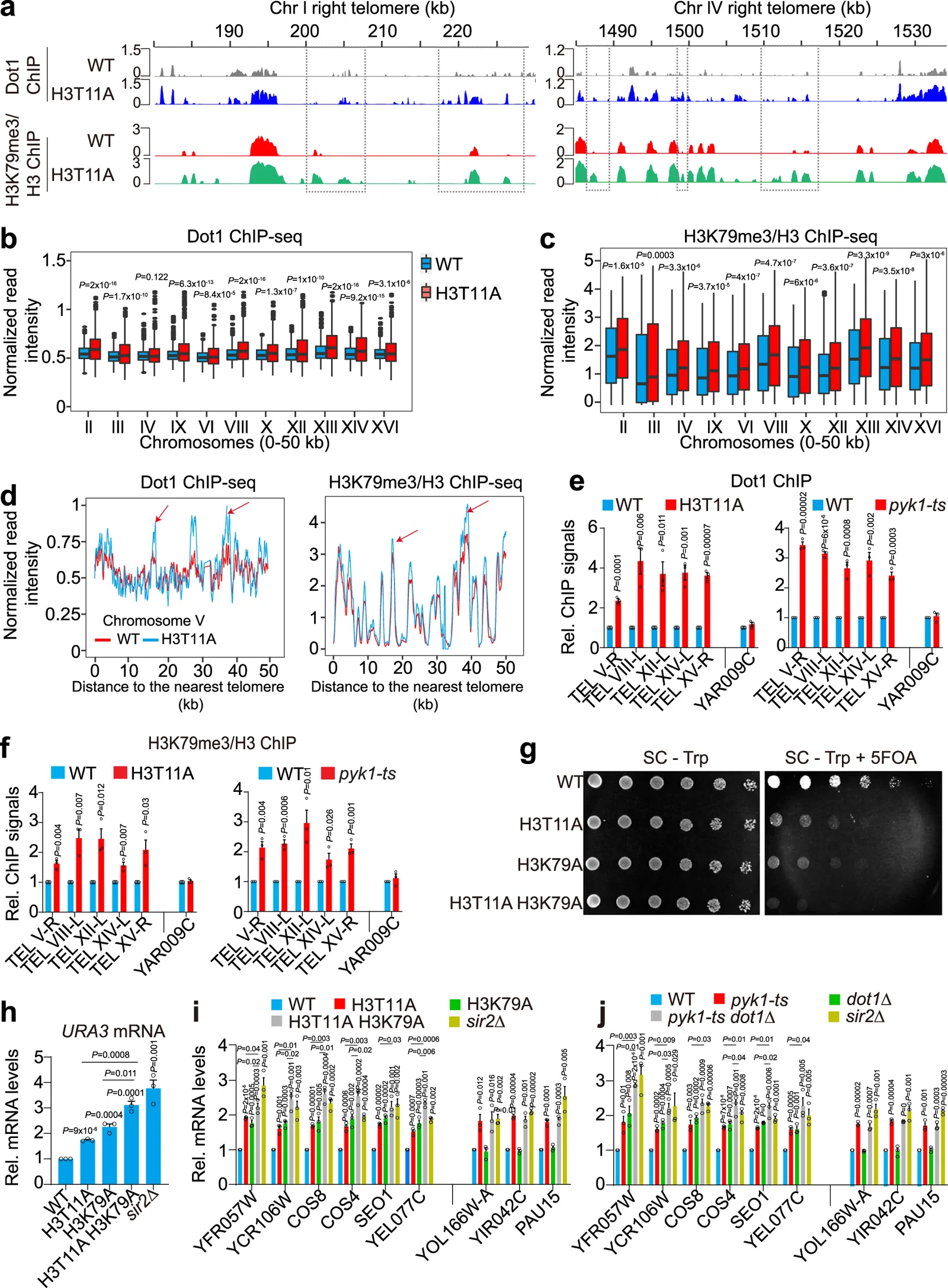
SESAME-catalyzed H3pT11 and Dot1-catalyzed H3K79me3 collaborate to promote telomere silencing. **a** ChIP-seq tracks showing the enrichment of Dot1 and H3K79me3/H3 at representative subtelomeric regions in WT and H3T11A mutant. **b**, **c** Box plots showing the normalized reads intensity of Dot1 (**b**) and H3K79me3/H3 (**c**) at subtelomeric regions in WT and H3T11A mutant. **d** The enrichment of Dot1 and H3K79me3/H3 at chromosome V was increased in H3T11A mutant. **e** ChIP-qPCR analysis of Dot1 occupancy at subtelomeric regions in WT, H3T11A, and *pyk1-ts* mutants. **f** ChIP-qPCR analysis of the enrichment of H3K79me3/H3 at subtelomeric regions in WT, H3T11A, and *pyk1-ts* mutants. **g** H3pT11 and H3K79me3 work together to promote telomere silencing. The telomere silencing reporter cells (WT, H3T11A, H3K79A and H3T11A H3K79A) bearing *URA3* adjacent to Tel VII-L were normalized for OD_600_, threefold serially diluted and spotted on SC - Trp with or without 5-FOA plates. The slower growth on 5-FOA plates indicates derepressed silencing of *URA3*. **h** RT-qPCR analysis of the transcription of *URA3* in WT, H3T11A, H3K79A, and H3T11A H3K79A mutants. **i** RT-qPCR analysis of the transcription of telomere-proximal genes in WT, H3T11A, H3K79A, H3T11A H3K79A, and *sir2Δ* mutants. **j** RT-qPCR analysis of the transcription of telomere-proximal genes in WT, *pyk1-ts*, *dot1Δ*, *pyk1-ts dot1Δ*, and *sir2Δ* mutants. For **e**, **f**, **h**–**j**, data represent the mean ± SE; *n* = 3 biological independent experiments. Two-sided *t*-tests were used for statistical analysis. For **g**, a typical example of three biologically independent replicates is shown. For **b**, **c**, centre lines denote medians, box limits denote 25th and 75th percentiles and whiskers denote maximum and minimum values. Two-sided Wilcoxon test in R (package ggpval) was used for statistical analysis. Source data are provided with this paper.

SESAME-catalyzed H3pT11 and Dot1-catalyzed H3K79me3 have been shown to regulate telomere silencing^14,23,35^. Our ChIP-seq data showed that the occupancy of Dot1 and H3K79me3 was significantly increased at subtelomere regions in H3T11A mutant (Fig. 5a–d; Supplementary Fig. 7a, b). Notably, in H3T11A mutant, there are some new peaks for H3K79me3 at subtelomere regions (Fig. 5a). In contrast, the occupancy of Dot1 and H3K79me3 was significantly reduced at subtelomere regions in H3T11D mutant (Supplementary Fig. 7c, d). ChIP-qPCR confirmed the increased occupancy of Dot1 and H3K79me3 at subtelomere regions in H3T11A and *pyk1-ts* mutants (Fig. 5e, f; Supplementary Fig. 7e), indicating that SESAME-catalyzed H3pT11 antagonizes Dot1-catalyzed H3K79me3 at subtelomere regions.

We thus investigated the effect of H3pT11-H3K79me3 crosstalk on telomere silencing. The H3T11A, H3K79A, and H3T11A H3K79A mutations were individually introduced into a telomere silencing reporter strain, which has the *URA3* reporter gene inserted adjacent to the left telomere of chromosome VII (Tel VII-L)^36^. Cells with repressed *URA3* were indicated by their ability to growth on medium containing 5-fluoroorotic acid (5-FOA), which can be converted into toxic 5-fluorouracil (5FU) by Ura3. Interestingly, although H3pT11 antagonizes H3K79me3, the growth of both H3T11A and H3K79A mutants on 5-FOA plate was reduced when compared to their WT counterpart (Fig. 5g). Strikingly, the growth of H3T11A H3K79A double mutant on 5-FOA plate was severely reduced even when compared with H3T11A and H3K79A mutants (Fig. 5g). The reduced growth of these mutants on 5-FOA plates was caused by increased transcription of *URA3* as determined by RT-qPCR (Fig. 5h), indicating that H3pT11 and H3K79me3 work together to maintain telomere silencing. In addition, we investigated the transcription of genes near the other telomeres, including *YFR057W*, *YCR106W*, *COS8*, *COS4*, *SEO1,* and *YEL077C*, which are located 2 kb from Tel VI-R, 3.2 kb from Tel III-R, 6.2 kb from Tel VIII-L, 6.2 kb from Tel VI-L, 7.2 kb from Tel I-L, and 0.5 kb from Tel V-L, respectively^23^. Similar to *URA3* derepression, the transcription of these telomere-proximal genes was significantly increased in H3T11A and H3K79A mutants and increased to a higher level in H3T11A H3K79A double mutant (Fig. 5i). Similar transcriptional derepression of these genes was observed in *pyk1-ts*, *dot1Δ* and *pyk1-ts dot1Δ* mutants (Fig. 5j), indicating that SESAME-catalyzed H3pT11 and Dot1-catalyzed H3K79me3 collaborate to promote telomere silencing.

### H3pT11 and H3K79me3 cooperate to enhance SIR complex binding at telomeres

**Fig. 6:**
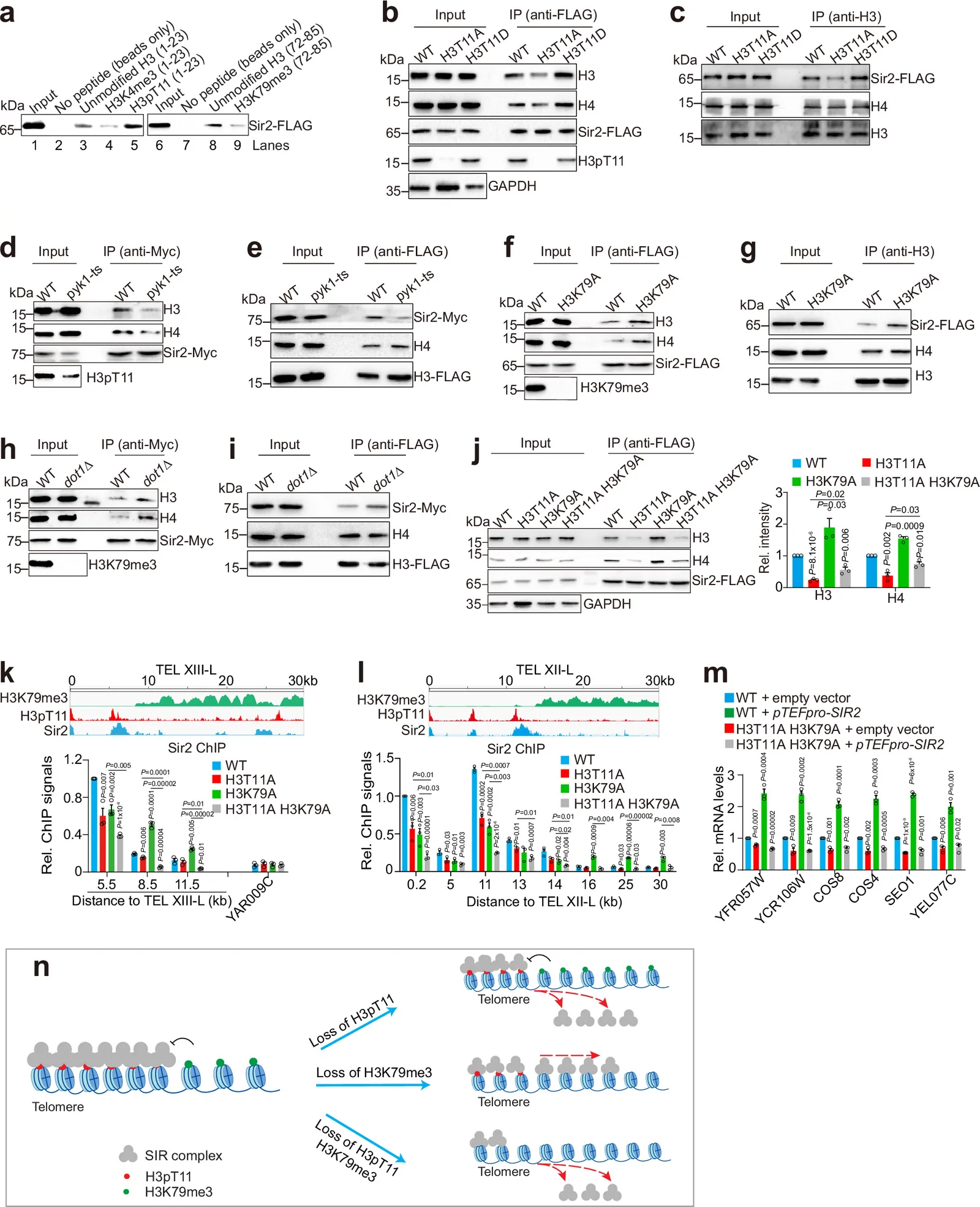
SESAME-catalyzed H3pT11 and Dot1-catalyzed H3K79me3 cooperate to promote SIR binding at telomere regions. **a** Peptide pull-down assays showing the relative binding ability of SIR complex to unmodified H3 peptides, H3K4me3 peptides, H3pT11 peptides, and H3K79me3 peptides. **b** In vivo endogenous Co-IP assays showing SESAME-catalyzed H3pT11 facilitates SIR complex binding to chromatin. Endogenously expressed Sir2-FLAG was immunoprecipitated from WT, H3T11A, and H3T11D mutants with anti-FLAG beads. To ensure equal loading of Sir2, more cell lysate of H3T11A mutant was used for immunoprecipitation. **c** Reciprocal IP assays showing SESAME-catalyzed H3pT11 facilitates SIR complex binding at chromatin. The nucleosomes were immunoprecipitated from cells with anti-H3 antibody. **d** In vivo endogenous Co-IP showing Pyk1-catalyzed H3pT11 promotes Sir2 binding to nucleosomes. Endogenously expressed Sir2-Myc was immunoprecipitated from WT and *pyk1-ts* mutant with anti-Myc beads. **e** The reciprocal IP showing Pyk1-catalyzed H3pT11 promotes Sir2 binding to nucleosomes. Endogenously expressed H3-FLAG-containing nucleosomes were immunoprecipitated from WT and *pyk1-ts* mutant with anti-FLAG beads. **f**, **g** In vivo endogenous Co-IP (**f**) and reciprocal IP (**g**) showing Dot1-catalyzed H3K79me3 prevents SIR complex binding to chromatin. **h**, **i** In vivo endogenous Co-IP (**h**) and reciprocal IP (**i**) showing Dot1-catalyzed H3K79me3 prevents SIR complex binding to chromatin. **j** In vivo endogenous Co-IP assays showing the relative binding of SIR complex to nucleosomes in WT, H3T11A, H3K79A, and H3T11A H3K79A mutants. **k** ChIP-qPCR analysis of Sir2 occupancy at regions with different distance to telomere XIII-L in WT, H3T11A, H3K79A, and H3T11A H3K79A mutants. Top panel, ChIP-seq tracks showing the distribution of H3pT11/H3, H3K79me3/H3, and Sir2 at telomere XIII-L. **l** ChIP-qPCR analysis of Sir2 occupancy at regions with different distance to telomere XII-L in WT, H3T11A, H3K79A, and H3T11A H3K79A mutants. **m**, RT-qPCR analysis of telomere-proximal gene transcription in WT and H3T11A H3K79A mutant transformed with empty vector or vector that overexpresses Sir2 (*pTEFpro-SIR2*). **n** Proposed model for how SESAME-catalyzed H3pT11 and Dot1-catalyzed H3K79me3 regulate telomere silencing. For **j**–**m**, data represent the mean ± SE; *n* = 3 biological replicates. Two-sided *t*-tests were used for statistical analysis. For **a**–**i**, a typical example of at least two biologically independent replicates was shown. Source data are provided with this paper.

To understand how SESAME-catalyzed H3pT11 and Dot1-catalyzed H3K79me3 function in parallel ways to promote telomere silencing, we examined their effects on SIR complex binding at chromatin. We performed the peptide pull-down assay by incubating purified endogenous SIR complex (Sir2-FLAG) with biotinylated unmodified, H3T11 phosphorylated, or H3K79 trimethylated H3 tail peptides. The SIR complex showed a binding preference for H3pT11 peptide (Fig. 6a, lane 3 vs lane 5) but had reduced binding to H3K79me3 peptide (Fig. 6a, lane 8 vs lane 9). We then performed in vivo endogenous Co-IP assay to examine the interaction between SIR complex with nucleosomes. We constructed WT, H3T11A, and H3T11D strains that express a Sir2-FLAG fusion protein from the endogenous *SIR2* locus, named Sir2-FLAG WT H3, Sir2-FLAG H3T11A, and Sir2-FLAG H3T11D. The endogenous Sir2-FLAG was immunoprecipitated (IPed) by anti-FLAG agarose beads from cell extracts. The bound nucleosomes were detected by immunoblots with anti-H3 and anti-H4 antibodies. The results showed that the Co-IPed nucleosomes were reduced in H3T11A mutant and slightly increased in H3T11D mutant (Fig. 6b), which is also confirmed by reciprocal IP (Fig. 6c). We also immunoprecipitated the endogenous Sir3-FLAG from cell lysates of Sir3-FLAG WT H3, Sir3-FLAG H3T11A, and Sir3-FLAG H3T11D. This experiment revealed that the Co-IPed nucleosomes were still reduced in H3T11A mutant and slightly increased in H3T11D mutant (Supplementary Fig. 8a). Similar reduced interaction between SIR complex and nucleosomes was also observed in *pyk1-ts* mutant (Fig. 6d, e; Supplementary Fig. 8b). Sir3 has been reported to bind at non-subtelomeric regions^36–39^, and we also observed the co-localization of H3pT11 with Sir3 at some non-subtelomeric regions (Supplementary Fig. 8c). Moreover, ChIP-qPCR analysis revealed that Sir3 occupancy at these loci was significantly reduced in H3T11A mutant (Supplementary Fig. 8c). These data indicate that SESAME-catalyzed H3pT11 promotes the binding of SIR complex to chromatin.

We also performed similar Co-IP and reciprocal IP in H3K79A and *dot1Δ* mutants. In contrary to H3T11A and *pyk1-ts* mutants, loss of H3K79me3 in H3K79A and *dot1Δ* mutants increased Sir2 binding to nucleosomes (Fig. 6f–i), indicating that Dot1-catalyzed H3K79me3 inhibits the binding of SIR complex to chromatin. To examine whether the reduced Sir2 binding to H3T11A was caused by increased H3K79me3, we performed Co-IP in H3T11A H3K79A double mutant. Although the SIR complex showed reduced binding to both H3T11A mutant and H3T11A H3K79A double mutant when compared with WT H3, the SIR complex bound slightly but significantly more H3T11A H3K79A nucleosomes than H3T11A nucleosomes (Fig. 6j), suggesting that on one hand, H3pT11 directly promotes the binding of SIR complex to nucleosomes; on the other hand, H3pT11 indirectly facilitates the binding of SIR complex by inhibiting H3K79me3.

We then examined the effect of these two modifications on SIR complex binding at telomere regions. H3pT11 primarily localizes close (~5 kb, telomere-proximal) to the left telomere of chromosome XIII (TEL XIII-L) and H3K79me3 primarily localizes distal (>~8 kb, centromere-proximal) from TEL XIII-L (Fig. 6k). Loss of H3pT11 led to reduced Sir2 occupancy across telomere regions; however, loss of H3K79me3 led to reduced Sir2 occupancy at regions from 0 to 5.5 kb but increased Sir2 occupancy at regions >8.5 kb (Fig. 6k), suggesting that SIR complex is redistributed in H3K79A mutant, which is consistent with the reported role of H3K79me3 as an euchromatin-heterochromatin boundary^14^. Strikingly, Sir2 occupancy was significantly reduced in H3T11A H3K79A double mutant compared with H3T11A and H3K79A mutants (Fig. 6k). Similar results were observed in left telomere of chromosome XII (TEL XII-L) (Fig. 6l).

To further confirm that H3pT11 and H3K79me3 synergistically regulate Sir2 occupancy and telomere silencing, we examined whether overexpression of Sir2 can rescue the telomere silencing defects in H3T11A H3K79A mutant. We transformed WT and H3T11A H3K79A mutant with the construct that overexpresses *SIR2* under the constitutive strong *TEF1* promoter (pTEFpro-*SIR2*). The transcription of telomere-proximal genes was significantly increased in H3T11A H3K79A mutant; however, overexpression of *SIR2* abrogated the increased transcription of these genes in H3T11A mutant (Fig. 6m), indicating that the telomere silencing defects in H3T11A H3K79A mutant can be rescued by *SIR2* overexpression.

Considering the differential effect of H3pT11 and H3K79me3 on SIR binding as well as their different locations at chromatin, we proposed that H3pT11 and H3K79me3 act cooperatively to promote the binding of SIR at telomere regions to maintain telomere silencing (Fig. 6n). In the absence of H3pT11, SIR complex dissociates from telomeres and more H3K79me3 occurred in telomere-proximal regions. In the absence of H3K79me3, SIR complex spreads from telomere-proximal regions to telomere-distal regions, leading to decreased SIR binding at telomere-proximal regions. In the absence of both H3pT11 and H3K79me3, more SIR complex dissociates from telomeres.

### Reb1 recruits SESAME complex to phosphorylate H3T11 and inhibit H3K79me3 at subtelomere regions

**Fig. 7:**
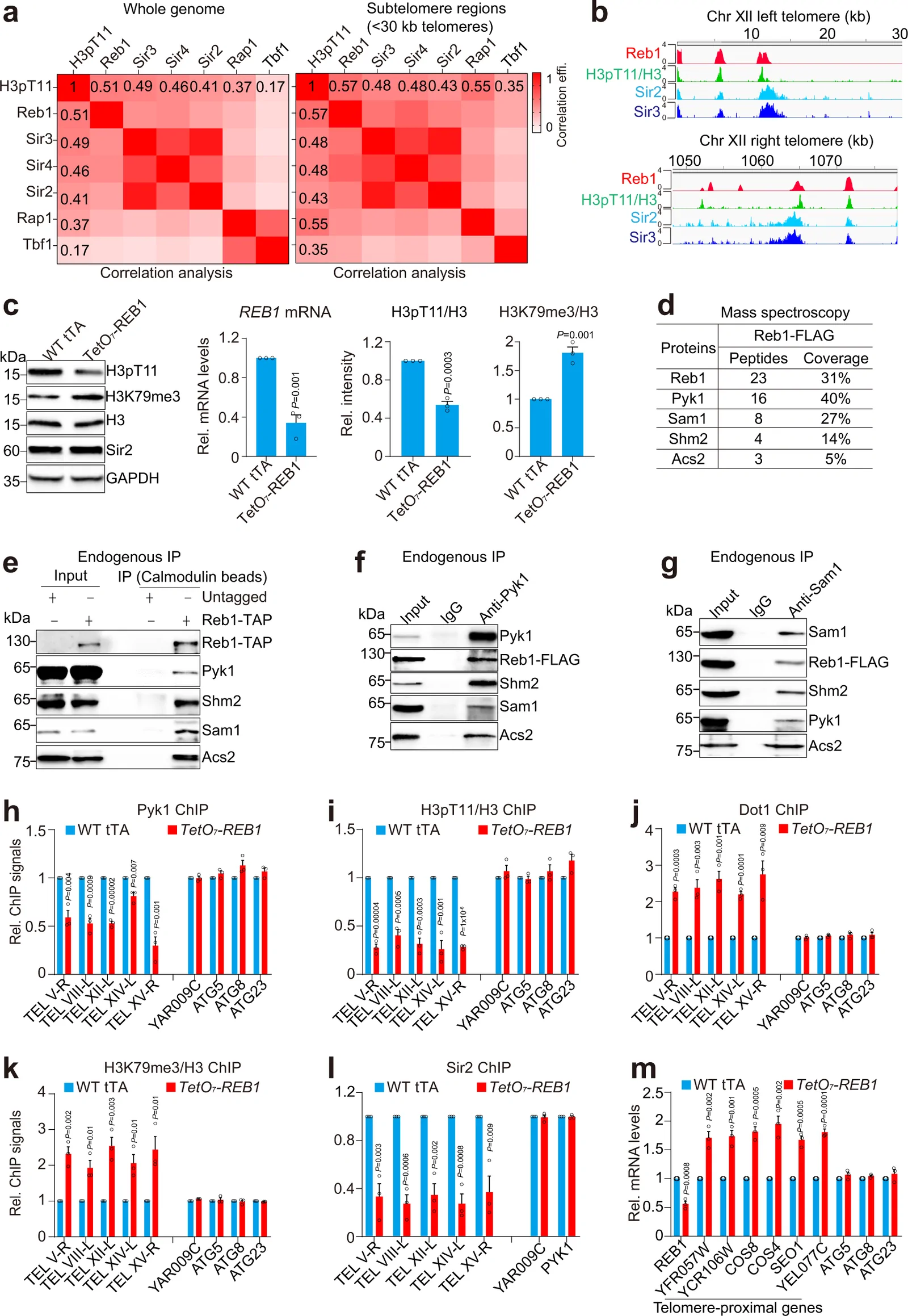
Reb1 recruits SESAME to phosphorylate H3T11 and inhibit Dot1 to catalyze H3K79me3 at telomere regions. **a** Correlation analyses of ChIP-seq data of H3pT11 with telomere-associated proteins at the whole genome (left panel) and subtelomeric regions (<30 kb from the nearest telomeres, right panel). **b** ChIP-seq tracks showing the enrichment of Reb1, H3pT11/H3 and Sir2 at representative subtelomeric regions. **c** Knockdown of *REB1* led to reduced H3pT11 and increased H3K79me3. WT tTA, and TetO_7_-*REB1* mutant were grown in YPD medium to an OD_600_ of 0.5 and then treated with 120 µg/ml doxycycline for 3 h. **d** LC-MS analysis of proteins interacting with Reb1. **e** In vivo endogenous Co-IP assays showing SESAME complex interacted with Reb1. Reb1-TAP was immunoprecipitated with calmodulin beads. **f**, **g** The reciprocal endogenous Co-IP assays showing SESAME complex interacted with Reb1. Pyk1 and Sam1 were immunoprecipitated with anti-Pyk1 and anti-Sam1 antibodies, respectively. **h–l** ChIP-qPCR analysis of the enrichment of Pyk1, H3pT11/H3, Dot1, H3K79me3/H3 and Sir2 at telomere-proximal regions in WT tTA and *TetO*_*7*_*-REB1*. Reb1 had no effect on *ATG* genes. *YAR009C* was used as a negative control. **m** RT-qPCR analysis of the transcription of telomere-proximal genes in WT tTA and *TetO*_*7*_*-REB1*. For **c**, **h**–**m**, data represent the mean ± SE; *n* = 3 biological independent experiments. Two-sided *t*-tests were used for statistical analysis. For **e**–**g**, a typical example of three biologically independent replicates was shown. Source data are provided with this paper.

To understand how SESAME is recruited to phosphorylate H3T11 at telomeres, we aimed to identify factor(s) that regulates H3pT11 at telomeres. Although Set1 is required to recruit SESAME to phosphorylate H3T11 at active genes and *ATG* genes^33^ (Supplementary Fig. 5l), Set1 had no significant effect on the binding of Pyk1 and Dot1 at telomeres (Supplementary Fig. 9a, b), consistent with our reports that the binding of SESAME at telomeres is independent of Set1-catalyzed H3K4me3^23^. By comparing the ChIP-seq data of H3pT11 to those of known telomere regulating proteins^40–43^, we found that the genome-wide occupancy pattern of H3pT11 was similar to Reb1 with a correlation co-efficient of 0.52 (Supplementary Fig. 9c). We also performed ChIP-seq for Reb1 and found that Reb1 had a similar occupancy pattern as H3pT11 with correlation co-efficient of 0.51 and 0.57 for the whole genome and subtelomeric regions, respectively (Fig. 7a, b).

Reb1 is a telomere-associated factor that regulates telomere silencing and telomere elongation^44–47^. We first examined H3pT11 in *TetO*_*7*_*-REB1* mutant, where the *REB1* promoter was replaced with TetO_7_ and *REB1* transcription can be shut off by doxycycline treatment^26^ (Fig. 7c). Knockdown of *REB1* significantly reduced H3pT11 (Fig. 7c), indicating that Reb1 promotes SESAME-catalyzed H3T11 phosphorylation. We also found that H3K79me3 was significantly increased in *TetO*_*7*_*-REB1* mutant (Fig. 7c).

We then investigated whether Reb1 interacts with SESAME complex. Reb1-FLAG was immunoprecipitated by anti-FLAG beads and liquid chromatography-tandem mass spectrometry (LC-MS) analysis revealed the presence of SESAME subunits, Pyk1, Shm2, Sam1, and Acs2 (Fig. 7d). We also confirmed their interaction by Co-IP assay. When the endogenous Reb1 was immunoprecipitated from cell extract containing tandem affinity purification (TAP)-tagged Reb1 (Reb1-TAP), SESAME subunits, Pyk1, Shm2, Sam1 and Acs2 were co-IPed with Reb1 (Fig. 7e). We also performed IP with anti-Pyk1 and found that SESAME subunits, Shm2, Sam1, Acs2 were co-IPed with endogenous Pyk1 (Fig. 7f). Moreover, the endogenous Reb1 was co-IPed with Pyk1 (Fig. 7f). Further Co-IP experiments with anti-Sam1 also revealed the interaction between endogenously expressed Reb1 and SESAME complex (Fig. 7g). In contrast, Dot1 had no interaction with Reb1 (Supplementary Fig. 9d).

We then performed ChIP-qPCR to examine the effect of Reb1 on Pyk1 binding at telomeres. Knockdown of *REB1* significantly reduced the occupancy of Pyk1 and H3pT11 at telomeres (Fig. 7h, i). Consistently, an increased enrichment of Dot1 and H3K79me3 at telomeres was observed in *TetO*_*7*_*-REB1* mutant (Fig. 7j, k). In accordance with the changes of H3pT11 and H3K79me3, we observed significantly reduced Sir2 binding at telomeres in *TetO*_*7*_*-REB1* mutant (Fig. 7l). Reb1 had no interaction with Sir2, Sir3, and Sir4 as determined by Co-IP assay (Supplementary Fig. 9e, f), suggesting that Reb1 does not directly recruit SIR complex to telomeres via Reb1-SIR interaction but indirectly via the H3pT11-H3K79me3 crosstalk. As a consequence of reduced Sir2 occupancy at telomere regions, the transcription of telomere-proximal genes was significantly increased in *TetO*_*7*_*-REB1* mutant (Fig. 7m). These data demonstrate that Reb1 recruits SESAME complex to phosphorylate H3T11, which prevents the promiscuous binding of Dot1 at telomere heterochromatin regions, leading to enhanced SIR complex binding at telomeres. Thus, we identified the Reb1-SESAME-Dot1 axis to establish telomere heterochromatin-euchromatin boundary.

Reb1 has also been reported to block the spread of silencing^46,47^. We thus re-examined the ChIP-seq data for Reb1, H3pT11 and SIR complex at subtelomere regions. Reb1, H3pT11 and SIR complex co-localized at 19 of 32 analyzed subtelomeric regions (Supplementary Fig. 10a). Reb1 occupied at 12 of the 32 analyzed subtelomeric regions where the H3pT11 enrichment was low (Supplementary Fig. 10b). H3T11 phosphorylation was enriched at one subtelomeric region with little occupancy of Reb1 (Supplementary Fig. 10c). We hypothesized that the role of Reb1 may vary among different telomeres. Reb1 may block the spreading of SIR complex at telomere-proximal regions where H3pT11 is low, while Reb1 may recruit SIR complex at telomere-proximal regions where Reb1, H3pT11 and SIR complex co-localize. We examined the effect of Reb1 on Sir2 binding at telomere-proximal regions where H3pT11 is low, such as XIII-R. Our data showed that Sir2 occupancy was significantly reduced in telomere-proximal regions but increased in telomere-distal regions in *TetO*_*7*_*-REB1* mutant (Supplementary Fig. 11a). Similar results were also observed in Tel II-R, Tel VII-L, Tel IX-R, Tel XI-L, and Tel XI-R (Supplementary Fig. 11b). As a result of increased Sir2 binding at these regions, the transcription of genes near these regions was significantly reduced (Supplementary Fig. 11c), suggesting that Reb1 could block the spread of SIR complex at these regions. We then examined the effect of Reb1 on Sir2 binding at telomere-proximal regions where Reb1, H3pT11 and SIR complex co-localized, such as XII-L. Knockdown of Reb1 significantly reduced the occupancy of Sir2 at XII-L and no significant increased Sir2 occupancy was observed in telomere-distal regions (Supplementary Fig. 11d). Similar results were observed in Tel II-L, Tel VII-R, Tel VIII-L, Tel VIII-R, Tel XII-L, and Tel XII-R (Supplementary Fig. 11e). As a consequence, the transcription of gene near these regions was significantly increased in *TetO*_*7*_*-REB1* mutant (Supplementary Fig. 11f). Thus, the Reb1 promotes the binding of SIR complex at some but not all telomere regions via the SESAME-Dot1 axis.

### H3pT11 and H3K79me3 regulate telomere silencing during glucose starvation conditions

**Fig. 8:**
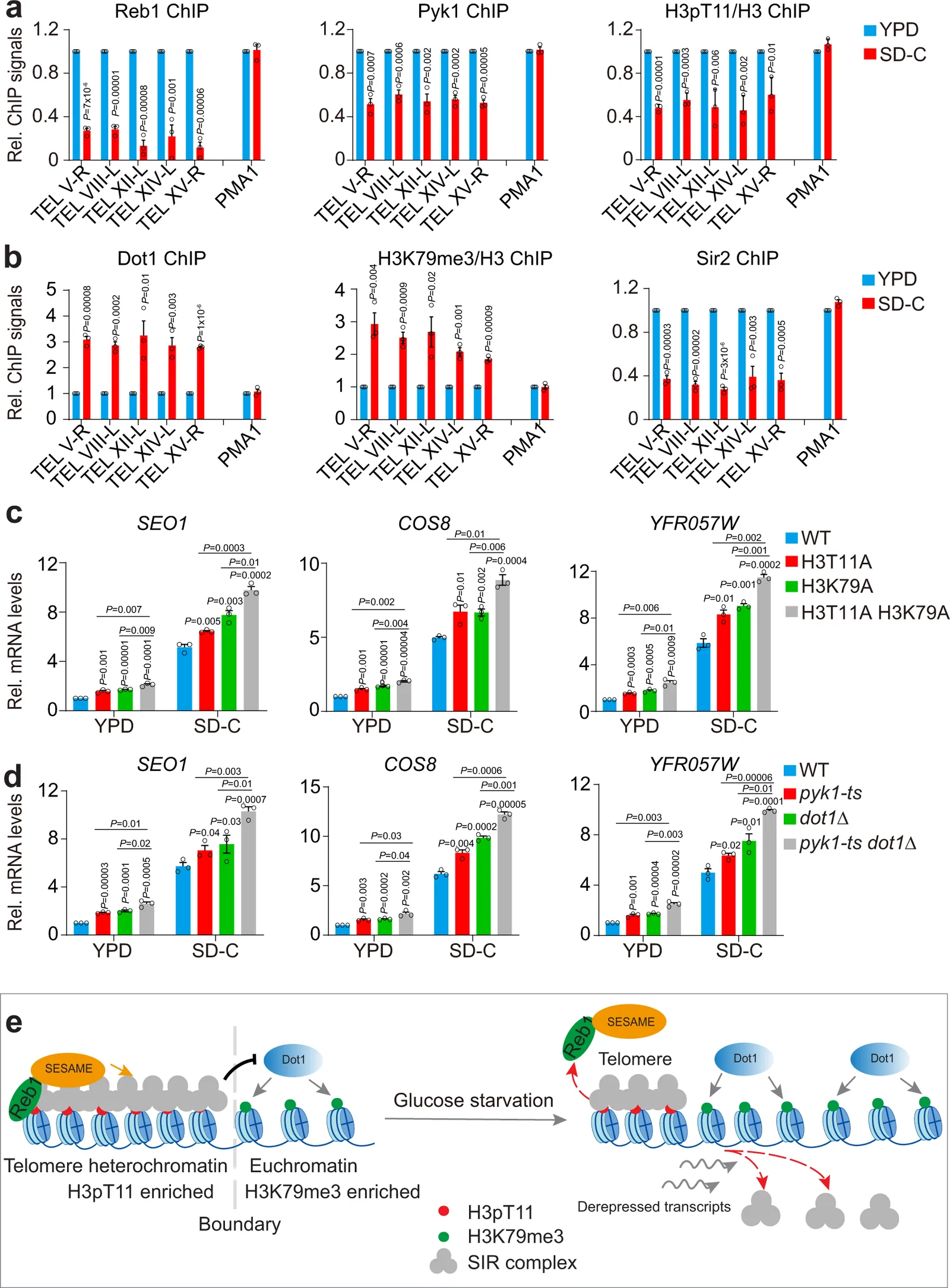
H3pT11 and H3K79me3 work together to regulate telomere silencing during glucose starvation conditions. **a** ChIP-qPCR analysis of the enrichment of Reb1, Pyk1 and H3pT11/H3 at telomere-proximal regions when cells were grown in YPD and SD-C medium, respectively. Yeast cells were grown in YPD until OD_600_ of 1.0. Cells were harvested, resuspended in SD-C and then grown for 3 h. **b** ChIP-qPCR analysis of the enrichment of Dot1, H3K79me3/H3, and Sir2 at telomere-proximal regions when cells were grown in YPD and SD-C medium, respectively. **c** RT-qPCR analysis of the transcription of telomere-proximal genes (*SEO1*, *COS8*, *YFR057W*) in WT, H3T11A, H3K79A, and H3T11A H3K79A mutants when cells were grown in YPD and SD-C medium, respectively. **d** RT-qPCR analysis of the transcription of telomere-proximal genes (*SEO1*, *COS8*, *YFR057W*) in WT, *pyk1-ts*, *dot1Δ,* and *pyk1-ts dot1Δ* mutants when cells were grown in YPD and SD-C medium at 39 °C for 2 h, respectively. **e** Proposed model for regulation of telomere silencing by the Reb1-SESAME-Dot1 pathway. For **a**–**d**, data represent the mean ± SE; *n* = 3 biological independent experiments. Two-sided *t*-tests were used for statistical analysis. Source data are provided with this paper.

As it has been established that H3pT11 was significantly reduced and H3K79me3 was significantly increased when cells were grown under glucose starvation conditions (Fig. 1f), we hence examined the role of Reb1-SESAME-Dot1 axis in telomere silencing under glucose starvation conditions. When cells were grown in glucose-depletion medium (SD-C), although the protein level of Reb1 remained unchanged (Supplementary Fig. 12a), the occupancy of Reb1 at telomere regions was significantly reduced (Fig. 8a). The enrichment of Pyk1 and H3pT11 at telomere regions was significantly reduced, whereas the occupancy of Dot1 and H3K79me3 was significantly increased at telomere regions, leading to reduced Sir2 occupancy at telomere regions (Fig. 8a, b). When cells were grown in SD-C, the interaction between Reb1 and Pyk1 was not reduced but slightly increased (Supplementary Fig. 12b), suggesting that the reduced occupancy of Pyk1 at telomere-proximal genes was caused by reduced Reb1 binding. As a result, the transcription of telomere-proximal genes was significantly increased when cells were grown in SD-C (Fig. 8c).

The transcription of telomere-proximal genes was significantly increased in H3T11A and H3K79A mutant when cells were grown in SD-C (Fig. 8c). Simultaneous mutation of H3T11A and H3K79A further increased the transcription of telomere-proximity genes (Fig. 8c). Similar results were observed in *pyk1-ts dot1Δ* mutant under glucose starvation conditions (Fig. 8d). Collectively, these data support a model that under normal conditions, Reb1 recruits SESAME to telomere regions to phosphorylate H3T11 and inhibit Dot1-catalyzed H3K79me3, resulting in formation of telomere-proximal H3pT11 and centromere-proximal H3K79me3. This specific distribution pattern of H3pT11 and H3K79me3 promotes SIR complex binding at telomere regions and enhances telomere silencing. During glucose starvation, Reb1 is dissociated from telomeres, which reduces the occupancy of SESAME and H3pT11 and increases the occupancy of Dot1 and H3K79me3 at subtelomere regions, leading to decreased SIR complex binding and compromised telomere silencing (Fig. 8e).

## Discussion

Pyruvate kinase Pyk1-containing SESAME complex phosphorylates histone H3T11 to maintain telomere silencing by promoting SIR complex binding at telomeres and preventing autophagy-mediated Sir2 degradation, which provides a direct connection between cell metabolism, autophagy, and telomere silencing^23^. However, the exact mechanism of action for H3pT11 is poorly understood. Here, we find that Pyk1-catalyzed H3pT11 antagonizes Dot1-catalyzed H3K79me3 to exert its biological effects. Specifically, SESAME-catalyzed H3pT11 directly prevents the binding of Dot1 to nucleosomes and inhibits its activity to tri-methylate H3K79. Dot1-catalyzed H3K79me3 transcriptionally activates autophagy and SESAME-catalyzed H3pT11 represses autophagy by inhibiting Dot1-catalyzed H3K79me3. Although SESAME-catalyzed H3pT11 inhibits Dot1-catalyzed H3K79me3, these two histone marks act together to maintain telomere silencing by promoting the binding of SIR complex at telomere regions. In addition, we identify Reb1 as a telomere-associated factor that recruits SESAME to telomeric regions to phosphorylate H3T11 and prevent the invasion of H3K79me3 into telomere heterochromatin, which constitutes the Reb1-SESAME-Dot1 axis. When cells are grown under glucose starvation, Reb1 is dissociated from telomeres, which reduced H3pT11 and increased H3K79me3 at telomeric regions, leading to compromised telomere silencing (Fig. 8e). Thus, our study identifies Dot1-catalyzed H3K79me3 as a downstream effector for SESAME-catalyzed H3pT11 and provides a mechanistic insight into epigenetic regulation of autophagy and telomere silencing.

H3K79 is methylated by Dot1 in yeast and DOT1L in mammals, among which DOT1L plays an important role in a subset of leukemias that express an MLL fusion protein caused by rearrangements of the MLL gene (MLL-r)^48^. Some DOT1L inhibitors have been developed and are in clinical trials for the treatment of MLL-r leukemia^49^. H3K79me3 regulators could become potential drug targets for diseases in which DOT1L has been implicated. Dot1-catalyzed H3K79me3 has been reported to be regulated by histone modifications. The well-known regulatory mechanism is the trans-histone crosstalk: Dot1-catalyzed H3K79me3 is dependent on Rad6/Bre1-catalyzed H2BK123 monoubiquitination (H2Bub)^29^, which is conserved in mammals, where ubiquitination of H2B by RNF20/40 promotes H3K79 methylation by DOT1L^48^. In addition, the activity of Dot1 is positively regulated by histone acetylation. The histone deacetylase Rpd3 reduces Dot1-catalyzed H3K79me3 and loss of Rpd3 or HDAC1 increases H3K79me3^24^. A more recent study showed that acetylation of H4K16 (H4K16ac) directly stimulates the catalytic activity of Dot1 on nucleosomes^30^. H4K16ac, together with H2Bub, allosterically enhance methylation of H3K79 by Dot1^30^. H3K79me3 is a very stable histone mark and can exist and accumulate on old histones^50^. To date, no H3K79 demethylase has been identified in yeast and how H3K79 methylation is negatively regulated remains a mystery. Here, we identify SESAME-catalyzed H3pT11 as a negative regulator of Dot1-catalyzed H3K79me3. The inhibitory effect of H3pT11 is direct, resulting in a reduced substrate binding and low enzymatic activity of Dot1. Therefore, H3pT11 is an important mechanism to counteract Dot1 activity in the absence of H3K79 demethylase. PKM2, the homolog of Pyk1 in mammals, has been reported to phosphorylate H3T11 and play critical role in Mixed-lineage leukemia (MLL) fusions-induced leukemia^51,52^. It is possible that the crosstalk between PKM2-catalyzed H3pT11 and DOT1L-catalyzed H3K79me3 plays a role in leukemia.

Autophagy is an evolutionarily conserved cellular process that primarily participates in lysosome-mediated protein degradation. Transcriptional regulation of *ATG* genes represents an important way to control autophagy^32^. Autophagy is regulated by histone modifications. Under starvation conditions, reduced H2B monoubiquitination (H2Bub) results in the activation of autophagy by controlling the transcription of autophagy regulatory genes^53^. The histone deacetylase Rpd3 represses the transcription of autophagy genes to inhibit autophagy^54^. The acetyltransferase hMOF-catalyzed H4K16ac is involved in autophagy regulation^55^. Shin et al. identified co-activator-associated arginine methyltransferase 1 (CARM1) as a crucial regulator of autophagy in mammals^56^. Increased histone H3R17 dimethylation by CARM1 is required for proper autophagy activity^56^. Although we have previously shown that H3pT11 inhibits autophagy by repressing the transcription of autophagy genes, it remains unclear about how H3pT11 represses the transcription of autophagy genes as no H3pT11 reader or effector has been characterized so far. Here, we identify Dot1-catalyzed H3K79me3 as a positive regulator of autophagy by facilitating the transcription of autophagy genes. By inhibiting Dot1-catalyzed H3K79me3, H3pT11 represses the transcription of autophagy genes and reduces autophagy. Since loss of Set2, which catalyzes H3K36 methylation, has no significant effect on autophagy, it is unlikely that H3pT11 represses the transcription of autophagy genes by inhibiting Set2-catalyzed H3K36 methylation. As most autophagy genes are responsible for autophagosome formation and down-regulation of these autophagy genes may lead to reduced autophagic activities, it remains to be determined whether there is a subset of autophagy genes that specifically accounts for H3pT11-mediated repression of autophagy.

It has been established that histone modifications, including Sas2-catalyzed H4K16ac^54^, Dot1-catalyzed H3K79me3^20^, Set1-catalyzed H3K4me3^21^, play important roles in regulation of telomere silencing by preventing the SIR complex from spreading from heterochromatin to euchromatin regions. However, it is unclear about the mechanism to prevent euchromatin invading into heterochromatin regions. Our previous work identified SESAME-catalyzed H3pT11 as a histone mark that is enriched in telomere heterochromatin regions and maintains Sir2 homeostasis^23^. Here, we show that SESAME-catalyzed H3pT11 promotes the binding of SIR complex to telomere regions, which is consistent with the reports that H3 N-termini 1-23 can be bound by Sir3 and Sir4^57^. Moreover, H3pT11 prevents the mislocalization of H3K79me3 at telomere heterochromatin, which facilitates the establishment of boundary between heterochromatin and euchromatin. Kitada and colleagues reported that the presence or absence of H3K79me3 is the critical determinant of whether the subtelomeric loci are active or silenced^3^. However, due to lack of H3K79 demethylase, H3K79me3 is not actively demethylated and therefore only diluted by rounds of cell division, which makes it difficult for selective exclusion of H3K79me3 from silent domains^58^. Here, we find an antagonistic relationship between H3pT11 and H3K79me3. The presence of H3pT11 inhibits the promiscuous occurrence of H3K79me3 at silent chromatin, which compensates for the absence of H3K79 demethylase. Moreover, H3pT11 enhances SIR complex binding at silent chromatin and H3K79me3 prevents SIR binding at active chromatin, which makes SIR complex highly concentrated and restricted in certain areas. As a consequence, H3pT11, inhibition of Dot1 activity and replicative dilution of H3K79me3 synergistically promote SIR complex binding at telomeres and enhance telomeric silencing. The binding of Dot1 to chromatin can positively affect local gene expression by inducing chromatin rearrangements^59^. Moreover, Dot1 has been reported to regulate nucleosome dynamics^60^. We cannot exclude the possibility that the combined effect of H3T11A and H3K79A on gene silencing could be due to a destabilization of nucleosomes by these mutations.

We also examined whether H3pT11 works with Set1-catalyzed H3K4me3 and Sas2-catalyzed H4K16ac to regulate telomere silencing. Although the transcription of telomere-proximal genes was significantly increased in H3T11A, *set1Δ*, and H3T11A *set1Δ* mutants, there was no significant difference between *set1Δ* and H3T11A *set1Δ* mutants and no additive effect was observed for H3T11A and *set1Δ* mutants (Supplementary Fig. 13a). The transcription of telomere-proximal genes was significantly reduced in *sas2Δ* mutant but increased in H3T11A and H3T11A *sas2Δ* mutants (Supplementary Fig. 13b). Thus, our data suggest that H3pT11 cooperates with Dot1-catalyzed H3K79me3 but not Set1-catalyzed H3K4me3 or Sas2-catalyzed H4K16ac to promote telomere silencing.

Takahashi et al. have reported that loss of Dot1 and H3K79 methylation affects the transcription of a few telomere-proximal genes^61^. We confirmed that loss of Dot1 and mutation of H3K79A did not affect the expression of subtelomeric genes reported in their work, including *YOL166W-A*, *YIR042C*, and *PAU15* (Fig. 5i, j). However, these genes were significantly increased in H3T11A, H3T11A H3K79A, *pyk1-ts*, and *pyk1-ts dot1Δ* mutants (Fig. 5i, j), suggesting that Pyk1-catalyzed H3pT11 may have a broad role in regulating telomere silencing. It is highly likely that H3pT11 does not act together with H3K79me3 at all subtelomeric regions. At some subtelomere regions, H3T11 phosphorylation promotes telomere silencing independent of H3K79me3. Meanwhile, Takahashi et al. proposed that other factors work together with Dot1-catalyzed H3K79me3 to regulate telomere silencing^61^, which may make loss of Dot1 alone have no marked effect on telomere silencing. We think H3pT11 could be the factor that acts with Dot1-catalyzed H3K79me3 to regulate telomere silencing.

The function of Pyk1 and H3pT11 at telomeric regions is independent of Set1-catalyzed H3K4me3. Instead, we identify Reb1 as a transcription factor that recruits SESAME complex to telomeric regions to phosphorylate H3T11. Reb1 is present in the subtelomeric regions to negatively regulate telomere length by inhibiting the activity of telomerase^45^. Our data showed that Reb1 has high similar binding pattern with H3pT11 at telomeric regions. Moreover, Reb1 directly interacts with SESAME but not Dot1. By promoting SESAME-catalyzed H3pT11, Reb1 prevents the invasion of H3K79me3 into telomere heterochromatin. Therefore, our work identifies an epigenetic mechanism for regulation of telomere silencing by Reb1. Reb1 has also been reported to block the spread of silencing^46,47^. Our results suggest that Reb1 could prevent the spread of SIR complex at telomeres where H3pT11 is low. At telomere regions where Reb1, H3pT11, and SIR co-localize, Reb1 could promote the binding of SIR complex at telomeres. Therefore, the role of Reb1 in SIR complex binding may vary among different telomeres.

Reb1 does not co-localize with H3pT11 and SIR complex at 13 telomeric regions. There are two possible reasons. Firstly, Reb1 is probably not the sole telomere-associated factor determining H3pT11 at telomeres. H3pT11 has a correlation coefficient of 0.55 with another telomere-binding protein Rap1 (Fig. 7a). It is possible that Rap1 could regulate H3pT11. Secondly, H3T11 could be dephosphorylated by other proteins at telomere regions. Further efforts are required to address this question.

Together, we identify a histone crosstalk between SESAME-catalyzed H3pT11 and Dot1-catalyzed H3K79me3. By directly inhibiting Dot1-catalyzed H3K79me3, we uncover how SESAME-catalyzed H3pT11 represses the transcription of autophagy genes and prevents autophagy. Our work also provides an example in which the activity of methyltransferase Dot1 is modulated through histone crosstalk to ensure optimal maintenance and propagation of the telomeric silent state.

## Methods

### Materials

All yeast strains used in this study are described in Supplementary Table 1. The promoter-shutoff mutants (WT tTA, TetO_7_-*ENO2*, TetO_7_-*PGI1*, TetO_7_-*REB1*) were purchased from Yeast Tet-promoters Hughes Collection in Open Biosystems (GE Dharmacon)^26^. The gene-deletion mutants and genomic integration of C-terminal epitope tags were constructed by homologous recombination of PCR fragments according to standard protocols^62^. All yeast strains were verified by colony PCR, DNA sequencing, qRT-PCR, and/or immunoblots before being used in experiments. The effect of epitope tags on protein functions was checked by growth assays and immunoblots for histone modifications. The primers used for qPCR are listed in Supplementary Table 2.

### Cell growth and treatment

For most experiments, yeast cells were grown in 2% glucose-containing YPD (Yeast Extract Peptone Dextrose) medium at 30 °C until OD_600_ of 0.7. For nitrogen starvation treatment, cells were grown in YPD until OD_600_ of 0.7 and then harvested at 2000 × *g* for 5 min. After being washed with sterile double-distilled water (ddH_2_O) twice, cells were grown in SD-N medium (0.17% yeast nitrogen base without amino acids and ammonium sulfate, 2% dextrose) for 0–1 h. For glucose starvation treatment, cells were grown in YPD until OD_600_ of 0.7 and then pelleted at 2000 × *g* for 5 min. After being washed with sterile ddH_2_O twice, cells were grown in SD-C medium (0.67% yeast nitrogen base without amino acids, supplemented with amino acids, no dextrose) for 0–6 h. For promoter-shutoff mutants (WT tTA, TetO_7_-*ENO2*, TetO_7_-*PGI1*, TetO_7_-*REB1*), cells were grown in YPD medium at 30 °C until OD_600_ of 0.5 and then treated with 40–120 μg/ml doxycycline for 3 h. The knockdown efficiency was determined by RT-qPCR (Supplementary Fig. 14). For *pyk1-ts* and *acs2-ts* mutants, cells were grown in 26 °C until OD_600_ of 0.5 and then transferred to pre-warmed medium at 39 °C for 2 h.

### Immunoblot analysis

Cells were grown in 5 ml YPD or selective medium as indicated until OD_600_ of 0.7–1.0 or conditions indicated. Cells were harvested by centrifugation at 2000 × *g* and lysed in alkaline lysis buffer (2 M NaOH, 8% (v/v) 2-mercaptoethanol) on ice for 15 min. After centrifugation at 15,000 × *g*, the protein pellet was resuspended in 100-150 μl 2×SDS-sample buffer. Protein samples were separated by 8–15% SDS-PAGE and transferred to Immobilon-P PVDF membrane (Bio-Rad). The blots were probed with primary antibodies followed by horseradish peroxidase-labeled IgG secondary antibodies. The protein bands were visualized using the ECL Chemiluminescence Detection Kit (Bio-Rad, 170-5061) and quantified with ImageJ.

### Antibodies

Antibodies against anti-H3 (1:5000; ab1791), anti-H4 (1:5000; ab10158) and H3pT11 (1:5000; ab5168) were purchased from Abcam; antibodies against H3K79me1 (1: 1000; A2367), H3K79me2 (1: 5000; A2368), H3K79me3 (1:5000; A2369), anti-His (1:5000; AE0039) and anti-FLAG (1:5000; AE024) were purchased from Abclonal; antibody against Sir2 (1:500; sc-6667) was purchased from Santa Cruz Biotechnology; antibodies against GAPDH (1:10,000; 10494-1-AP), GFP (1:5000; 66002-1-1 g), Myc (1:5000; 60003-2-1 g), goat polyclonal anti-mouse IgG (1:5000; SA00001-1) and goat polyclonal anti-rabbit IgG (1:5000; SA00001-2) were obtained from Proteintech; antibody against FLAG M2 (1:3000; F1804-1MG) was obtained from Sigma-Aldrich; antibody against H4K16ac (1:2000; 07-329) was obtained from EMD Millipore; antibody against CBP (1:2000; Abs130593) was purchased from Absin Bioscience Inc. The custom-made antibodies against Acs2, Pyk1, Sam1 and Shm2 were produced by Covance Inc. and their specificity was verified by immunoblots of cell lysates of the corresponding histone point mutants, gene deletion or knockdown mutants^31^.

### Chromatin immunoprecipitation (ChIP) assay

Yeast cells were grown in 200 ml YPD media at 30 °C until OD_600_ of 1.0–1.5. The samples were crosslinked with 1% formaldehyde and the crosslinking was quenched by adding 10 ml of 2.5 M glycine. After centrifugation, cells were washed with cold washing buffer (TBS + 1 mM PMSF), lysed with glass beads in FA-SDS lysis buffer (40 mM HEPES-KOH, pH7.5, 1 mM EDTA, pH 8.0, 0.1% SDS, 1% Triton X-100, 0.1% Na deoxycholate, 1 mM PMSF, 2 μg/ml leupeptin, 1 μg/ml pepstatin A, protease inhibitor cocktail). Chromatin was sonicated to an average size of ~500 bp and then subjected to immunoprecipitation with anti-FLAG M2 antibody (5 μl, F1804, Sigma), anti-H3 (2 μl; ab1791, Abcam), anti-H3pT11 (2 μl; ab5168, Abcam), anti-H3K79me3 (2 μl; A2369, Abclonal) pre-bound to Protein G Dynabeads (Invitrogen) at 4 °C overnight. The beads were then washed successively with FA lysis buffer, FA buffer + 1 M NaCl, FA buffer + 0.5 M NaCl, TEL buffer (10 mM Tris, pH 8.0, 1 mM EDTA, 0.25 M LiCl, 1% NP-40, 1% Na deoxycholate) and TE (10 mM Tris, pH 7.4, 1 mM EDTA). The eluted DNA/protein complexes were treated with 2 µl Proteinase K (10 µg/µl) to remove proteins at 55 °C for 1 h and then treated at 65 °C overnight. The RNA was removed by digestion with RNase (Solarbio, R8020), purified with ethanol precipitation and quantitated by qPCR with primers listed in Supplementary Table 2. For ChIP-qPCR analysis, the IP signals for H3pT11 and H3K79me3 were normalized to H3; the IP signals for Dot1 and Pyk1 were normalized to input. The relative ChIP signals were then normalized to wild-type or untagged cells.

ChIP-seq was performed with anti-H3K79me3, anti-H3, and anti-FLAG M2 antibodies^33^. The libraries were constructed and sequenced on an Illumina platform^33^. Reads were aligned to yeast genome sacCer3 from UCSC using bowtie2 version 2.1.0 with parameter -k 1. Telomere repeated sequences were excluded for further analysis. Data was read into R (3.1.0) for further analysis. Peaks were called using MACS2 (v.2.1.1, macs2 callpeak) with parameter -t -c -g 1.2e7 -n -B -q 0.01 -nomodel. Peaks annotation was performed on a website service (https://manticore.niehs.nih.gov/pavis2/). Tracks were smoothed by deepTools2 (v.2.0) and visualized by IGV software (v.2.0) with a reference genome of *S. cerevisiae* (sacCer3).

For correlation analyses of Fig. 1a and 7a, we used the multiBigwigSummary function in deeptools (version 3.5.1) software to compute the average scores for each of the files in every genomic region. We used the bed-file mode to compute the correlation scores for the ChIP-seq data of indicated histone modifications in all yeast genes (sacSer3 sgdGene). The heatmap was generated by plotCorrelation function in the same software package using the Pearson method, where the colors represent the correlation coefficients. For Fig. 1b, c, e, the computeMatrix function in the same software package was used to compute the coverage of the indicated ChIP-seq signals in all yeast genes followed by plotHeatmap function to draw the profile plot and heatmap plot. For Fig. 1d, the indicated ChIP-seq bigwig file was visualized by IGV software.

### Quantitative reverse transcription PCR (qRT-PCR)

Total RNA was isolated from yeast cells grown to an OD_600_ of 0.5–0.7 by standard phenol-chloroform extraction procedures. DNA was removed by digestion with DNase I (Takara catalog no. 2270 A) for 0.5 h, quantified by Nanodrop 2000 (Thermo scientific) and its integrity was assessed by agarose gel electrophoresis. Generally, 500 ng total RNA was used for reverse transcription PCR (RT-PCR) in a 10 μl reaction volume with Reverse Transcriptase Kit (M-MLV) (ZOMANBIO)^33^. qPCR was performed with iTaq™ Universal SYBR® Green Supermix (Bio-Rad, 1725121) on a real-time PCR machine (Bio-Rad). Primers used for qPCR are described in Supplementary Table 2. The quantity of relative transcription level was calculated by 2^(-ΔΔCt). The mRNA level of the gene of interest was normalized to that of beta-actin. The qRT-qPCR was performed and analyzed by following the MIQE guidelines^63^.

### Immunoprecipitation

Immunoprecipitation was performed as described with minor modifications^21,64^. The whole cell extract was prepared by vortexing with glass beads and then digested with Micrococcal Nuclease (MNase). For TAP-tagged strains, the whole cell extract was incubated with 30 μl of calmodulin beads (GE Healthcare) for 4 h at 4 °C in calmodulin binding buffer (10 mM Tris, pH 8.0, 150-350 mM NaCl, 1 mM MgOAc, 2 mM CaCl_2_, 0.1% NP-40, 10% glycerol, 1 mM PMSF, 2 μg/ml leupeptin, 1 μg/ml pepstatin A, protease inhibitor cocktail). For other IPs, the whole cell extract was mixed with 30 μl anti-Myc agarose beads (Proteintech), or anti-FLAG affinity gel (Gescript) and incubated for 1 hr to overnight at 4 °C in IP binding buffer (40 mM HEPES-KOH, pH7.5, 0.1% NP-40, 10% glycerol, 1 mM PMSF, 150–350 mM NaCl, 2 μg/ml leupeptin, 1 μg/ml pepstatin A, protease inhibitor cocktail). The beads were washed three times with 1 ml calmodulin binding buffer or IP binding buffer. The beads were boiled and the supernatant was subject to immunoblot analysis.

For in vitro immunoprecipitation, Dot1-FLAG was affinity purified by anti-FLAG affinity gel (Gescript) from whole cell lysate^21^. Purified Dot1-FLAG was incubated with 0.2 µg in vitro-assembled recombinant nucleosomes. Dot1-FLAG was then immunoprecipitated with 10 µl anti-FLAG affinity gel (Gescript) and washed with 3 × 1 ml IP washing buffer. Supernatant from the boiled beads was subject to immunoblot analysis.

### Nucleosome preparation

The recombinant *Xenopus laevis* histones H2A, H2B, H3 and H4 were expressed in *Escherichia coli* BL21 (DE3) and purified by SP ion exchange column. Octamers and nucleosomes were assembled using the salt-dialysis method^55^.

For purification of nucleosomes from yeast cells, cells were grown in 100 ml YPD medium at 30 °C until OD_600_ of ~0.7, harvested and resuspended in a 500 μl lysis buffer (10 mM Tris, pH 8.0, 50 mM NaCl, 5 mM MgCl_2_, 5 mM CaCl_2_, 0.5 mM spermidine, 1 mM 2-mercaptoethanol, 0.075% NP-40). 1.5 μl zymolyase 20 T was added to digest cell wall at 37 °C for 30 min. MNase was then added to digest chromatin into soluble nucleosomes. After centrifugation, the nucleosome-containing supernatant was collected, quantitated with Super-Bradford Quantification Kit (Beijing ComWin Biotech Co., Ltd) and stored with aliquots at −80 °C.

### Protein expression and purification

The recombinant 6×His-tagged Dot1 (Dot1-His6) and Pyk1 (Pyk1-His6) proteins were expressed in *E. coli* and purified by Ni-NTA Agarose (QIAGEN). The TAP-tagged Dot1 (Dot1-TAP) was purified from yeast cells by TAP purification^65,66^. For FLAG affinity purification of SIR complex, cells were cultured in 12 L YPD medium and collected at OD_600_ of 1.5. Cells were resuspended in 10 ml H350 buffer (25 mM HEPES-KOH, pH7.6, 350 mM KCl, 2 mM MgCl_2_, 1 mM EDTA, 10% Glycerol, 0.02% NP40, protease inhibitor cocktail) and then homogenized by Biospec bead beater. After centrifugation at 17,000 × *g* for 2 h, the cell lysate was incubated with 500 μl pre-washed anti-FLAG Affinity Gel (Gescript) at 4 °C for 4 h. The beads were washed successively with 5 ml H350 buffer and 3 ml E100 buffer (25 mM HEPES-KOH, pH7.6, 100 mM KCl, 2 mM MgCl_2_, 1 mM EDTA, 10% Glycerol, 0.02% NP40). The SIR complex was eluted with E100 buffer containing 0.25 mg/ml FLAG peptide and concentrated using Amicon Ultra-0.5 ml Centrifugal Filters (Millipore).

### In vitro histone methyltransferase (HMT) assay

The HMT reactions were performed by mixing purified 20 μg Dot1-TAP and 0.5 mg nucleosomes in the reaction buffer (50 mM Tris-HCl, pH 8.5, 50 mM NaCl, 5 mM MgCl_2_ and 1 mM DTT) with 10 mM SAM at 30 °C for 0–3 h. The reactions were quenched by adding 5×SDS buffer and incubated at 95 °C for 5 min. The products were resolved in a 10% or 18% SDS-PAGE gel and the histone methylation level was determined by immunoblotting with indicated antibodies.

### Microscopy analysis

Yeast cells were cultured in YPD or selective medium until OD_600_ of 1.0. After washing with cold phosphate-buffered saline (PBS), cells were fixed with 4% formaldehyde in PBS for 0.5 h and treated with DAPI for 15 min. Cells were then washed with cold PBS and visualized by fluorescent microscopy using a ZEISS LSM710 microscope (Germany) with a 100× oil immersion objective. Images were acquired using ZEN Imaging Software ZEN 2.1 (ZEISS). The nucleus was indicated by DAPI blue staining. The merged color Images were generated by ImageJ.

### Peptide pull-down assay

The peptide pull-down assays were performed in accordance with the protocol described^67^. In brief, 1.0 µg of biotinylated histone peptides were incubated with 1 μg of protein or 0.5 ml whole cell extract in binding buffer (50 mM Tris-HCl, pH 7.5, 250 mM NaCl, 0.1% NP-40, 1 mM PMSF, protease inhibitors) overnight at 4 °C. After 1 h incubation with Streptavidin beads (Amersham), the beads were washed with the binding buffer and boiled for 5 min. The supernatant from boiled beads was subject to immunoblot analysis. The peptides used are the following:

Unmodified H3 (1–23): ARTKQTARKSTGGKAPRKQIASK;

H3K4me3 (1–23): ARTK(me3)QTARKSTGGKAPRKQIASK;

H3pT11 (1–23): ARTKQTARKST(p)GGKAPRKQIASK;

Unmodified H3 (72–85): REIAQDFKTDLRFQ;

H3K79me3 (72–85): REIAQDFK(me3)TDLRFQ.

### Liquid chromatography-tandem mass spectrometry (LC-MS) analysis

The immunoprecipitated proteins were disulfide-reduced by 25 mM DTT at 37 °C for 40 min. Cysteines were alkylated by adding iodoacetamide to a final concentration of 50 mM. The immunoprecipitated proteins were digested with sequencing-grade trypsin (Promega) at 37 °C overnight and the supernatant was then desalted using C18 columns (Thermo Fisher) and lyophilized. The dried peptides were reconstituted in 0.1% formic acid (FA) and loaded onto an Acclaim PepMap 100 C18 LC column (Thermo Fisher) utilizing a Thermo Easy nLC 1000 LC system (Thermo Fisher) connected to Q Exactive HF mass spectrometer (Thermo Fisher)^67^. The peptides were eluted with a 5–20% gradient of acetonitrile with 0.1% formic acid over 70 min. The flow rate was 300 nl min^−1^. The MS1 scans were performed at a resolution of 60,000 over a mass range of 380–1560 *m/z*, with a maximum injection time of 120 ms and an AGC target of 1 × 10^6^. The MS2 scans were performed at a resolution of 15,000 with a normalized collision energy of 24, a maximum injection time of 50 ms and an AGC target of 2 × 10^5^. The raw mass spectrometry data were searched against the *Saccharomyces cerevisiae* proteome database from Uniport (https://www.uniprot.org/proteomes/UP000002311) using Sequest HT, MS Amanda and ptmRS algorithms in Proteome Discoverer 2.3 (Thermo Fisher). The precursor ion mass tolerance was set to 10 ppm and the fragment ion mass was 0.02 Da. Dynamic modifications were set for methionine oxidation and phosphorylation on serine, threonine and tyrosine. Only fully tryptic peptides with up to two mis-cleavages and a false-detection rate of 1% using the percolator validator algorithms were accepted. One biological replicate was performed for Reb1-FLAG IP sample.

### Subcellular fractionation

Log phase cells were harvested, washed with sterile cold H_2_O and then resuspended in 900 μl of cold buffer SB (40 mM HEPES, pH 7.5, 1.4 M Sorbitol, 0.5 mM MgCl_2_, 10 mM 2-mercaptoethanol, 1 mM PMSF). After washing, cell pellets were resuspended in buffer SB plus 2 mM 2-mercaptoethanol and 2 mg/ml Zymolase 20 T and incubated at 30 °C for 0.5 hr. The pellet was washed with sorbitol-containing buffer SB solution and resuspended in 400 μl of lysis buffer (10 mM Tris, pH 8.0, 5 mM MgCl_2_, 5 mM CaCl_2_, 0.5 mM spermidine, 1 mM 2-mecaptoethanol, 0.1% NP40, 50 mM NaCl, protease inhibitors) at 4 °C for 1 h. Half of each sample was stored at −20 °C as the whole cell extract. The other half was centrifuged at 12,000 × *g* for 5 min at 4 °C. The pellet was washed with lysis buffer and resuspended in 200 μl of lysis buffer as the chromatin fraction. Protein distribution in each fraction was detected by immunoblotting with the antibodies against GAPDH (Proteintech) and H3 (Abcam).

### Statistics and reproducibility

Representative results of at least two biologically independent experiments were performed in all of the figure panels. The two-sided Student’s *t*-test was used for comparison between two groups and a *P* value < 0.05 was considered statistically significant. For all error bars, data are mean ±  standard error (SEM). Microsoft Excel (professional Plus2013) for basic statistical analysis.

### Reporting summary

Further information on research design is available in the Nature Portfolio Reporting Summary linked to this article.

## Supplementary information

Supplemental information

Reporting Summary

## Source data

Source data

## Data Availability

The data that support this study are available from the corresponding authors upon reasonable request. The ChIP-seq data for H3K79me3 and Dot1 generated in this study have been deposited in the GEO database under accession numbers PRJNA793286 and PRJNA793621. The ChIP-seq data for H3pT11 and H3K79me3 in WT and FRB-Pyk1 generated in this study have been deposited in the GEO database under accession numbers PRJNA876639 and PRJNA876653. The ChIP-seq data for Dot1 in WT H3 and H3T11D generated in this study have been deposited in the GEO database under accession number PRJNA876760. The ChIP-seq data for H3K79me3 (WT H3, H3T11D), Reb1 and H4K16ac generated in this study have been deposited in the GEO database under accession number GSE210908. The ChIP-seq data for H3pT11 and H3K79me3 are available in the GEO database under accession numbers GSE147050 and GSE107331. The ChIP-seq data for Sir2, Sir3, and Sir4 are available in the GEO database under accession number SRP030670. The ChIP-seq data for Reb1 and Tbf1 are available in the GEO database under accession numbers GSM2143116 and GSM521935. The ChIP-seq data for H3K79me1, H3K36me, H4R3me2s, H4K5ac, H4K12ac, H4K8ac, H4R3me, H4K20me, H3K36me3, H3K36me2, H3K27ac, H3K23ac, H2AK5ac, H3K4me, H3K4me2, H3K4me3, H3K14ac, H3K18ac, H3K56ac, and H3K9ac are available in the GEO database under accession number GSE61888. The RNA-seq data for WT, H3T11A, H3K79A and *set2Δ* are available in the GEO database under accession numbers GSE147764, GSE29059, and GSE167338. The Genome file sacCer3 can be downloaded from https://hgdownload.soe.ucsc.edu/goldenPath/sacCer3/bigZips/. The mass spectrometry proteomics data have been deposited to the ProteomeXchange Consortium via the PRIDE partner repository with the dataset identifier PXD038138. Source data are provided with this paper.
