# Author Correction: SESAME-catalyzed H3T11 phosphorylation inhibits Dot1-catalyzed H3K79me3 to regulate autophagy and telomere silencing

**DOI:** 10.1038/s41467-026-78553-2

**Published:** 2026-10-19

**Authors:** Fei He, Qi Yu, Min Wang, Rongsha Wang, Xuanyunjing Gong, Feng Ge, Xilan Yu, Shanshan Li

**Affiliations:** 1 State Key Laboratory of Biocatalysis and Enzyme Engineering, School of Life Sciences, Hubei University, Wuhan, Hubei 430062 China; 2 Key Laboratory of Algal Biology, Institute of Hydrobiology, Chinese Academy of Sciences, Wuhan, Hubei 430072 China

**Keywords:** Epigenetics, Autophagy, Epigenetics, Enzymes

Correction to: *Nature Communications* https://doi.org/10.1038/s41467-022-35182-9, published online 6 December 2022

In the version of this article initially published, in Fig. 4b and associated Source Data, data for *ATG7*, *ATG15* and *ATG23* were incorrect; in Fig. 6l and associated Source Data, data for the 16K-Replicate 3 were inadvertent duplicates of 25K-Replicate 3 data; in Supplementary Fig. 8c and associated Source Data, data for *UBA2* and *FAR3* from Replicate 3 were inadvertently duplicated into Replicate 2. For comparison, the original figures are available as Supplementary Information accompanying this amendment. The figures and Source Data are now amended in the HTML and PDF versions of the article.

## Supplementary information

Original Figs. 4, 6, Supplementary Fig. 8

