# Supplementary material for: SESAME-catalyzed H3T11 phosphorylation inhibits Dot1-catalyzed H3K79me3 to regulate autophagy and telomere silencing
Source: Nat Commun. 2022 Dec 6;13:7526. doi: 10.1038/s41467-022-35182-9 (PMC9726891; doi:10.1038/s41467-022-35182-9)
Supplement: Supplementary file 1 — Supplemental information [file 41467_2022_35182_MOESM1_ESM.pdf]

Supplementary Information

SESAME-catalyzed H3T11 phosphorylation inhibits Dot1-catalyzed H3K79me3 to regulate autophagy and telomere silencing

Fei He[1,3], Qi Yu[1,3], Min Wang[2], Rongsha Wang[1], Xuanyunjing Gong[1], Feng Ge[2], Xilan Yu[1,*], Shanshan Li[1,*]

[1]State Key Laboratory of Biocatalysis and Enzyme Engineering, School of Life Sciences, Hubei University, Wuhan, Hubei 430062, China

[2]Key Laboratory of Algal Biology, Institute of Hydrobiology, Chinese Academy of Sciences, Wuhan, Hubei 430072, China

[3]These authors contribute equally to this work

*Corresponding authors: Shanshan Li, shl@hubu.edu.cn

Xilan Yu, yuxilan@hubu.edu.cn

Supplementary Data:

1. Supplementary (Fig. 1-14).

2. Supplemental Tables (Table S1-2).

Supplementary Fig. 1

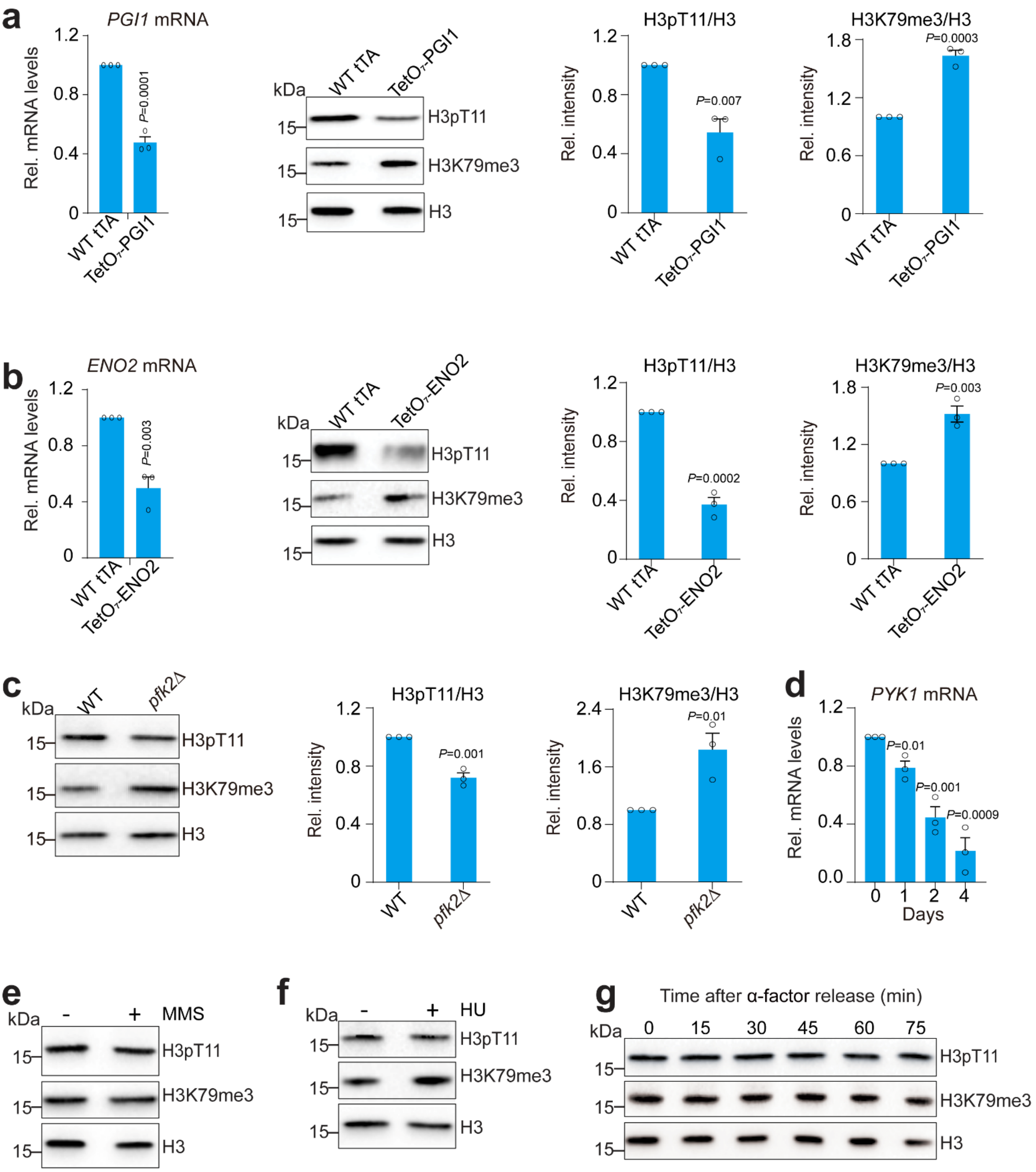

Supplementary Fig. 1. H3pT11 anti-correlates with H3K79me3.
a, Immunoblot analysis of H3pT11 and H3K79me3 in WT tTA and $TetO_7$-*PGI1* mutant. Cells were grown in YPD medium until OD_{600} of 0.5 and then treated with 40 µg/ml doxycycline for 3 hr. The knockdown efficiency of *PGI1* was determined by RT-qPCR.
b, Immunoblot analysis of H3pT11 and H3K79me3 in WT tTA and $TetO_7$–*ENO2* mutant. Cells were grown in YPD medium until OD_{600} of 0.5 and then treated with 40 µg/ml doxycycline for 3 hr. The knockdown efficiency of *ENO2* was determined by RT-qPCR.
c, Immunoblot analysis of H3pT11 and H3K79me3 in WT and *pfk2Δ* mutant.
d, The transcription of *PYK1* was reduced during chronological aging. Cells were grown in YPD medium for 0-4 days to undergo chronological aging. The total RNA was isolated and the transcription of *PYK1* was determined by RT-qPCR.
e, Immunoblot analysis of H3pT11 and H3K79me3 in WT cells treated with or without methyl methanesulfonate (MMS). Cells were grown in YPD medium until OD_{600} of 0.5 and then treated with or without 0.02% MMS for 1 hr.
f, Immunoblot analysis of H3pT11 and H3K79me3 in WT cells treated with or without hydroxyurea (HU). Cells were grown in YPD medium until OD_{600} of 0.5 and then treated with or without 10 mM HU for 2 hr.
g, Immunoblot analysis of H3pT11 and H3K79me3 in WT cells synchronized at G1/S with 4 µg/ml α factor for 2 hr and then released for 0-75 min. Cell cycle arrest was achieved as demonstrated by flow cytometry.

For Supplementary Fig. 1**a-d**, data represent the mean ± SE; n=3 biologically independent experiments. Two-sided *t*-tests were used for statistical analysis. For Supplementary Fig. 1**e-g**, a typical example of three biologically independent replicates was shown.

Supplementary Fig. 2

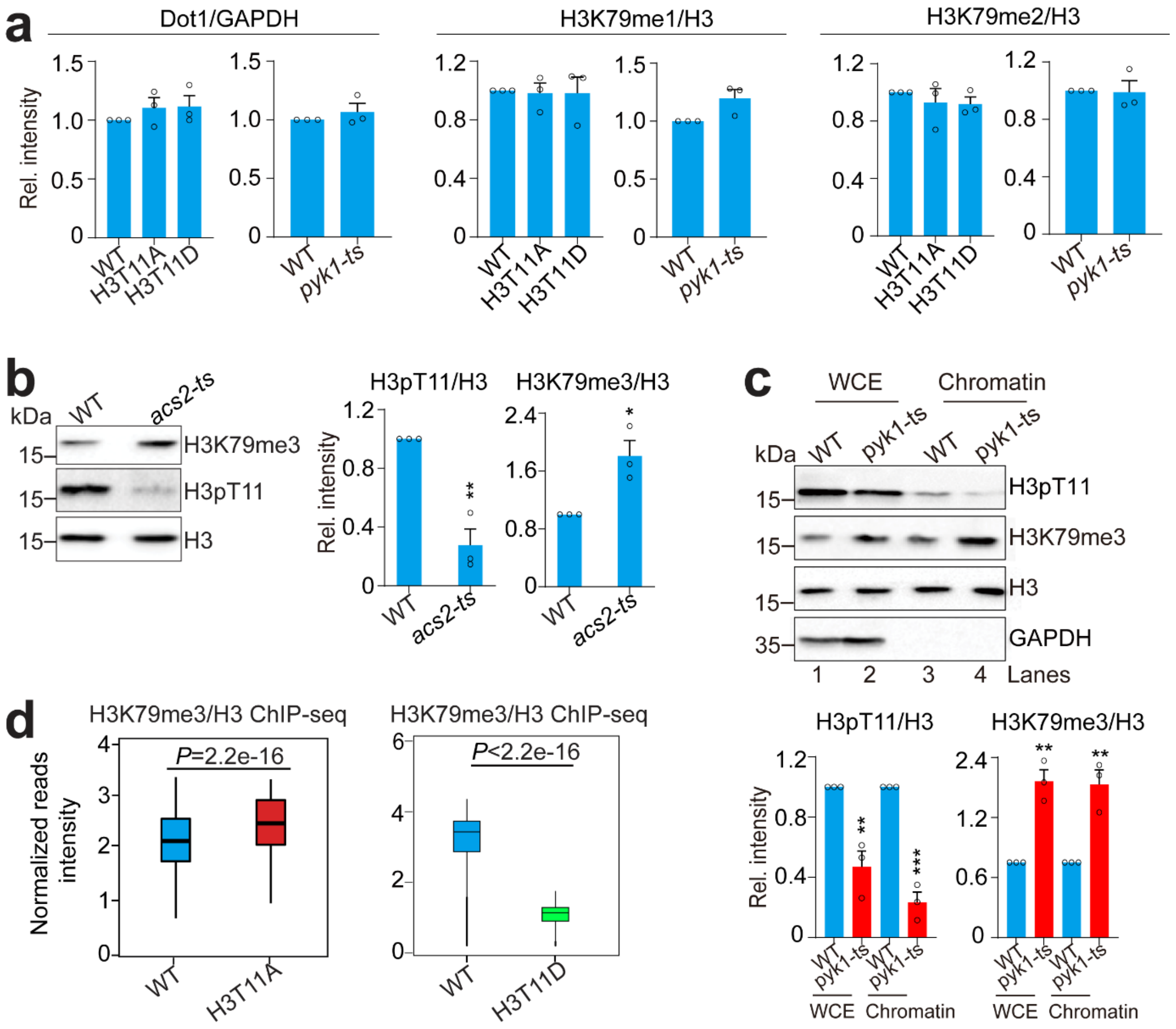

Supplementary Fig. 2. Loss of H3pT11 increases Dot1-catalyzed H3K79me3.

a, Quantification of the relative intensity of Dot1/GAPDH, H3K79me1/H3 and H3K79me2/H3 in Fig. 2**a**-**b**.

b, Immunoblot analysis of H3pT11 and H3K79me3 in WT and *acs2-ts* mutant. WT and *acs2-ts* mutant were grown in YPD medium until OD_{600} of 0.5 and then treated at 39 °C for 2 hr.

c, Effects of Pyk1 on H3pT11 and H3K79me3 in the whole cell extract (WCE) and chromatin-bound fraction by immunoblots. WT and *pyk1-ts* mutant were grown in YPD medium until OD_{600} of 0.5 and then treated with 39 °C for 2 hr.

d, Box plots of normalized H3K79me3/H3 reads intensity in WT, H3T11A and H3T11D mutants. Centre lines denote medians; box limits denote 25th and 75th percentiles; whiskers denote maxima and minima. Two-sided Wilcoxon test in R (package ggpval) was used for statistical analysis.

For Supplementary Fig. 2**a**-**c**, data represent the mean ± SE; n=3 biologically independent experiments. Two-sided *t*-tests were used for statistical analysis.

Supplementary Fig. 3

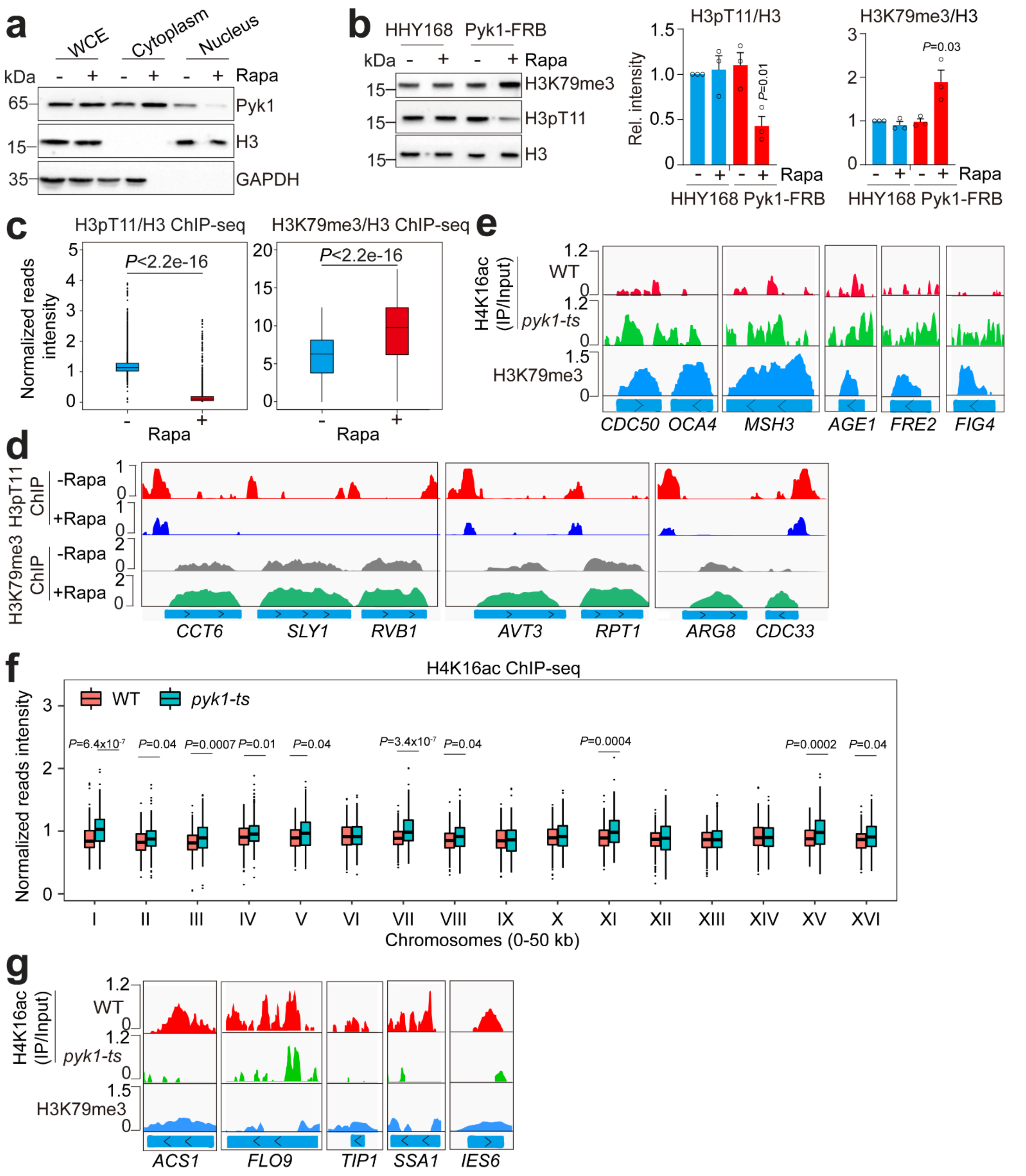

Supplementary Fig. 3. Effect of SESAME-catalyzed H3pT11 on H4K16ac and H3K79me3.
a, Subcellular fractionation assay showing reduced nuclear Pyk1 in Pyk1-FRB mutant when treated with 1 μg/ml rapamycin for 2 hr.
b, Blocking Pyk1 nuclear translocation reduced H3pT11 and increased H3K79me3. WT (HHY168) and Pyk1-FRB mutant were treated with or without 1 μg/ml rapamycin for 2 hr.
c, Box plots showing the normalized reads intensity of H3pT11/H3 and H3K79me3/H3 in Pyk1-FRB mutant when treated with or without 1 μg/ml rapamycin. Centre lines denote medians; box limits denote 25th and 75th percentiles; whiskers denote maxima and minima. Two-sided Wilcoxon test in R (package ggpval) was used for statistical analysis.
d, ChIP-seq tracks showing the enrichment of H3pT11/H3 and H3K79me3/H3 at representative genes in Pyk1-FRB mutant when treated with or without rapamycin.
e, ChIP-seq tracks showing the enrichment of H4K16ac at representative genes in WT and *pyk1-ts* mutant. WT and *pyk1-ts* mutant were grown in YPD medium until OD_{600} of 0.5 and then treated with 39 °C for 2 hr.
f, Box plots showing the normalized reads intensity of H4K16ac at subtelomeric regions (<50 kb from the nearest telomeres) in WT and *pyk1-ts* mutant. Centre lines denote medians; box limits denote 25th and 75th percentiles; whiskers denote maxima and minima.
g, ChIP-seq tracks showing the enrichment of H4K16ac at representative genes in WT and *pyk1-ts* mutant.

For Supplementary Fig. 3**b**, data represent the mean ± SE; n=3 biologically independent experiments. Two-sided *t*-tests were used for statistical analysis. For Supplementary Fig. 3**a**, a typical example of two biologically independent replicates was shown.

Supplementary Fig. 4

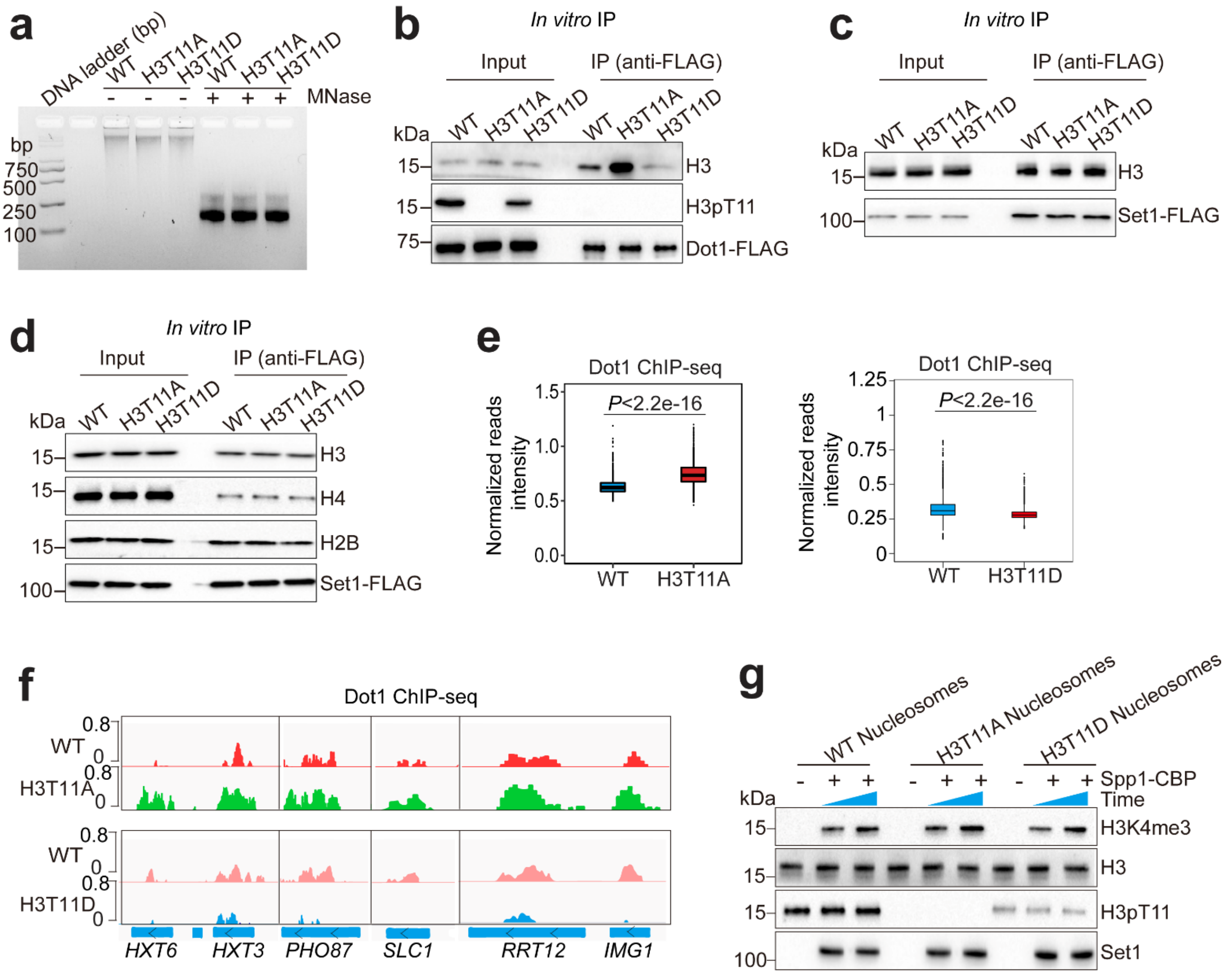

Supplementary Fig. 4. SESAME-catalyzed H3pT11 antagonizes Dot1-catalyzed H3K79me3.

a, Profile of MNase-digested nucleosomes. Nucleosomes were isolated from WT, H3T11A and H3T11D mutants followed by digestion with MNase. The digested nucleosomes were electrophoresed on 1% agarose gel.

b, *In vitro* Co-IP showing purified Dot1-FLAG preferentially bound recombinant purified histone H3T11A. Purified Dot1-FLAG showed less binding affinity to recombinant purified histone H3T11D. Dot1-FLAG was purified from yeast cells with anti-FLAG beads.

c, *In vitro* Co-IP showing purified Set1-FLAG had no preference to bind recombinant purified WT H3, H3T11A, and H3T11D mutant histones. Set1-FLAG was purified from yeast cells with anti-FLAG beads.

d, *In vitro* Co-IP showing purified Set1-FLAG had no preference to bind WT H3, H3T11A, and H3T11D nucleosomes.

e, Box plots showing the normalized reads intensity of Dot1 in WT, H3T11A, and H3T11D mutants. Centre lines denote medians; box limits denote 25th and 75th percentiles; whiskers denote maxima and minima. Two-sided Wilcoxon test in R (package ggpval) was used for statistical analysis.

f, ChIP-seq tracks showing the occupancy of Dot1 at representative genes in WT, H3T11A and H3T11D mutants.

g, *In vitro* histone methyltransferase activity with tandem affinity purified Set1 complex (Spp1-CBP) and nucleosomes. Set1 complex had similar activity towards WT, H3T11A and H3T11D nucleosomes.

For Supplementary Fig. 4**a**-**d**, **g**, a typical example of two biologically independent replicates was shown.

Supplementary Fig. 5

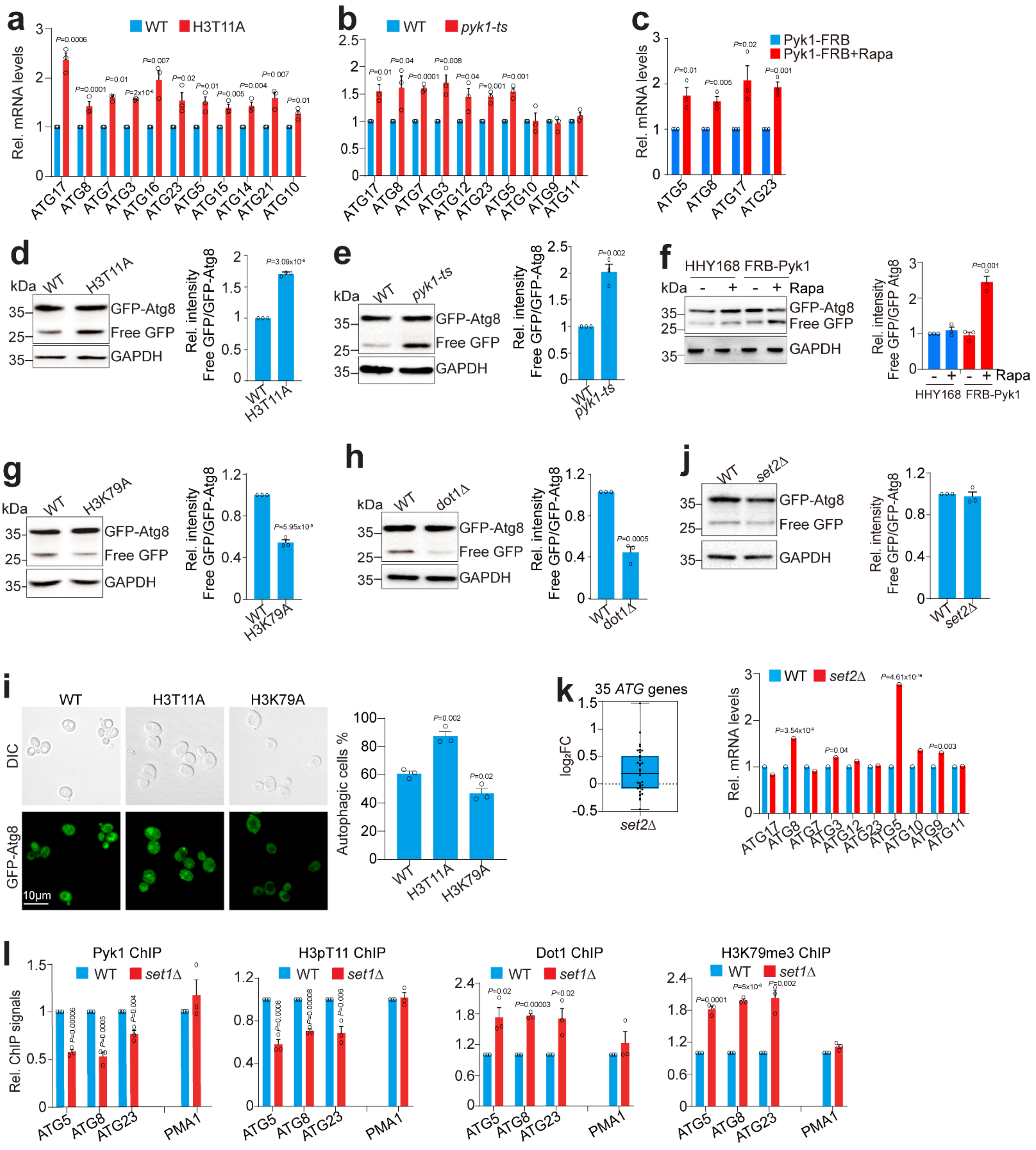

Supplementary Fig. 5. SESAME-catalyzed H3pT11 represses autophagy by inhibiting Dot1-catalyzed H3K79me3.

a-**b**, RT-qPCR analysis of the transcription of autophagy genes in WT, H3T11A, and *pyk1-ts* mutants. For supplementary Fig. 5**b**, WT and *pyk1-ts* mutant were grown in YPD medium until OD_{600} of 0.5 and then treated with 39 °C for 2 hr.

c, RT-qPCR analysis of the transcription of autophagy genes in Pyk1-FRB mutant treated with or without 1 µg/ml rapamycin for 2 hr.

d, GFP-Atg8 processing assays were performed in WT and H3T11A mutant expressing the endogenous *ATG8* promoter-driven *GFP-ATG8*. The ratio of free GFP/GFP-Atg8 was used to indicate the autophagy activity.

e, GFP-Atg8 processing assays were performed in WT and *pyk1-ts* mutant expressing the endogenous *ATG8* promoter-driven *GFP-ATG8*. WT and *pyk1-ts* mutant were grown in YPD medium until OD_{600} of 0.5 and then treated with 39 °C for 2 hr.

f, GFP-Atg8 processing assays were performed in WT (HHY168) and Pyk1-FRB mutant expressing the endogenous *ATG8* promoter-driven *GFP-ATG8* treated with or without 1 µg/ml rapamycin for 2 hr.

g-h, GFP-Atg8 processing assays were performed in WT, H3K79A and *dot1Δ* mutants expressing the endogenous *ATG8* promoter-driven *GFP-ATG8*.

i, Representative fluorescence microscopy images showing the distribution of GFP-Atg8 (green) in WT, H3T11A and H3K79A mutants.

j, Set2 had no significant effect on autophagy. GFP-Atg8 processing assays were performed in WT and *set2Δ* mutant expressing the endogenous *ATG8* promoter-driven *GFP-ATG8*.

k, Left panel, box plots showing the transcriptional changes of *ATG* genes in *set2Δ* by analyzing *set2Δ* RNA-seq data. FC, fold change (*set2Δ*/WT). Right panel, bar graph showing the transcription of *ATG* genes in *set2Δ* by analyzing *set2Δ* RNA-seq data. Centre lines denote medians; box limits denote 25th and 75th percentiles; whiskers denote maxima and minima.

l, ChIP-qPCR analysis of the enrichment of Pyk1, H3pT11, Dot1 and H3K79me3 at *ATG5*, *ATG8* and *ATG23* in WT and *set1Δ* mutant.

For Supplementary Fig. 5**a**-**l**, data represent the mean ± SE; n=3 biologically independent experiments. Two-sided *t*-tests were used for statistical analysis.

Supplementary Fig. 6

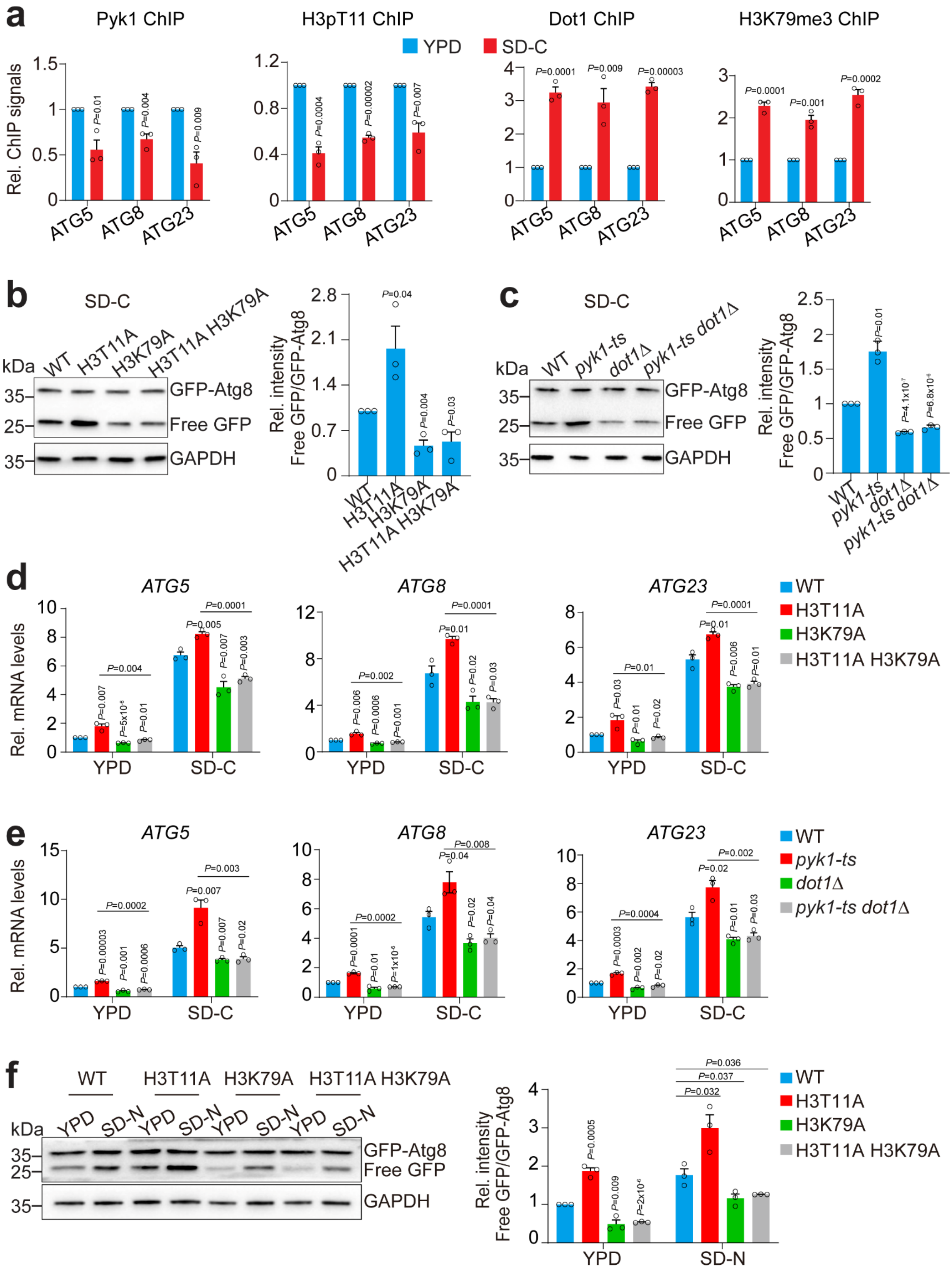

Supplementary Fig. 6. SESAME-catalyzed H3pT11 represses autophagy by inhibiting Dot1-catalyzed H3K79me3 under glucose starvation conditions.

a, ChIP-qPCR analysis of the enrichment of Pyk1, H3pT11, Dot1 and H3K79me3 at *ATG5*, *ATG8* and *ATG23* when cells were grown in YPD rich media and glucose starvation condition (SD-C) for 2 hr, respectively.

b, Representative immunoblot analysis of GFP-Atg8 and free GFP in WT, H3T11A, H3K79A and H3T11A H3K79A mutants expressing the endogenous *ATG8* promoter-driven *GFP-ATG8* with anti-GFP antibody. Cells were grown in glucose starvation medium (SD-C) for 2 hr. GAPDH was used as a loading control.

c, GFP-Atg8 processing assays were performed in WT, *pyk1-ts*, *dot1Δ* and *pyk1-ts dot1Δ* mutants expressing the endogenous *ATG8* promoter-driven *GFP-ATG8*. Cells were grown in glucose starvation medium (SD-C) at 39 °C for 2 hr.

d, RT-qPCR analysis of the transcription of autophagy genes in WT, H3T11A, H3K79A and H3T11A H3K79A mutants. Cells were grown in YPD rich medium and glucose starvation medium (SD-C), respectively.

e, RT-qPCR analysis of the transcription of autophagy genes in WT, *pyk1-ts*, *dot1Δ* and *pyk1-ts dot1Δ* mutants. Cells were grown in YPD rich medium and glucose starvation medium (SD-C) at 39 °C for 2 hr, respectively.

f, GFP-Atg8 processing assays were performed in WT, H3T11A, H3K79A and H3T11A H3K79A mutants expressing the endogenous *ATG8* promoter-driven *GFP-ATG8*. Cells were grown in YPD rich medium and nitrogen starvation medium (SD-N) for 1 hr, respectively.

For Supplementary Fig. 6**a**-**f**, data represent the mean ± SE; n=3 biologically independent experiments. Two-sided *t*-tests were used for statistical analysis.

Supplementary Fig. 7

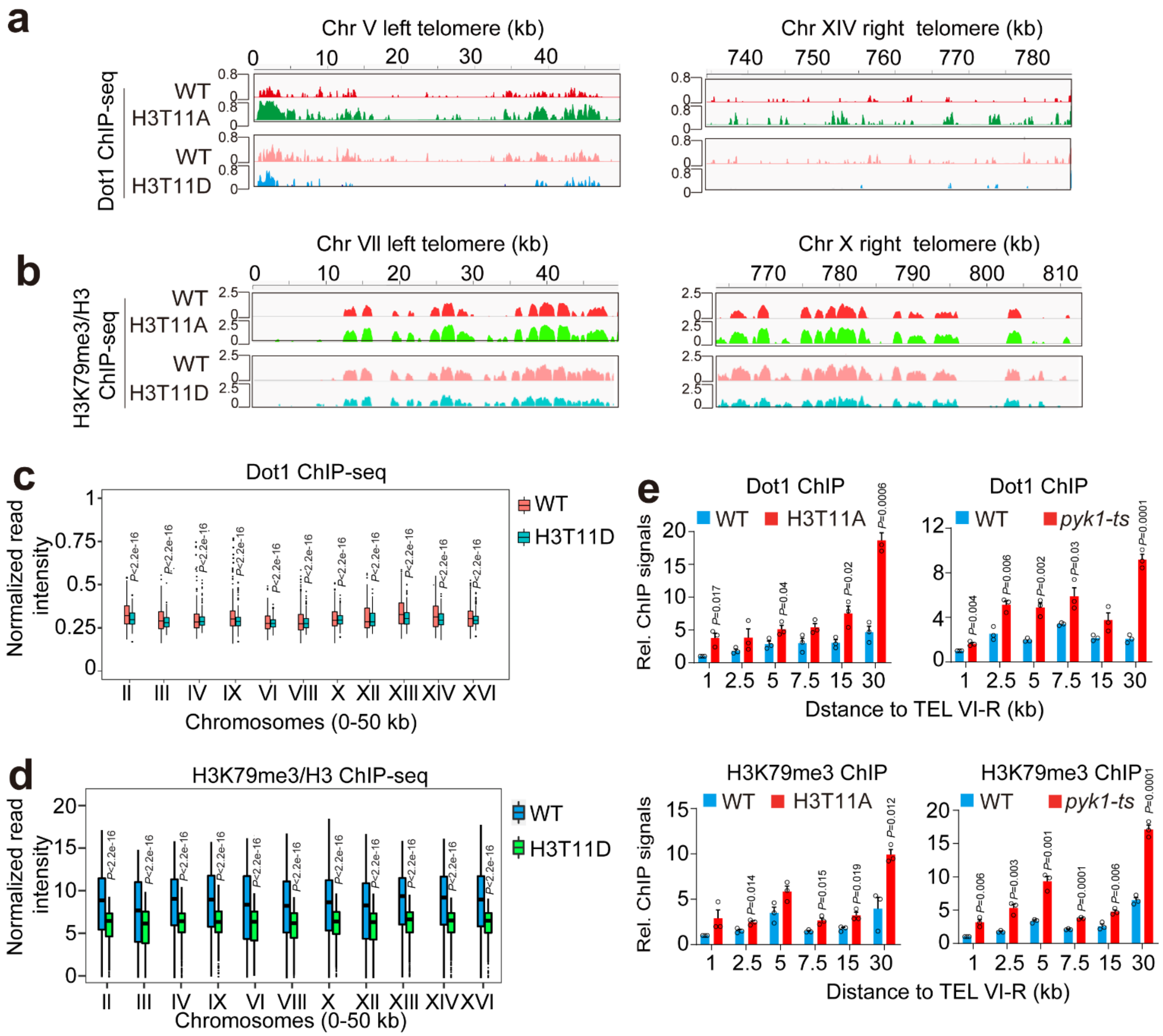

Supplementary Fig. 7. SESAME-catalyzed H3pT11 and Dot1-catalyzed H3K79me3 work together to promote telomere silencing.
a, ChIP-seq tracks showing the occupancy of Dot1 at representative subtelomeric regions in WT, H3T11A and H3T11D mutants.
b, ChIP-seq tracks showing the enrichment of H3K79me3/H3 at representative subtelomeric regions in WT, H3T11A and H3T11D mutants.
c, Box plots showing the normalized reads intensity of Dot1 at subtelomeric regions (<50 kb from the nearest telomeres) in WT and H3T11D mutant.
d, Box plots showing the normalized reads intensity of H3K79me3/H3 at subtelomeric regions (<50 kb from the nearest telomeres) in WT and H3T11D mutant.
e, ChIP-qPCR analysis of the occupancy of Dot1 and H3K79me3/H3 at regions with different distance (1, 2.5, 5, 7.5, 15 and 30 kb) to telomere VI-R in WT, H3T11A, and *pyk1-ts* mutants.

For Supplementary Fig. 7**e**, data represent the mean $\pm$ SE; n=3 biologically independent experiments. Two-sided *t*-tests were used for statistical analysis. For Supplementary Fig. 7**c-d**, centre lines denote medians; box limits denote 25th and 75th percentiles; whiskers denote maxima and minima. Two-sided Wilcoxon test in R (package ggpval) was used for statistical analysis.

Supplementary Fig. 8

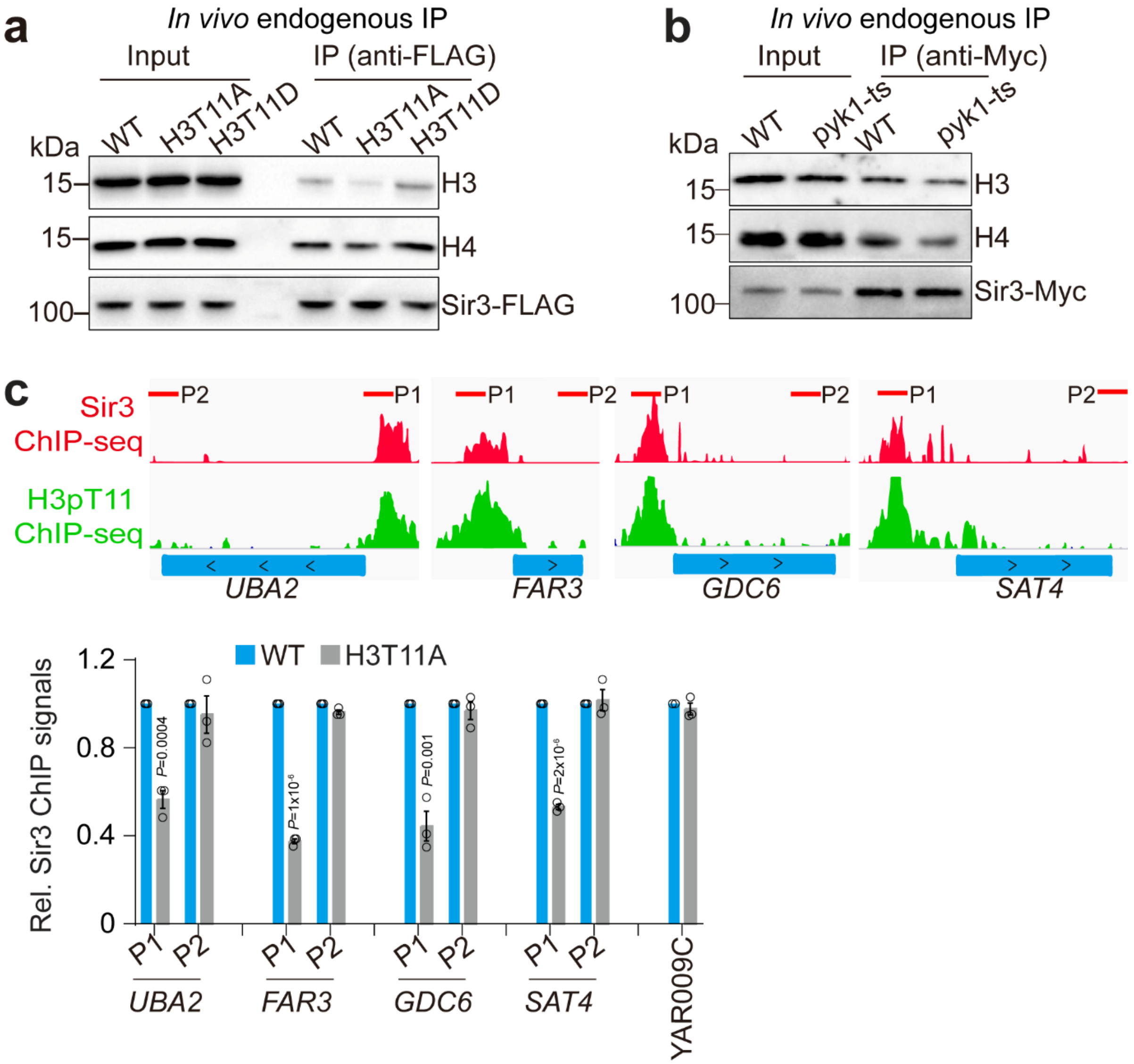

Supplementary Fig. 8. SESAME-catalyzed H3pT11 promotes the binding of SIR complex at telomere regions.

a-b, *In vivo* endogenous Co-IP assays showing SESAME-catalyzed H3pT11 facilitates SIR complex binding to chromatin. **a**, Endogenously expressed Sir3-FLAG was immunoprecipitated from WT, H3T11A and H3T11D mutants with anti-FLAG beads. **b**, Endogenously expressed Sir3-Myc was immunoprecipitated from WT and *pyk1-ts* mutant with anti-Myc beads. Cells were grown in YPD medium at 39 °C for 2 hr.

c, ChIP-qPCR analysis of Sir3 occupancy at indicated regions in WT and H3T11A mutant. The primers used to amplify the regions were indicated at the top of ChIP-seq tracks in red lines.

For Supplementary Fig. 8**c**, data represent the mean ± SE; n=3 biologically independent experiments. Two-sided *t*-tests were used for statistical analysis. For Supplementary Fig. 8**a-b**, a typical example of two biologically independent replicates was shown.

Supplementary Fig. 9

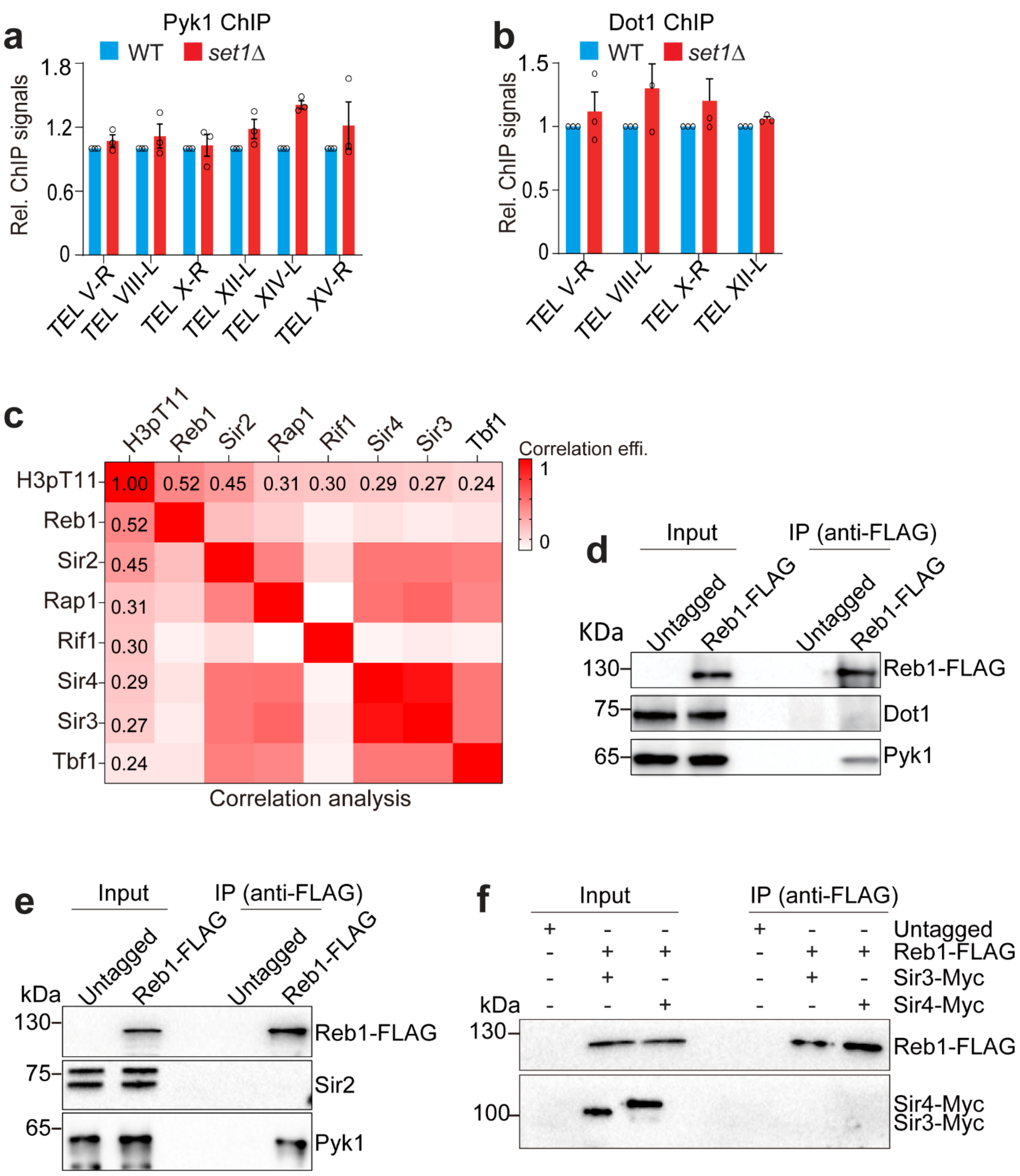

Supplementary Fig. 9. Reb1 recruits SESAME to phosphorylate H3T11 and inhibit Dot1 to catalyze H3K79me3 at telomeric regions.

a-b, ChIP-qPCR analysis of the occupancy of Pyk1 and Dot1 at subtelomeric regions in WT and *set1Δ* mutants.

c, Correlation analyses of ChIP-seq data of H3pT11 with the telomere-associated proteins. The raw ChIP-seq dataset for Reb1 is retrieved from GSE81112.

d, *In vivo* Co-IP assays showing Reb1 interacted with Pyk1 but not Dot1. The endogenously expressed Reb1-FLAG was immunoprecipitated with anti-FLAG beads. The untagged strain was used as a negative control.

e-f, *In vivo* Co-IP assays showing Reb1 had no interaction with Sir2, Sir3 and Sir4. The endogenously expressed Reb1-FLAG was immunoprecipitated with anti-FLAG beads. The untagged strain was used as a negative control.

For Supplementary Fig. 9**a-b**, data represent the mean ± SE; n=3 biologically independent experiments. Two-sided *t*-tests were used for statistical analysis. For Supplementary Fig. 9**d-f**, a typical example of two biologically independent replicates was shown.

Supplementary Fig. 10

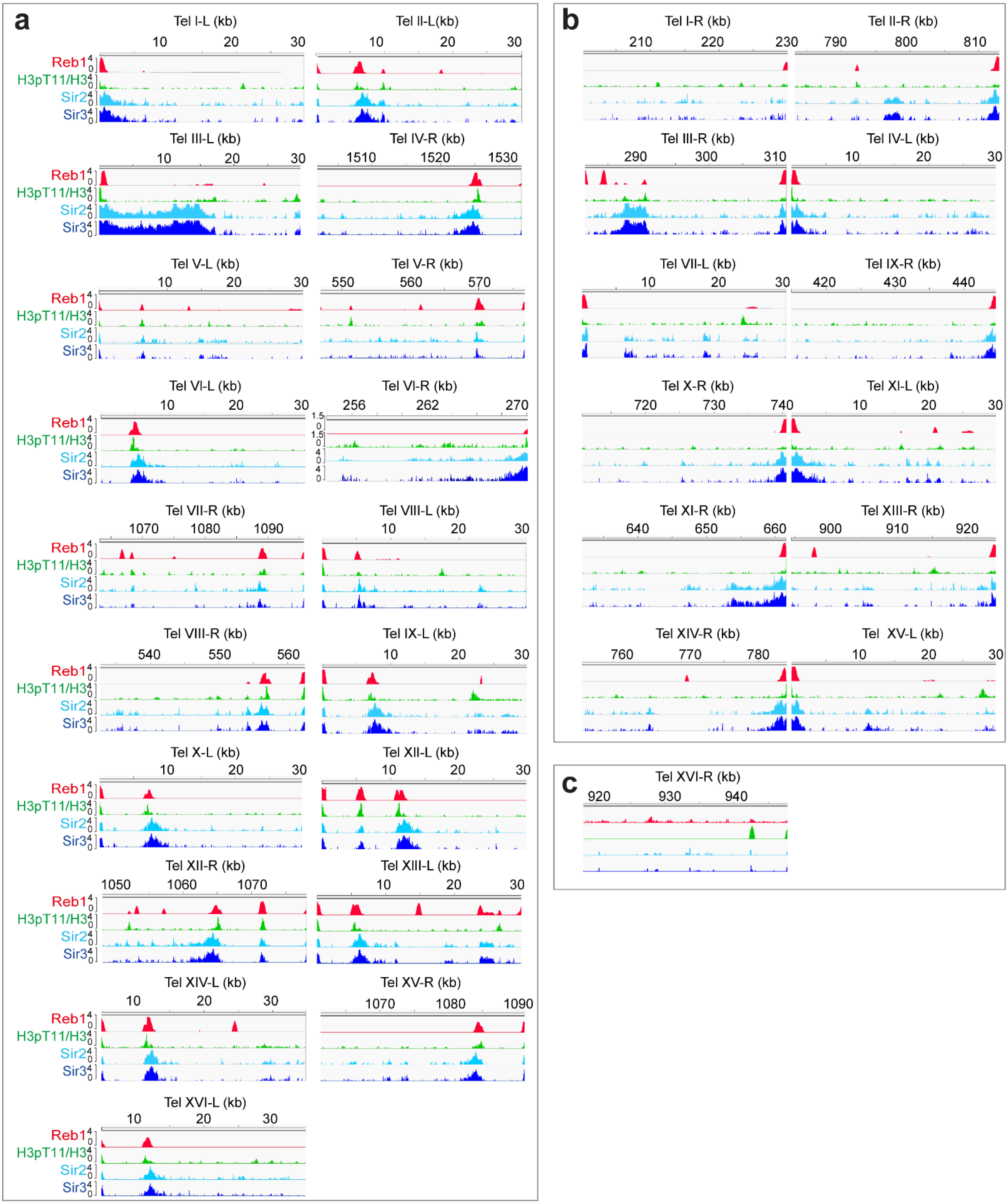

Supplementary Fig. 10. The localization of Reb1, H3pT11 and SIR complex at telomere-proximal regions.

a, Reb1, H3pT11 and SIR complex co-localized at 19 subtelomere regions, including Tel I-L, Tel II-L, Tel III-L, Tel IV-R, Tel V-L, Tel V-R, Tel VI-L, Tel VI-R, Tel VII-R, Tel VIII-L, Tel VIII-R, Tel IX-L, Tel X-L, Tel XII-L, Tel XII-R, Tel XIII-L, Tel XIV-L, Tel XV-R, Tel XVI-L.

b, Reb1 occupied at 12 subtelomere regions where the H3pT11 enrichment was low, including Tel I-R, Tel II-R, Tel III-R, Tel IV-L, Tel VII-L, Tel IX-R, Tel X-R, Tel XI-L, Tel XI-R, Tel XIII-R, Tel XIV-R, Tel XV-L.

c, An example of a telomere (Tel XVI-R) enriched in H3pT11 with little enrichment of Reb1.

Supplementary Fig. 11

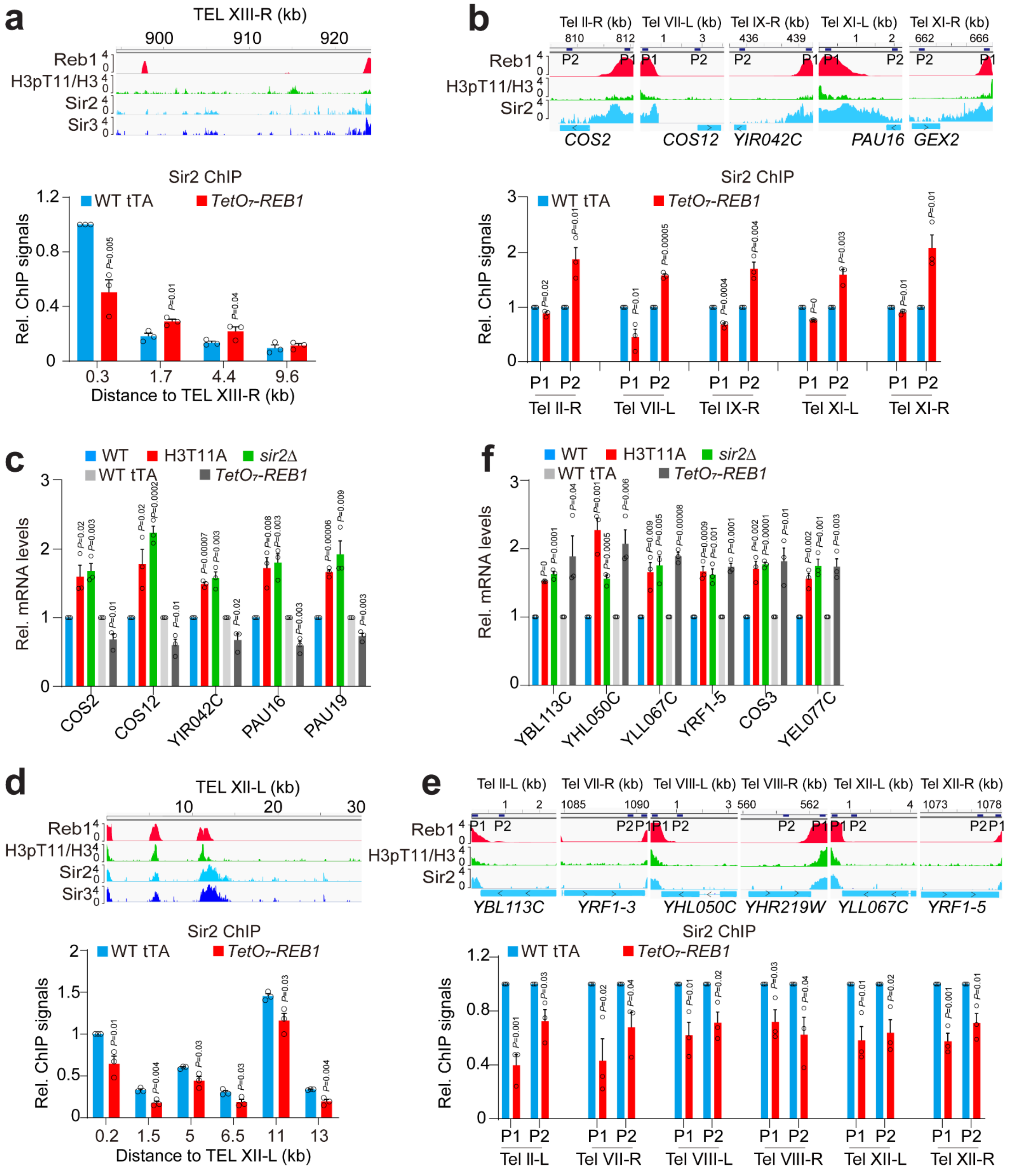

Supplementary Fig. 11. The effect of Reb1 and H3pT11 on the occupancy of SIR complex and telomere silencing differs among different telomeres.

a-b, ChIP-qPCR analysis of Sir2 occupancy at indicated subtelomeric regions in WT tTA and $TetO_7$-*REB1* mutant. WT tTA and $TetO_7$-*REB1* mutant were grown in YPD medium to an OD_{600} of 0.5 and then treated with 120 µg/ml doxycycline for 3 hr. The primers used for ChIP-qPCR were indicated on the top of ChIP-seq tracks.

c, RT-qPCR analysis of the transcription of telomere-proximal genes in WT, H3T11A, *sir2Δ*, WT tTA, and $TetO_7$-*REB1* mutants. The localization of *COS2*, *COS12*, *YIR042C*, and *PAU16* at chromosomes was indicated in the ChIP-seq tracks in Supplementary Fig. 11**b**. *PAU19* is located 1.5 kb from Tel XIII-R.

d, ChIP-qPCR analysis of Sir2 occupancy at regions with different distances to TEL XII-L in WT tTA and $TetO_7$-*REB1* mutant.

e, ChIP-qPCR analysis of Sir2 occupancy at indicated subtelomeric regions in WT tTA and $TetO_7$-*REB1* mutant.

f, RT-qPCR analysis of the transcription of telomere-proximal genes in WT, H3T11A, *sir2Δ*, WT tTA, and $TetO_7$-*REB1* mutants. The localization of *YBL113C*, *YHL050C*, *YLL067C*, and *YFR1-5* at telomeres was indicated in the ChIP-seq tracks in Supplementary Fig. 11**e**. *COS3* and *YEL077C* are located 7.22 kb from Tel XIII-L and 0.5 kb from Tel V-L, respectively.

For Supplementary Fig. 11**a**-**f**, data represent the mean ± SE; n=3 biologically independent experiments. Two-sided *t*-tests were used for statistical analysis.

Supplementary Fig. 12

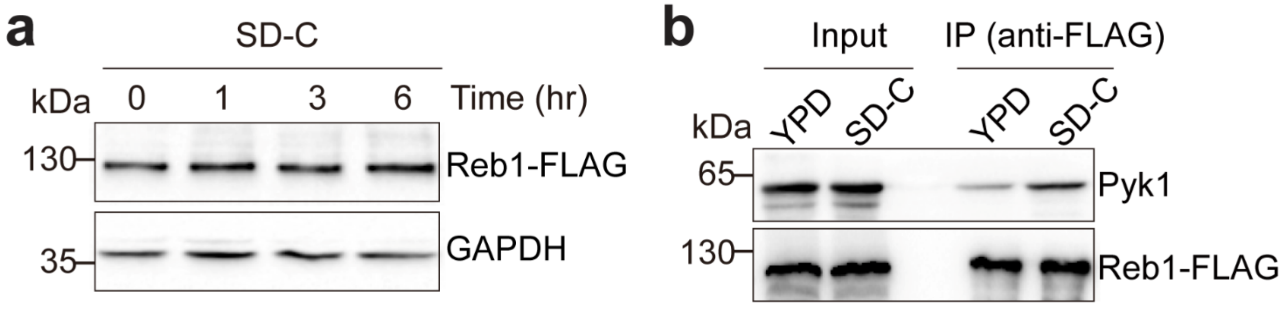

Supplementary Fig. 12. Effect of glucose starvation on Reb1 expression and Reb1-Pyk1 interaction.

a, Immunoblot analysis of Reb1 expression when cells were grown in SD-C for 0-6 hr.

b, *In vivo* Co-IP analysis of the interaction between Reb1 and Pyk1 when cells were grown in YPD and SD-C medium. The endogenous expressed Reb1-FLAG was immunoprecipitated with anti-FLAG beads.

For Supplementary Fig. 12**a**-**b**, a typical example of three biologically independent replicates was shown.

Supplementary Fig. 13

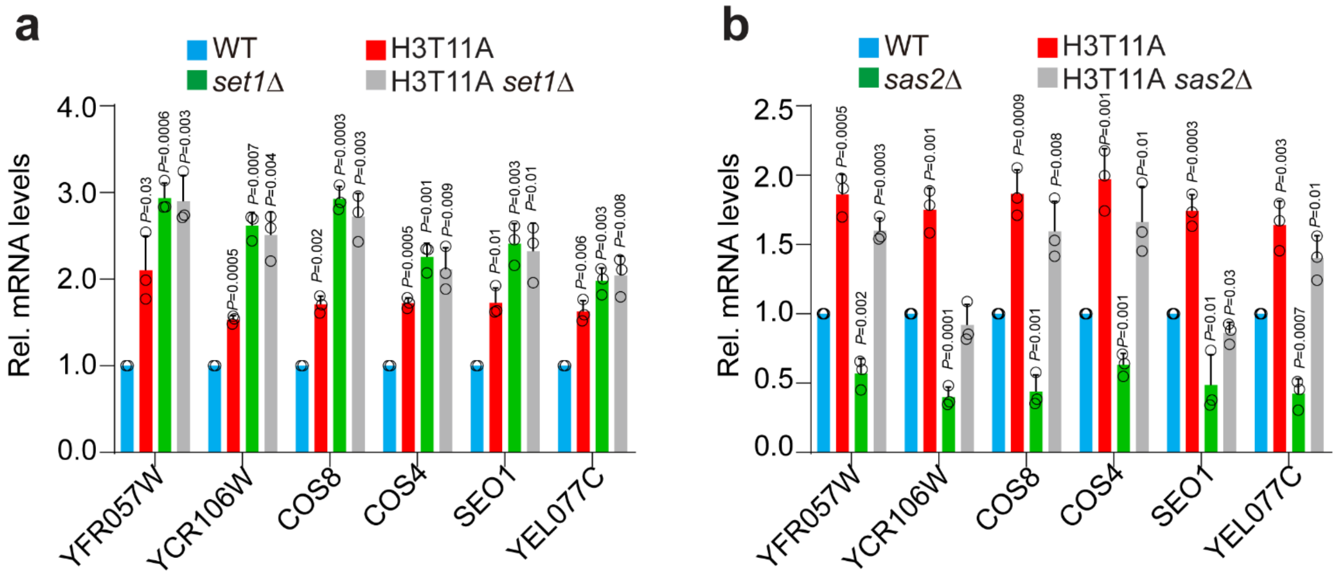

Supplementary Fig. 13. H3pT11 has no additive effect on telomere silencing with Set1 and Sas2.
a, RT-qPCR analysis of the transcription of telomere-proximal genes in WT, H3T11A, *set1Δ* and H3T11A *set1Δ* mutants.
b, RT-qPCR analysis of the transcription of telomere-proximal genes in WT, H3T11A, *sas2Δ* and H3T11A *sas2Δ* mutants.

For Supplementary Fig. 13**a**-**b**, data represent the mean ± SE; n=3 biological independent experiments. Two-sided *t*-tests were used for statistical analysis.

Supplementary Fig. 14

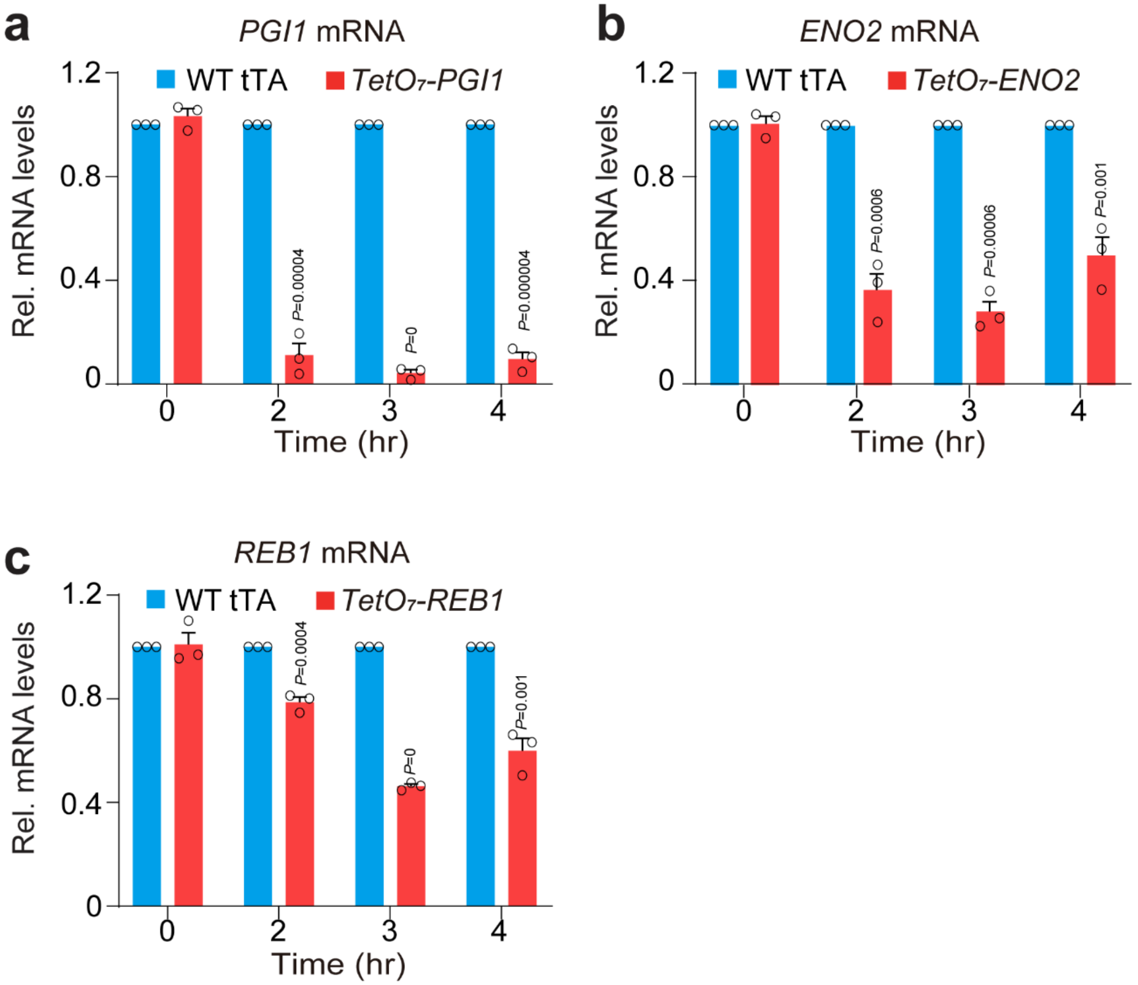

Supplementary Fig. 14. Knockdown efficiency of *PGI1*, *ENO2* and *REB1* in WT tTA, $TetO_7$-*PGI1*, $TetO_7$-*ENO2* and $TetO_7$-*REB1* mutants. WT tTA, $TetO_7$-*PGI1*, and $TetO_7$-*ENO2* mutants were grown in YPD medium to an OD_{600} of 0.5 and then treated with 40 µg/ml doxycycline for different time points from 0-4 hr. WT tTA, and $TetO_7$-*REB1* mutant were grown in YPD medium to an OD_{600} of 0.5 and then treated with 120 µg/ml doxycycline for different time points from 0-4 hr.

For Supplementary Fig. 14**a-c**, data represent the mean ± SE; n=3 biologically independent experiments. Two-sided *t*-tests were used for statistical analysis.

Supplementary Table 1 List of strains used in this study

Name	Parental Strain	Genotype	Source
WT (YBL)	S288C	*MATa his3Δ200 leu2Δ1 ura3-52, trp1Δ63 lys2-128δ (hht1- hhf1)Δ::LEU2, (hht2-hhf2)Δ::HIS3 Ty912 35-lacZ::his4 pDM18-HHT2-HHF2-TRP1*	Provided by Dr. Fred Winston
H3T11A (YBL)	S288C	*MATa his3Δ200 leu2Δ1 ura3-52, trp1Δ63 lys2-128δ (hht1- hhf1)Δ::LEU2, (hht2-hhf2)Δ::HIS3 Ty912 35-lacZ::his4 pDM18-HHT2(T11A)-HHF2-TRP1*	In this study
H3T11D (YBL)	S288C	*MATa his3Δ200 leu2Δ1 ura3-52, trp1Δ63 lys2-128δ (hht1- hhf1)Δ::LEU2, (hht2-hhf2)Δ::HIS3 Ty912 35-lacZ::his4 pDM18-HHT2(T11D)-HHF2-TRP1*	In this study
H3K79A (YBL)	S288C	*MATa his3Δ200 leu2Δ1 ura3-52, trp1Δ63 lys2-128δ (hht1- hhf1)Δ::LEU2, (hht2-hhf2)Δ::HIS3 Ty912 35-lacZ::his4 pDM18-HHT2(K79A)-HHF2-TRP1*	In this study
H3T11A H3K79A (YBL)	S288C	*MATa his3Δ200 leu2Δ1 ura3-52, trp1Δ63 lys2-128δ (hht1- hhf1)Δ::LEU2, (hht2-hhf2)Δ::HIS3 Ty912 35-lacZ::his4 pDM18-HHT2(T11A K79A)-HHF2-TRP1*	In this study
UCC1369	UCC1369	*MATa ade2::hisG his3Δ200 leu2Δ0 lys2Δ0 met15Δ0 trp1Δ63 ura3Δ0 adh4::URA3-TEL-VIIL ADE2-TEL-VR , (hht2-hhf2)Δ::MET15 (hht1-hhf1)Δ::LEU2, pDM18-HHT2- HHF2-TRP1*	Provided by Dr. Dan Gottschling
H3T11A (UCC1369)	UCC1369	*MATa ade2::hisG his3Δ200 leu2Δ0 lys2Δ0 met15Δ0 trp1Δ63 ura3Δ0 adh4::URA3-TEL-VIIL ADE2-TEL-VR , (hht2-hhf2)Δ::MET15 (hht1-hhf1)Δ::LEU2, pDM18- HHT2(T11A)-HHF2-TRP1*	In this study
H3K79A (UCC1369)	UCC1369	*MATa ade2::hisG his3Δ200 leu2Δ0 lys2Δ0 met15Δ0 trp1Δ63 ura3Δ0 adh4::URA3-TEL-VIIL ADE2-TEL-VR , (hht2-hhf2)Δ::MET15 (hht1-hhf1)Δ::LEU2, pDM18- HHT2(K79A)-HHF2-TRP1*	In this study
H3T11A H3K79A (UCC1369)	UCC1369	*MATa ade2::hisG his3Δ200 leu2Δ0 lys2Δ0 met15Δ0 trp1Δ63 ura3Δ0 adh4::URA3-TEL-VIIL ADE2-TEL-VR , (hht2-hhf2)Δ::MET15 (hht1-hhf1)Δ::LEU2, pDM18- HHT2(T11A K79A)-HHF2-TRP1*	In this study
Dot1-FLAG WT H3 (YBL)	S288C	*MATa his3Δ200 leu2Δ1 ura3-52, trp1Δ63 lys2-128δ (hht1- hhf1)Δ::LEU2, (hht2-hhf2)Δ::HIS3 Ty912 35-lacZ::his4 pDM18-HHT2-HHF2-TRP1 DOT1-3xFLAG::KAN*	In this study
Dot1-FLAG H3T11A (YBL)	S288C	*MATa his3Δ200 leu2Δ1 ura3-52, trp1Δ63 lys2-128δ (hht1- hhf1)Δ::LEU2, (hht2-hhf2)Δ::HIS3 Ty912 35-lacZ::his4 pDM18-HHT2(T11A)-HHF2-TRP1 DOT1-3xFLAG::KAN*	In this study
Dot1-FLAG H3T11D (YBL)	S288C	*MATa his3Δ200 leu2Δ1 ura3-52, trp1Δ63 lys2-128δ (hht1- hhf1)Δ::LEU2, (hht2-hhf2)Δ::HIS3 Ty912 35-lacZ::his4 pDM18-HHT2(T11D)-HHF2-TRP1*	In this study

		DOT1-3xFLAG::KAN	
Sir2-FLAG<br>WT H3 (YBL)	S288C	*MATa his3Δ200 leu2Δ1 ura3-52, trp1Δ63 lys2-128δ (hht1- hhf1)Δ::LEU2, (hht2-hhf2)Δ::HIS3 Ty912 35-lacZ::his4 pDM18-HHT2-HHF2-TRP1 SIR2-3xFLAG::KAN*	In this study
Sir2-FLAG<br>H3T11A (YBL)	S288C	*MATa his3Δ200 leu2Δ1 ura3-52, trp1Δ63 lys2-128δ (hht1- hhf1)Δ::LEU2, (hht2-hhf2)Δ::HIS3 Ty912 35-lacZ::his4 pDM18-HHT2(T11A)-HHF2-TRP1 SIR2-3xFLAG::KAN*	In this study
Sir2-FLAG<br>H3T11D (YBL)	S288C	*MATa his3Δ200 leu2Δ1 ura3-52, trp1Δ63 lys2-128δ (hht1- hhf1)Δ::LEU2, (hht2-hhf2)Δ::HIS3 Ty912 35-lacZ::his4 pDM18-HHT2(T11D)-HHF2-TRP1 SIR2-3xFLAG::KAN*	In this study
Sir2-FLAG<br>H3K79A (YBL)	S288C	*MATa his3Δ200 leu2Δ1 ura3-52, trp1Δ63 lys2-128δ (hht1- hhf1)Δ::LEU2, (hht2-hhf2)Δ::HIS3 Ty912 35-lacZ::his4 pDM18-HHT2(K79A)-HHF2-TRP1 SIR2-3xFLAG::KAN*	In this study
Sir2-FLAG<br>H3T11A H3K79A<br>(YBL)	S288C	*MATa his3Δ200 leu2Δ1 ura3-52, trp1Δ63 lys2-128δ (hht1- hhf1)Δ::LEU2, (hht2-hhf2)Δ::HIS3 Ty912 35-lacZ::his4 pDM18-HHT2(T11AK79A)-HHF2-TRP1, SIR2-3xFLAG::KAN*	In this study
Sir3-FLAG<br>WT H3 (YBL)	S288C	*MATa his3Δ200 leu2Δ1 ura3-52, trp1Δ63 lys2-128δ (hht1- hhf1)Δ::LEU2, (hht2-hhf2)Δ::HIS3 Ty912 35-lacZ::his4 pDM18-HHT2-HHF2-TRP1 SIR3-3xFLAG::KAN*	In this study
Sir3-FLAG<br>H3T11A (YBL)	S288C	*MATa his3Δ200 leu2Δ1 ura3-52, trp1Δ63 lys2-128δ (hht1- hhf1)Δ::LEU2, (hht2-hhf2)Δ::HIS3 Ty912 35-lacZ::his4 pDM18-HHT2(T11A)-HHF2-TRP1 SIR3-3xFLAG::KAN*	In this study
Sir3-FLAG<br>H3T11D (YBL)	S288C	*MATa his3Δ200 leu2Δ1 ura3-52, trp1Δ63 lys2-128δ (hht1- hhf1)Δ::LEU2, (hht2-hhf2)Δ::HIS3 Ty912 35-lacZ::his4 pDM18-HHT2(T11D)-HHF2-TRP1 SIR3-3xFLAG::KAN*	In this study
Set1-FLAG<br>WT H3 (YBL)	S288C	*MATa his3Δ200 leu2Δ1 ura3-52, trp1Δ63 lys2-128δ (hht1- hhf1)Δ::LEU2, (hht2-hhf2)Δ::HIS3 Ty912 35-lacZ::his4 pDM18-HHT2-HHF2-TRP1 SET1-3xFLAG::KAN*	In this study
Set1-FLAG<br>H3T11A (YBL)	S288C	*MATa his3Δ200 leu2Δ1 ura3-52, trp1Δ63 lys2-128δ (hht1- hhf1)Δ::LEU2, (hht2-hhf2)Δ::HIS3 Ty912 35-lacZ::his4 pDM18-HHT2(T11A)-HHF2-TRP1 SET1-3xFLAG::KAN*	In this study
Set1-FLAG<br>H3T11D (YBL)	S288C	*MATa his3Δ200 leu2Δ1 ura3-52, trp1Δ63 lys2-128δ (hht1- hhf1)Δ::LEU2, (hht2-hhf2)Δ::HIS3 Ty912 35-*	In this study

		lacZ::his4 pDM18-HHT2(T11D)-HHF2-TRP1 SET1-3xFLAG::KAN	
WT H3 (YBL)/ATG8pGFP-Atg8	S288C	*MATa his3Δ200 leu2Δ1 ura3-52, trp1Δ63 lys2-128δ (hht1- hhf1)Δ::LEU2, (hht2-hhf2)Δ::HIS3, Ty912 35-lacZ::his4, pDM18-HHT2-HHF2-TRP1, ATG8p-GFP-ATG8-URA3*	In this study
H3T11A (YBL)/ATG8pGFP-Atg8	S288C	*MATa his3Δ200 leu2Δ1 ura3-52, trp1Δ63 lys2-128δ (hht1- hhf1)Δ::LEU2, (hht2-hhf2)Δ::HIS3, Ty912 35-lacZ::his4, pDM18-HHT2(T11A)-HHF2-TRP1, ATG8p-GFP-ATG8-URA3*	In this study
H3K79A (YBL)/ATG8pGFP-Atg8	S288C	*MATa his3Δ200 leu2Δ1 ura3-52, trp1Δ63 lys2-128δ (hht1- hhf1)Δ::LEU2, (hht2-hhf2)Δ::HIS3, Ty912 35-lacZ::his4, pDM18-HHT2(K79A)-HHF2-TRP1, ATG8p-GFP-ATG8-URA3*	In this study
H3T11A H3K79A (YBL)/ATG8pGFP-Atg8	S288C	*MATa his3Δ200 leu2Δ1 ura3-52, trp1Δ63 lys2-128δ (hht1- hhf1)Δ::LEU2, (hht2-hhf2)Δ::HIS3, Ty912 35-lacZ::his4, pDM18-HHT2(T11AK79A)-HHF2-TRP1, ATG8p-GFP-ATG8-URA3*	In this study
WT	BY4741	*MATa his3Δ1 leu2Δ0 met15Δ0 ura3Δ0*	Open Biosystems
pyk1-ts (*cdc19-1*)	S288C		Yeast Conditional Temperature Sensitive
dot1Δ	BY4741	*MATa his3Δ1 leu2Δ0 met15Δ0 ura3Δ0 dot1Δ::KAN*	Open Biosystems
set1Δ	BY4741	*MATa his3Δ1 leu2Δ0 met15Δ0 ura3Δ0 set1Δ::KAN*	Open Biosystems
sir2Δ	BY4741	*MATa his3Δ1 leu2Δ0 met15Δ0 ura3Δ0 sir2Δ::KAN*	Open Biosystems
sir2Δ (UCC1369)	UCC1369	*MATa ade2::hisG his3Δ200 leu2Δ0 lys2Δ0 met15Δ0 trp1Δ63 ura3Δ0 adh4::URA3-TEL-VIIL ADE2-TEL-VR , (hht2-hhf2)Δ::MET15 (hht1-hhf1)Δ::LEU2, pDM18-HHT2- HHF2-TRP1, sir2Δ::KAN*	In this study
set1Δ (UCC1369)	UCC1369	*MATa ade2::hisG his3Δ200 leu2Δ0 lys2Δ0 met15Δ0 trp1Δ63 ura3Δ0 adh4::URA3-TEL-VIIL ADE2-TEL-VR , (hht2-hhf2)Δ::MET15 (hht1-hhf1)Δ::LEU2, pDM18-HHT2- HHF2-TRP1, set1Δ::KAN*	In this study
H3T11A *set1Δ* (UCC1369)	UCC1369	*MATa ade2::hisG his3Δ200 leu2Δ0 lys2Δ0 met15Δ0 trp1Δ63 ura3Δ0 adh4::URA3-TEL-VIIL ADE2-TEL-VR , (hht2-hhf2)Δ::MET15 (hht1-hhf1)Δ::LEU2, pDM18-HHT2(T11A)- HHF2-TRP1, set1Δ::KAN*	In this study
sas2Δ (UCC1369)	UCC1369	*MATa ade2::hisG his3Δ200 leu2Δ0 lys2Δ0 met15Δ0 trp1Δ63 ura3Δ0 adh4::URA3-TEL-VIIL ADE2-TEL-VR , (hht2-hhf2)Δ::MET15 (hht1-hhf1)Δ::LEU2, pDM18-HHT2- HHF2-TRP1, sas2Δ::KAN*	In this study
H3T11A *sas2Δ*	UCC1369	*MATa ade2::hisG his3Δ200 leu2Δ0 lys2Δ0 met15Δ0*	In this study

(UCC1369)		*trp1Δ63 ura3Δ0 adh4::URA3-TEL-VIIL ADE2-TEL-VR , (hht2-hhf2)Δ::MET15 (hht1-hhf1)Δ::LEU2, pDM18-HHT2(T11A)- HHF2-TRP1, sas2Δ::KAN*	
pyk1-ts dot1Δ	BY4741	*cdc19-1 dot1Δ::URA3*	In this study
Dot1-FLAG	BY4741	*MATa his3Δ1 leu2Δ0 met15Δ0 ura3Δ0 DOT1-3xFLAG::KAN*	In this study
Set1-FLAG	BY4741	*MATa his3Δ1 leu2Δ0 met15Δ0 ura3Δ0 SET1-3xFLAG::KAN*	In this study
Dot1-Myc	BY4741	*MATa his3Δ1 leu2Δ0 met15Δ0 ura3Δ0 DOT1-13xMYC::HIS3*	In this study
Dot1-TAP	BY4741	*MATa his3Δ1 leu2Δ0 met15Δ0 ura3Δ0 DOT1-TAP ::HIS3*	In this study
Dot1-Myc *pyk1-ts*	BY4741	*cdc19-1 DOT1-13xMYC::HIS3*	In this study
HHT2-FLAG	BY4741	*MATa his3Δ1 leu2Δ0 met15Δ0 ura3Δ0 ,HHT2-3xFLAG::KAN*	In this study
HHT2-FLAG Sir2-Myc *pyk1-ts*	BY4741	*cdc19-1 SIR2-13xMYC::HIS3 ,HHT2-3xFLAG::KAN*	In this study
HHT2-FLAG Sir2-Myc *dot1Δ*	BY4741	*MATa his3Δ1 leu2Δ0 met15Δ0 ura3Δ0, SIR2-13xMYC::HIS3 dot1Δ::URA3, HHT2-3xFLAG::KAN*	In this study
Sir2-Myc	BY4741	*MATa his3Δ1 leu2Δ0 met15Δ0 ura3Δ0 SIR2-13xMYC::HIS3*	In this study
Sir2-Myc *pyk1-ts*	BY4741	*cdc19-1 SIR2-13xMYC::HIS3*	In this study
Sir3-Myc *pyk1-ts*	BY4741	*cdc19-1 SIR3-13xMYC::HIS3*	In this study
Sir2-Myc *dot1Δ*	BY4741	*MATa his3Δ1 leu2Δ0 met15Δ0 ura3Δ0, SIR2-13xMYC::HIS3 dot1Δ::URA3*	In this study
ATG8p-GFP-Atg8	BY4741	*MATa his3Δ1 leu2Δ0 met15Δ0 ura3Δ0, ATG8p-GFPATG8-URA3*	In this study
pyk1-ts ATG8p-GFP-Atg8	BY4741	*cdc19-1 ATG8p-GFP-ATG8-URA3*	In this study
dot1Δ ATG8p-GFP-Atg8	BY4741	*MATa his3Δ1 leu2Δ0 met15Δ0 ura3Δ0 dot1Δ::KAN ATG8p-GFP-ATG8-URA3*	In this study
pyk1-ts dot1Δ ATG8p-GFP-Atg8	BY4741	*cdc19-1 dot1Δ::URA3 ATG8p-GFP-ATG8-URA3*	In this study
Reb1-TAP	BY4741	*MATa his3Δ1 leu2Δ0 met15Δ0 ura3Δ0 REB1-TAP::HIS3*	In this study
Reb1-FLAG	BY4741	*MATa his3Δ1 leu2Δ0 met15Δ0 ura3Δ0 REB1-3xFLAG::KAN*	In this study
Dot1-Myc Reb1-FLAG	BY4741	*MATa his3Δ1 leu2Δ0 met15Δ0 ura3Δ0 REB1-3xFLAG::KAN, DOT1-13xMYC::HIS3*	In this study
WT tTA	R1158	*URA3::CMV-tTA MATa his3-1 leu2-0 met15-0*	Purchase from GE

TetO$_7$-REB1	R1158	*pREB1::kanR-tetO7-TATA URA3::CMV-tTA MATa his3-1 leu2-0 met15-0*	Purchase from GE
Dot1-Myc *TetO$_7$-REB1*	R1158	*MATa URA3::CMV-tTA his3-1 leu2-0 met15-0 ura3-1 pREB1::kanR-tetO7-TATA URA3::CMV-tTA, DOT1-13xMYC::HIS3*	In this study
TetO$_7$-PGI1	R1158	*pPGI1::kanR-tetO7-TATA URA3::CMV-tTA MATa his3-1 leu2-0 met15-0*	In this study
TetO$_7$-ENO2	R1158	*PENO2::kanR-tetO7-TATA URA3::CMV-tTA MATa his3-1 leu2-0 met15-0*	In this study
acs2-ts	BY4741	*MATa his3Δ1 leu2Δ0 met15Δ0 ura3Δ0 acs2::HygMX [pHT215,acs2-Ts1-CEN-URA3]*	In this study
WT (Empty vector)	S288C	*MATa ade2::hisG his3Δ200 leu2Δ0 lys2Δ0 met15Δ0 trp1Δ63 ura3Δ0 adh4::URA3-TEL-VIIL ADE2-TEL-VR , (hht2-hhf2)Δ::MET15 (hht1-hhf1)Δ::LEU2, pDM18-HHT2- HHF2-TRP1, pTEF-HIS3*	In this study
WT (*pTEFpro-SIR2*)	S288C	*MATa ade2::hisG his3Δ200 leu2Δ0 lys2Δ0 met15Δ0 trp1Δ63 ura3Δ0 adh4::URA3-TEL-VIIL ADE2-TEL-VR , (hht2-hhf2)Δ::MET15 (hht1-hhf1)Δ::LEU2, pDM18-HHT2- HHF2-TRP1, pTEF-SIR2-HIS3*	In this study
H3T11A H3K79A (Empty vector)	S288C	*MATa ade2::hisG his3Δ200 leu2Δ0 lys2Δ0 met15Δ0 trp1Δ63 ura3Δ0 adh4::URA3-TEL-VIIL ADE2-TEL-VR , (hht2-hhf2)Δ::MET15 (hht1-hhf1)Δ::LEU2, pDM18- HHT2(T11AK79A)-HHF2-TRP1, pTEF-HIS3*	In this study
H3T11A H3K79A (*pTEFpro-SIR2*)	S288C	*MATa ade2::hisG his3Δ200 leu2Δ0 lys2Δ0 met15Δ0 trp1Δ63 ura3Δ0 adh4::URA3-TEL-VIIL ADE2-TEL-VR , (hht2-hhf2)Δ::MET15 (hht1-hhf1)Δ::LEU2, pDM18- HHT2(T11AK79A)-HHF2-TRP1, pTEF-SIR2-HIS3*	In this study
HHY168		*MATα tor1-1 fpr1::NAT, RPL13A-2×FKBP12::TRP1 pFA6a-2×FKBP12-TRP1*	Provided by Dr. Ulrich K Laemmli
Pyk1-FRB		*MATα tor1-1 fpr1::NAT, RPL13A-2×FKBP12::TRP1 pFA6a-2×FKBP12-TRP1, PYK1-FRB::KAN*	In this study

Supplementary Table 2 List of oligonucleotides used in this study

Gene name	Sequence
**qRT-PCR and ChIP-qPCR**	
ACTIN	TCGAACAAGAAATGCAAACCG<br>GGCAGATTCCAAACCCAAAAC
ATG3	TTGCTAGATAAGGTTCGTGTGG<br>CAAGTATTGGTCTACCCGTAACG
ATG7	TCCCTGTTTTCAAAGACCCTC<br>GAGAACAGCCCTTTTAAACTCG
ATG9	AGCAACTTCCCTTTACCAGAC<br>GTCAGACTCAGGAACACGTAAG
ATG10	GAGAAGAGAACACCATGAA<br>TTGTCGAGTAGGTATTAAAC
ATG11	AACTCCCCTAATTCCAACGAC<br>AACGCGAAAGATCTACGTCTG
ATG12	GGAACGGCAATGGAAAGATC<br>TCAACTTGCTGGTCGACAG
ATG14	CAAGATGAAGTGTAGGTCCGTC<br>CATGAGGTCCTGTGACTGTTG
ATG15	CGAAGACCAAGAACCCCTATG<br>TCTCTTTCCTTCATCCGCTTC
ATG16	CAATCCACAAACAGATAGCATGG<br>GTCATGGCTGACAATGTTGC
ATG17	AGGAGAAAGGATGTGGCAAAC<br>TTTACCGGGCCAGATTGTC
ATG20	GAGTAAGCCAATATTTATCGAT<br>ATGAGGTGGTTCAGCCTTT
ATG21	AGAACCTGAGCCGTAACTTTG<br>TGGTTGGGAAATTCGTCTGG
ATG5-P	AGCATGCTCAGAAGTGCGAA<br>ACATCATAGGTTTCCTATCTCCGT
ATG5	ACCAGGTAAAGGATGTTCTCAC<br>TGCGATGGGAATGATAGTTGG
ATG8-P	ACCCGTGAAATCATAGCACAT<br>ACCAAATATTTTTGCCGCCG
ATG8	ACCTTACCGTAGGGCAATTTG<br>CCCGTCCTTATCCTTGTGTTC
ATG23-P	AGTTCAAGTGATTATTTTCGTTTTCTTGT<br>CTTCTTCACTTTATTTTGTTACCTTATAGAA
ATG23	CTCCATAGCGAAAGTACCACTG<br>CGACCCTTTATGGCTTTTGTG
URA3	CAGAATTGTCATGCAAGGGC<br>GTAACCTTCATCTCTTCCACCC

YFR057W	TGATATTTGGACCTACTAGTGTCTATAG<br>GCTTGGCGGTGTCTTTAATG
YCR106W	CGCCTTAGGGTTATTATACAATGC<br>CGCTCTCAAAGAGTGAAATGTCC
COS8	CCGTTCTACCTCAAGATGTTTTCCG<br>CCAGGAACAGGACAAGAAGTGAAAC
SEO1	GGTTTAGAGGGATGGAGATGG<br>TCCTCGCCAACCTAATTTCATC
COS4	CCTTCTCGAACCCTTCTCTTTG<br>TCTTCAATGTAAGGGACGGAATC
YEL077C	CAAGTTTTCCGCATGTCTGG<br>TCGTCTTTTGCCTCCCATTC
YIR042C	CAGGAGATATGAGTGGAAGTGTG<br>GAAAACCATTGAGTGTCGCG
PAU15	AGCCACTACCACTCTATCTCC<br>ATTTCAACTGGGTAGGTCTCAC
YOL166W	TGCCATACTCACCTTCACTTG<br>GGAGTGGAATGTGAAAGTAGGG
PMA1	ACACTTTCGTTGGTAGAGCTG<br>AGAAACAAGCAGTCCAGACC
PYK1	CCCAATCCCACCAAACCAC<br>TTCTACCAGCGGAGATGACCTT
YAR009C	AACTACATCCTGAGCAACCG<br>GTATGAGTTGACGGAGGTGTG
YBL113C	CAAGTTTTCCGCATGTCTGG<br>TCGTCTTTTGCCTCCCATTC
COS3	GGATGGGACGAAATTGCAAG<br>GCAGATAGAACGCGGTAGAAG
YHL050C	CAAGTTTTCCGCATGTCTGG<br>TCGTCTTTTGCCTCCCATTC
YLL067C	CCAAGTTTTCCGCATGTCTG<br>TCGTCTTTTGCCTCCCATTC
YRF1-5	CCAAGTTTTCCGCATGTCTG<br>TCGTCTTTTGCCTCCCATTC
COS2	GGATGGGACGAAATTGCAAG<br>GCAGATAGAACGCGGTAGAAG
PAU19	AGCCACTACCACTCTATCTCC<br>CAATCTCAACTGGGTAGGTCTC
COS12	ACAATTCCACGCTACCCAAG<br>CCACATTAATAGCCAAGTGCG
PAU16	AGCCACTACCACTCTATCTCC<br>ATTTCAACTGGGTAGGTCTCAC
YRF1-3	TTACCCCATCTAAAGTGCCG<br>ACAACCACACCTCCGAAATC

YHR219W	CCAAGTTTTCCGCATGTCTG<br>TCGTCTTTTGCCTCCCATTC
GEX2	GCGTTTGTATGTGGGTTTGG<br>TGGACAGTTGAAAGTAAGGAGTG
UBA2-1	TTTTGGATTTAGAAATTACATGACGTTC<br>AACCCTTCACCATTCGGATC
UBA2-2	CTTGAAAGGGGAATGATGTTTATTTG<br>ACCATTTTTCTTTCCTTTTAATTTCCTC
FAR3-1	ACGATAATCTCAAGCACAGGG<br>AATAAAAGTTGAAGCGGTGGC
FAR3-2	AAGATTCCAGAGATTACAGCCG<br>GGTTTGCTATTTCACGTCTGC
GCD6-1	CTCAGTGGTTAGAGCTTCGTG<br>GGTCGCGTTGCTTTTAAAGG
GCD6-2	TGTCCTAAGTGGCAAAGAACTG<br>GTCCCTTTCTTGTACTCTCTCAG
SAT4-1	AATCATACTCGAAGGGTAGTTGG<br>AGGGTTGAACTCACGATCTTG
SAT4-2	AAAGAGCCAGGTGTAGGAATG<br>AGCATTTTATCTCCCTTCCCC
REB1	CTGCGGCTGATACTGACGAT<br>ACCTTGAAGTCGATGGCGTT
ENO2	CTCCAAACATTCAAACCGCTG<br>GTCGTACTTACCGTCCTTGAAG
PGI1	TCTGGTCGGCTATTGGTTTG<br>TTGGGTGAAGTGGTTGTCG
TEL V R	GTAGATGGTGAAAAAGTGGTATAACG<br>CCCATAAAGCCCACGATTATCC
TEL VIII L	ATGTGGATAATCGTGGGCG<br>CATTATGCACGGCACTTGC
TEL XII L	GTGACAGCGAGAGTAAAGGTAG<br>TGTCTAACACCATCCAGCATG
TEL XIV L	AGAAGTTGTAGGCTAAGCGC<br>AGGATTCTGTTCGTTGCTCAG
TEL XV R	CGTGTGCCTATGCCATATCAG<br>CATCCGCTACTCACCATACTG
TEL XIII-L 5.5K	GATGCTATATGTCCCTACGGC<br>TGTTAGGATTGTGTTAGGGTGTG
TEL XIII-L 8.5K	TGAAATTCTGTATTGCAAACCCC<br>TTACAATGTGGCAAAATGAGACC
TEL XIII-L 11.5K	GGAAATAGGCTCTCGTGTCTC<br>TGGATTTACCCAGGAACATCTG
TEL XII-L 0.2K	ATCTACCTCTACTCTCGCTGTC

	CGTACATGAGGGCTATTTAGGG
TEL XII-L 1.5K	CCAAGTTTTCCGCATGTCTG<br>TCGTCTTTTGCCTCCCATTC
TEL XII-L 5K	TGTCTAACACCATCCAGCATG<br>GTGACAGCGAGAGTAAAGGTAG
TEL XII-L 6.5K	CCAAGTTTTCCGCATGTCTG<br>TCGTCTTTTGCCTCCCATTC
TEL XII-L 11K	ACACAGTACCTCAACACAACC<br>AGGTAGGGTAATGGAGGGTAG
TEL XII-L 13K	ACTATCGCAACCCAGAAATTTTG<br>GCGTCACTTCTAAGAACAAGATTG
TEL XII-L 14K	GGGTTTCTGTATCCGCGTAAG<br>ACGATGAAATTGAAGATGAGAAGTG
TEL XII-L 16K	TTACCTTCGTCAGCACCTTC<br>TCTACCGTGTACCAGATCCTTC
TEL XII-L 25K	ACGTCCCTGACTAAGCTTTTC<br>CTCACGAGCATATGGATACAGAG
TEL XII-L 30K	TGAAGGAATGGGTGGAGTTG<br>GTAGCGATGAAGGCACAAAC
TEL XIII-R 0.3K	GTATGGTGAGTAGCAGATGGTG<br>CCGTTACCCTCCAATTACCC
TEL XIII-R 1.7K	AGCCACTACCACTCTATCTCC<br>CAATCTCAACTGGGTAGGTCTC
TEL XIII-R 4.4K	ACCCCTTTGTTATTCCTGTTCC<br>GTAAACCTCCGAACCCATCC
TEL XIII-R 9.6K	ATCAGTTTCGAGAGCAACCC<br>GGAGCTTGTGCGGTTATGTA
TEL II-L 0.3K	TGTTGCACGGCAGTAGC<br>GTTGTCTCTTACCCGGATGTTC
TEL II-R 0.2K	CCATGTCCTACTCACTGTACTG<br>GTAGAAGTGAAGGTGAGTGTG
TEL VII-L 0.3K	ACCCTACCACTCTAATCCCAC<br>GTGTGAGAGTAGGGTAAGTTTGAG
TEL VII-R 0.2K	GAGACAAGTGGGAAAGAGTAGG<br>CCCTAAATAGCCCTCATGTACG
TEL VIII-L 0.2K	GCTTTTGGTTGAACATCCGG<br>AATAGCCCTAAATAGCCCTCATG
TEL VIII-R 0.2K	CACACCCACACTTTTCACATC<br>GGCTATGTAGAAGTGCTGTAGG
TEL IX-R 0.3K	CTACCATTACCCTACCATCCAC<br>AAGGTGAGTATGGCATGTGG
TEL XI-L 0.15K	GTGGGAGTGGTATGGTTGAG<br>CTCCAAACCTACCCTCACATTAC

TEL XI-R 0.4K	AGCCATCTGTTACTCACCATAC<br>GACCGATCAGAATACAAGTGAGG
TEL XII-R 0.1K	AATAGCCCTCATGTACGTCTC<br>GCAGTAGCGAGAGACAAGTG
