# Supplementary material for: Author Correction: SESAME-catalyzed H3T11 phosphorylation inhibits Dot1-catalyzed H3K79me3 to regulate autophagy and telomere silencing
Source: Nat Commun. 2026 Oct 19;17:10916. doi: 10.1038/s41467-026-78553-2 (PMC13680606; doi:10.1038/s41467-026-78553-2)
Supplement: Supplementary file 1 [file 41467_2026_78553_MOESM1_ESM.pdf]

Original Fig. 4

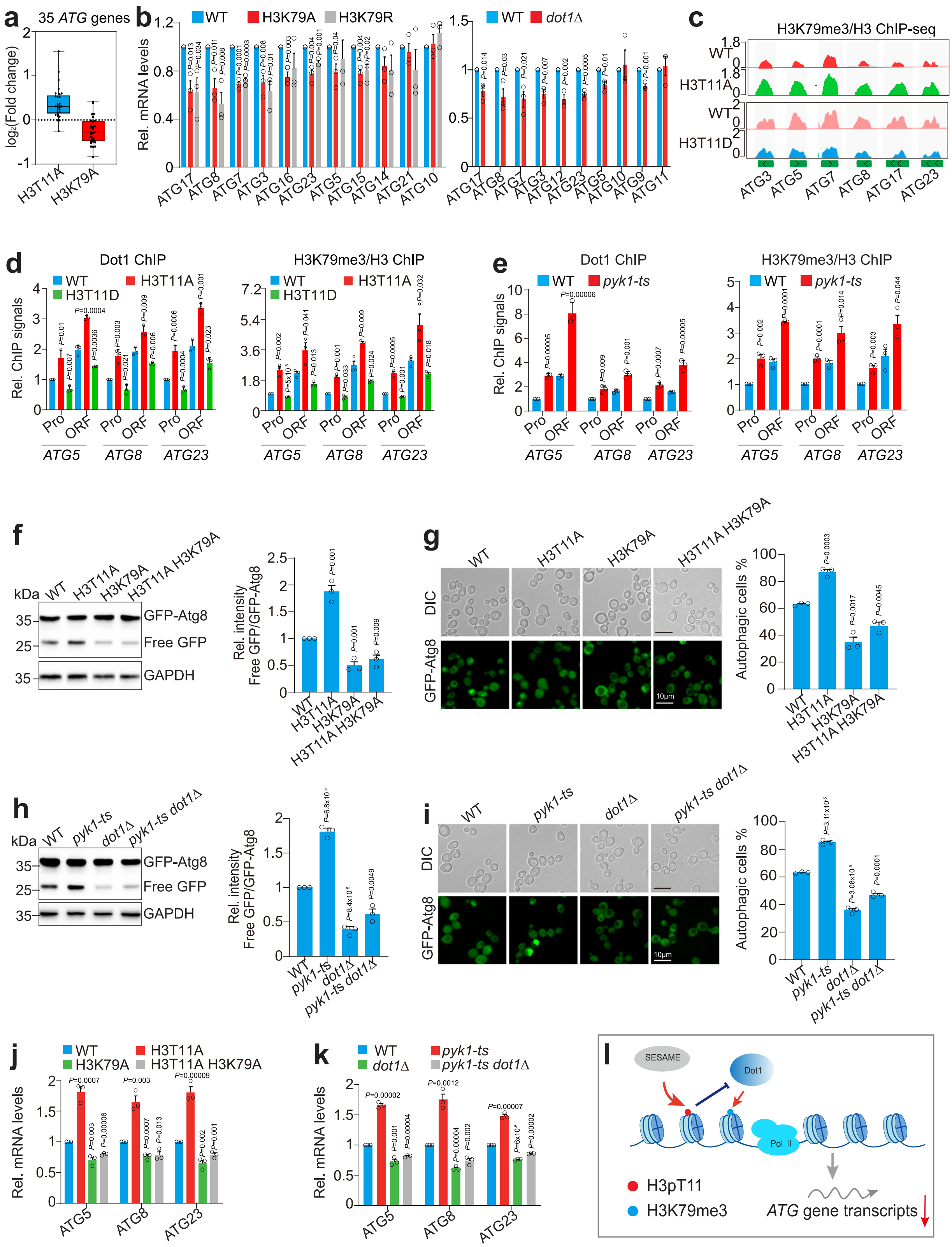

Original Fig. 6

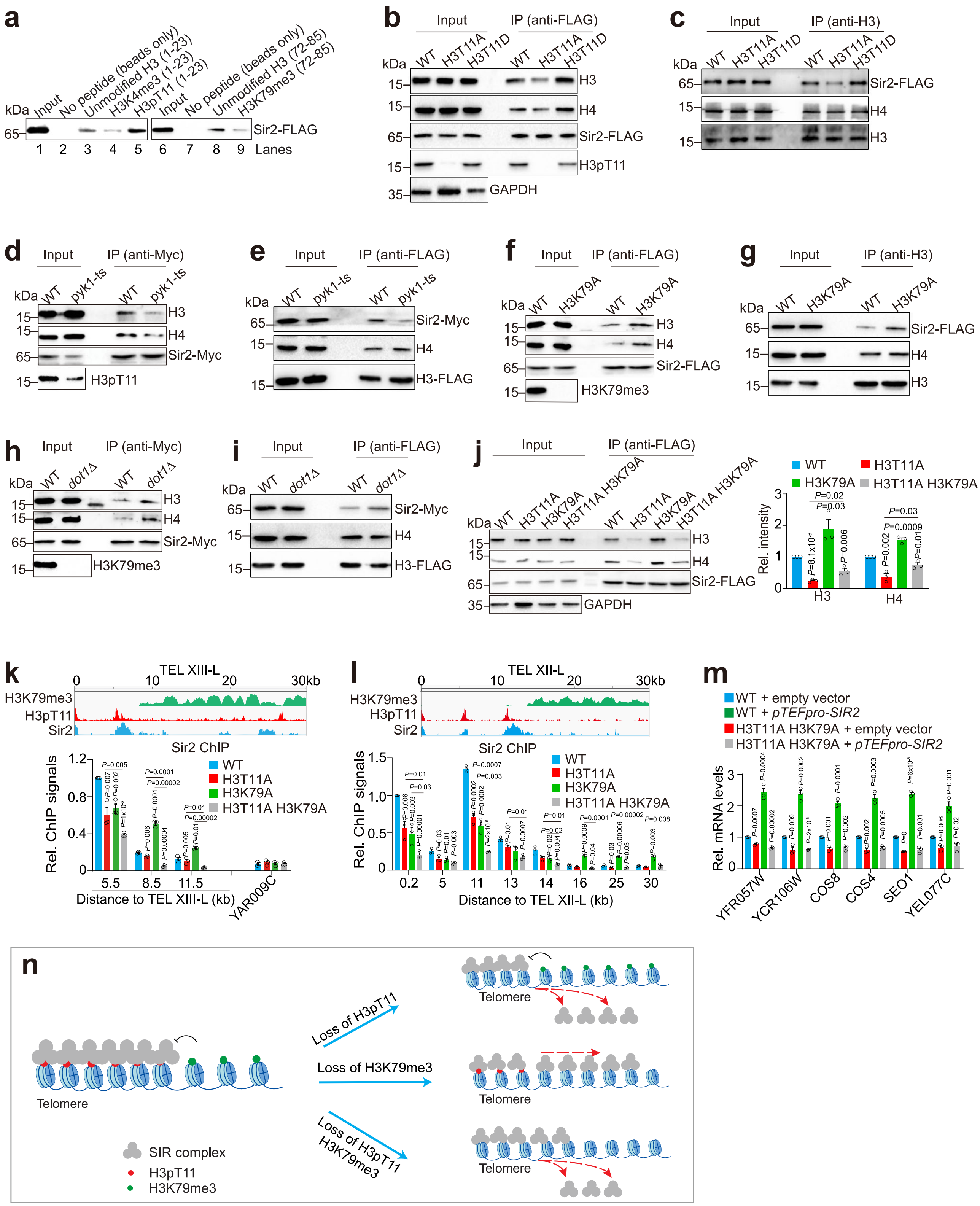

Supplementary Fig. 8

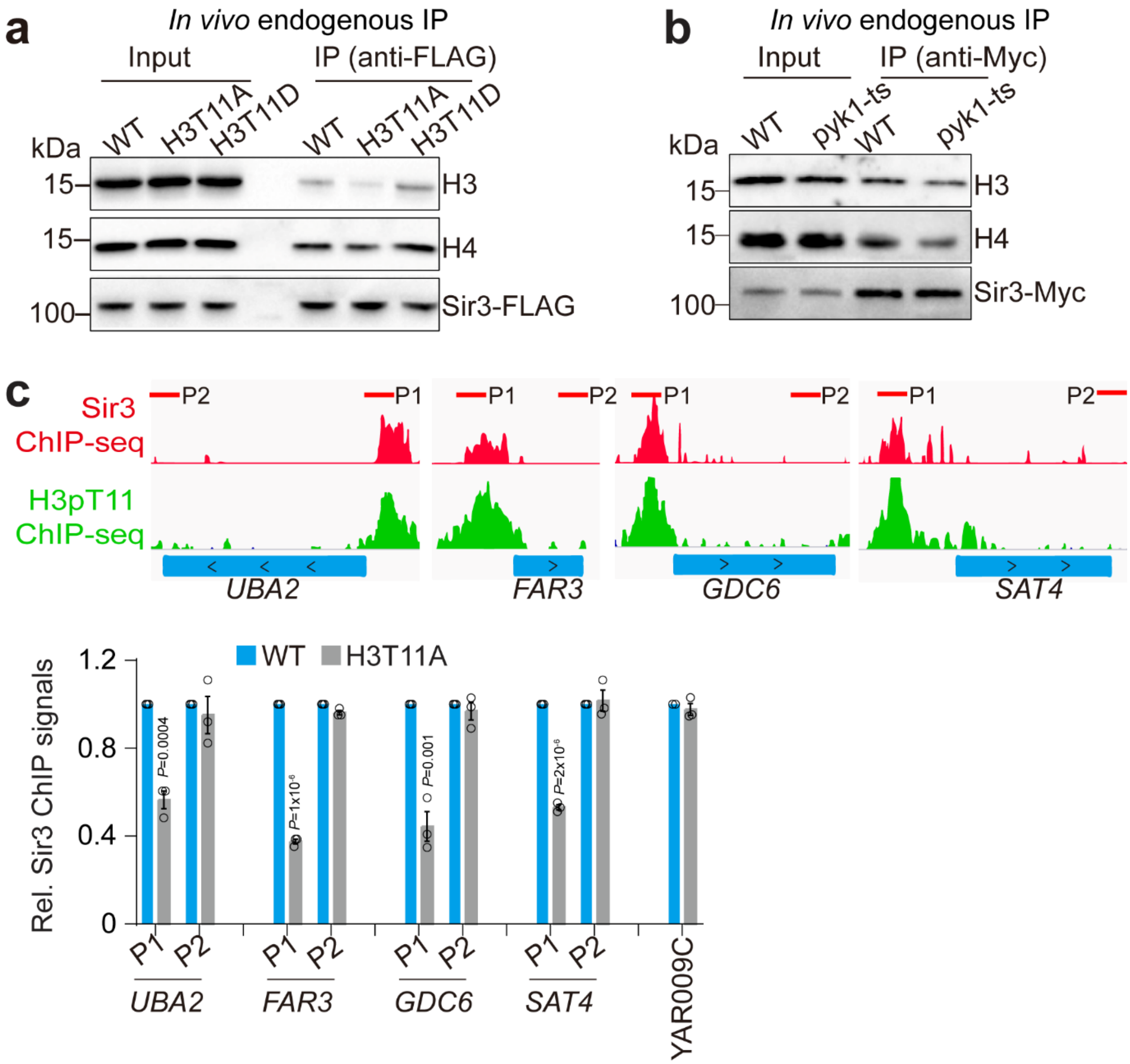

Supplementary Fig. 8. SESAME-catalyzed H3pT11 promotes the binding of SIR complex at telomere regions.

a-b, *In vivo* endogenous Co-IP assays showing SESAME-catalyzed H3pT11 facilitates SIR complex binding to chromatin. **a**, Endogenously expressed Sir3-FLAG was immunoprecipitated from WT, H3T11A and H3T11D mutants with anti-FLAG beads. **b**, Endogenously expressed Sir3-Myc was immunoprecipitated from WT and *pyk1-ts* mutant with anti-Myc beads. Cells were grown in YPD medium at 39 °C for 2 hr.

c, ChIP-qPCR analysis of Sir3 occupancy at indicated regions in WT and H3T11A mutant. The primers used to amplify the regions were indicated at the top of ChIP-seq tracks in red lines.

For Supplementary Fig. 8**c**, data represent the mean ± SE; n=3 biologically independent experiments. Two-sided *t*-tests were used for statistical analysis. For Supplementary Fig. 8**a-b**, a typical example of two biologically independent replicates was shown.
